# Astrocyte-targeted gene delivery of interleukin 2 specifically increases brain-resident regulatory T cell numbers and protects against pathological neuroinflammation

**DOI:** 10.1038/s41590-022-01208-z

**Published:** 2022-05-26

**Authors:** Lidia Yshii, Emanuela Pasciuto, Pascal Bielefeld, Loriana Mascali, Pierre Lemaitre, Marika Marino, James Dooley, Lubna Kouser, Stijn Verschoren, Vasiliki Lagou, Hannelore Kemps, Pascal Gervois, Antina de Boer, Oliver T. Burton, Jérôme Wahis, Jens Verhaert, Samar H. K. Tareen, Carlos P. Roca, Kailash Singh, Carly E. Whyte, Axelle Kerstens, Zsuzsanna Callaerts-Vegh, Suresh Poovathingal, Teresa Prezzemolo, Keimpe Wierda, Amy Dashwood, Junhua Xie, Elien Van Wonterghem, Eline Creemers, Meryem Aloulou, Willy Gsell, Oihane Abiega, Sebastian Munck, Roosmarijn E. Vandenbroucke, Annelies Bronckaers, Robin Lemmens, Bart De Strooper, Ludo Van Den Bosch, Uwe Himmelreich, Carlos P. Fitzsimons, Matthew G. Holt, Adrian Liston

**Affiliations:** 1 VIB-KU Leuven Center for Brain & Disease Research, Leuven, Belgium; 2 KU Leuven, Department of Microbiology, Immunology and Transplantation, Leuven, Belgium; 3 KU Leuven - Department of Neurosciences, Leuven, Belgium; 4 Swammerdam Institute for Life Sciences, Faculty of Science, University of Amsterdam, Amsterdam, Netherlands; 5 Immunology Programme, The Babraham Institute, Babraham Research Campus, Cambridge, United Kingdom; 6 Cardio & Organ Systems (COST), Biomedical Research Institute (BIOMED), Hasselt University, Diepenbeek, Belgium; 7 VIB Bio-Imaging Core, Leuven, Belgium; 8 KU Leuven, Faculty of Psychology, Laboratory of Biological Psychology, Leuven, Belgium; 9 VIB-KU Leuven Center for Brain & Disease Research, Electrophysiology Expertise Unit, Leuven, Belgium; 10 VIB Center for Inflammation Research, Ghent, Belgium; 11 Department of Biomedical Molecular Biology, Faculty of Sciences, Ghent University, Ghent, Belgium; 12 Toulouse Institute for Infectious and Inflammatory diseases (INFINITY), INSERM UMR1291, CNRS UMR 5051, Toulouse, France; 13 KU Leuven, Department of Imaging and Pathology, Biomedical MRI, Leuven, Belgium; 14 University Hospitals Leuven, Department of Neurology, Leuven, Belgium; 15 Dementia Research Institute, University College London, London, United Kingdom; 16 Instituto de Investigaçāo e Inovaçāo em Saúde (i3S), University of Porto, Porto, Portugal

**Keywords:** Neuroimmunology, Immunotherapy, Interleukins, Regulatory T cells

## Abstract

The ability of immune-modulating biologics to prevent and reverse pathology has transformed recent clinical practice. Full utility in the neuroinflammation space, however, requires identification of both effective targets for local immune modulation and a delivery system capable of crossing the blood–brain barrier. The recent identification and characterization of a small population of regulatory T (T_reg_) cells resident in the brain presents one such potential therapeutic target. Here, we identified brain interleukin 2 (IL-2) levels as a limiting factor for brain-resident T_reg_ cells. We developed a gene-delivery approach for astrocytes, with a small-molecule on-switch to allow temporal control, and enhanced production in reactive astrocytes to spatially direct delivery to inflammatory sites. Mice with brain-specific IL-2 delivery were protected in traumatic brain injury, stroke and multiple sclerosis models, without impacting the peripheral immune system. These results validate brain-specific IL-2 gene delivery as effective protection against neuroinflammation, and provide a versatile platform for delivery of diverse biologics to neuroinflammatory patients.

## Abstract (web summary)

Liston and colleagues design a gene-delivery system to specifically target astrocytes in the central nervous system to express IL-2 and thereby expand/maintain T_reg_ cells to suppress neuroinflammation.

## Main

Acute central nervous system (CNS) trauma is the leading cause of death and disability for people under the age of 45 years^1^. Although the causes of trauma are diverse, the common end result is substantial neuronal damage, or neuronal loss, in the affected region. This is thought to underlie the cognitive, sensorimotor function and personality changes typically seen in patients^1^. To date, drug treatments adopting a ‘neuro-centric’ approach have failed to deliver notable clinical benefits for the treatment of CNS injury^1,2^, indicating that this approach is too narrow. Acute CNS injury is now recognized as triggering a multicellular response, involving CNS-resident immune cells (microglia and astroglia) alongside infiltration of peripheral immune cells to the brain parenchyma^3^. While there is evidence to support a neuroprotective effect of immune activation during the initial CNS response, prolonged activation invariably becomes neurotoxic^3,4^. The involvement of the immune system allows immune-modulating biologics to emerge as a key therapeutic option. However, adoption of immune-modulating biologics in the neuroinflammatory clinical space first requires identification of biologics with effective anti-inflammatory potential in the CNS, coupled with parallel development of delivery systems capable of crossing the blood–brain barrier.

IL-2 is a high-potential immune-modulating biologic, based on its capacity to support the survival and proliferation of T_reg_ cells. T_reg_ cells possess potent immunoregulatory capacity, and are common in the blood and secondary lymphoid organs, with a small population resident in the healthy CNS^5^. While the capacity of IL-2 supplementation to expand circulating T_reg_ cells and inhibit neuroinflammation has been well demonstrated, these effects can be attributed to T_reg_ cell function in secondary lymphoid organs. For example, more severe pathology is observed following systemic T_reg_ cell depletion in mouse models of neuroinflammation, such as the experimental autoimmune encephalomyelitis (EAE) model of multiple sclerosis (MS)^6,7^, or models of stroke^8,9^ and traumatic brain injury (TBI)^10^. In neuroinflammatory diseases, such as EAE, where T cells trigger the inflammatory cascade^11^, T_reg_ cell depletion can enhance peripheral priming and infiltration of neuropathogenic T cells, regardless of any putative role for tissue-resident T_reg_ cells in the brain. Even in injury-driven neuroinflammation, such as stroke or TBI, the systemic T_reg_ cell depletion typically used to assess function also drives massive peripheral inflammation^12^, with potential pathological consequences^10^. The involvement of CNS-resident T_reg_ cells, as opposed to peripheral-resident T_reg_ cells, in the control of neuroinflammatory pathology thus remains obscured. This unknown remains one of the key limitations in the clinical utility of IL-2 in the neurology space, with the need to define potential for CNS-based impact, as opposed to systemic effects.

The functional distinction between systemic and CNS-based IL-2 delivery is critical for any therapeutic exploitation in neuroinflammatory disease. Treatments that rely on systemic IL-2 provision to drive expansion of circulating T_reg_ cells as a mechanism to control CNS inflammation would cause parallel systemic immune suppression and are, therefore, unlikely to see wide adoption in the clinic. By contrast, CNS-specific increases in IL-2 could allow treatment of neuroinflammation without inducing peripheral immunosuppression. Here, we demonstrate the highly efficacious control of neuroinflammation by CNS-based IL-2, using a synthetic biological circuit to drive local production of IL-2 while leaving the peripheral immune system intact. Furthermore, we provide a solution to the biologic delivery problem for the brain, with an adeno-associated virus (AAV)-based therapeutic delivery system capable of providing exquisite temporal and spatial control over biologic production. The demonstrated neuroprotection in four independent neuroinflammatory models provides a clear pathway to clinical exploitation of brain-specific IL-2 gene delivery, and a platform for the delivery of diverse biologics, potentially suitable for broad classes of neuroinflammatory disease and injury.

## Results

### IL-2 in brain drives T_reg_ cell expansion and neuroprotection

**Fig. 1:**
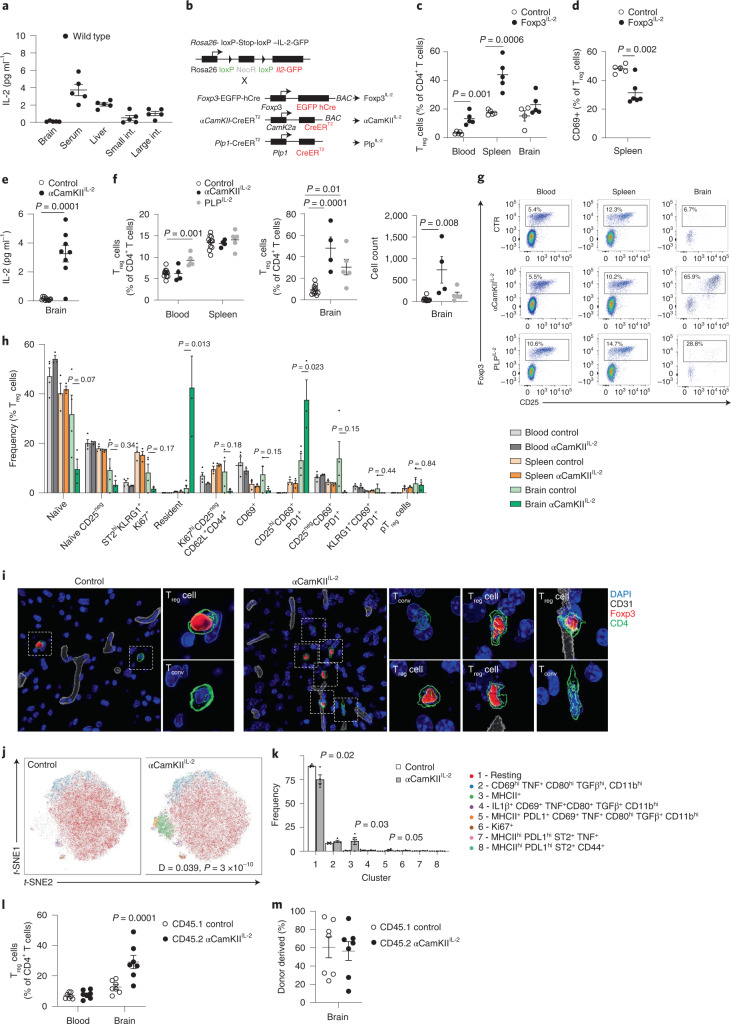
Local expression of IL-2 drives a brain-specific expansion of T_reg_ cells. **a**, IL-2 levels detected by ELISA (*n* = 5 per group). **b**, Schematic of transgenic systems for IL-2 expression. **c**,**d**, Frequency of Foxp3^+^ cells within CD4^+^ T cells (*n* = 5, 5; **c**), and CD69^+^ cells within T_reg_ cells (*n* = 5, 6; **d**). **e**, IL-2 levels detected by ELISA (*n* = 11, 8). **f**, Frequency of Foxp3^+^ cells within CD4^+^ T cells (*n* = 8, 4, 5). Absolute number of Foxp3^+^ cells. **g**, Representative flow cytometry plots for **f** indicating Foxp3 and CD25 coexpression. **h**, Brain, spleen and blood from wild-type and αCamKII^IL-2^ mice were compared by flow cytometry (*n* = 4, 3). **i**, Surface-rendered image of a T_reg_ cell in the midbrain. A representative picture of three individual mouse samples is shown. Scale bar, 10 µm. **j**, Brains from wild-type and αCamKII^IL-2^ mice were compared by flow cytometry (*n* = 4, 4; 64,927 cells plotted). *t*-distributed stochastic neighbor embedding (*t*-SNE) of microglia built on key markers (CD64, MHCII, PD-L1, CD80, CX3CR1 and CD45). **k**, Cluster quantification. **l**, CD45.1 mice parabiosed to tamoxifen-treated CD45.2 αCamKII^IL-2^ mice. Percentage of T_reg_ cells within the CD4^+^ T cell population in the blood and brain of parabiotic pairs (*n* = 7). **m**, Proportion of T_reg_ cells from the CD45.1 or CD45.2 donor in the brain of parabiotic pairs. Data from **a**, **c**–**f**, **h** and **k**–**m** are shown as the mean ± s.e.m. Statistical analyses were performed using an unpaired two-tailed Student’s *t*-test (**d** and **e**), unpaired two-tailed Student’s *t*-test with multiple-test correction (**c**, **h** and **k**), two-way analysis of variance (ANOVA; **e** and **m**) and one-way ANOVA (**f**). All experiments except **a**, **j** and **k** were repeated independently (≥ twice). pT_reg_, peripheral T_reg_ cell. Source data

**Extended Data Fig. 1:**
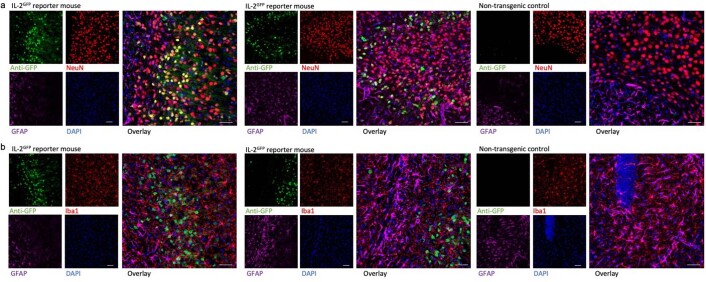
IL-2 reporter expression in neurons. Healthy perfused mouse brains from IL-2^GFP^ mice and non-transgenic controls assessed for GFP reporter expression by immunohistochemistry. **a**, anti-GFP IL-2 reporter (green), NeuN (red), GFAP (purple) and DAPI (blue). Single and combined channel confocal images of GFP-expressing NeuN+ cells in the mid-brain, compared to non-transgenic control. **b**, anti-GFP IL-2 reporter (green), Iba1 (red), GFAP (purple) and DAPI (blue). Single and combined channel confocal images of GFP-expressing cells in the mid-brain, compared to non-transgenic control. All images are of representative sections from three mice (n = 3/group). Scale bar, 50 µm.

**Extended Data Fig. 2:**
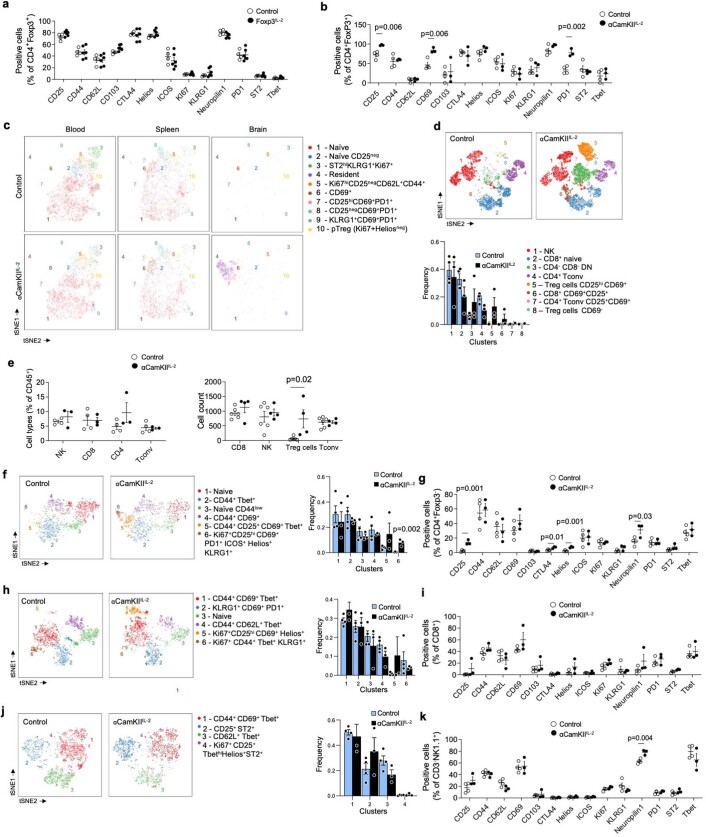
Brain regulatory T cell-specific effects of neuronal IL-2 production. **a**, Spleen from wildtype and Foxp3IL-2 mice were compared by flow cytometry (n = 4, 6). Frequency of key markers (CD25, CD44, CD62L, CD103, CTLA4, Helios, ICOS, Ki67, KLRG1, Neuropilin1, PD1, ST2, Tbet) on Treg cells in spleen. **b**, Brain from wildtype and αCamKII^IL-2^ mice were compared by high-dimensional flow cytometry (n = 4, 3). Frequency quantification of key markers on Treg cells. **c**, tSNE of blood, spleen and brain Treg cells built on key markers. Colors indicate annotated FlowSOM clusters, with quantification in Supplementary Fig. 5 f. tSNE run on samples pooled post-acquisition, with quantification performed on individual samples (n = 4, 3). The residential cluster is characterized as CD25hiCD69 + PD1 + CD103 + . **d**, tSNE of total leukocytes from brain of wildtype and αCamKII^IL-2^ mice, built on lineage markers with quantification. tSNE run on samples pooled post-acquisition, with quantification performed on individual samples (n = 4, 3). **e**, NK, CD4 and CD8 T cells, (left) as a proportion of CD45 + CD11b^−^ cells in the brain (n = 4, 3)., and (right) in absolute numbers, together with Treg cells (n = 6, 4). **f**, tSNE of brain CD4 conventional T cells built on key markers (CD62L, CD44, CD103, CD69, CD25, PD-1, Nrp1, ICOS, KLRG1, ST2, Ki67, Helios, T-bet, CTLA4). tSNE run on samples pooled post-acquisition, with quantification performed on individual samples (n = 4, 3). Colors indicate annotated FlowSOM clusters, with quantification and **g**, frequency of marker expression. **h**, tSNE of brain CD8 T cells built on key markers. tSNE run on samples pooled post-acquisition, with quantification performed on individual samples (n = 4, 3). Colors indicate annotated FlowSOM clusters, with quantification and **i**, frequency of marker expression. **j**, tSNE of brain NK cells built on key markers (t-SNE run on samples pooled post-acquisition, with quantification performed on individual samples (n = 4, 3). Colors indicate annotated FlowSOM clusters, with quantification and **k**, frequency of marker expression. Data are displayed as mean ± s.e.m. (**a**,**b**,**d**-**k**). Statistical analyses were performed using multiple unpaired two-tailed Student’s t-test. Source data

The potent capacity of T_reg_ cells to prevent inflammation makes increased IL-2 expression (with its proven ability to expand the T_reg_ cell population^13^) an attractive therapeutic strategy for neuroinflammatory pathology. In peripheral organs, the main source of IL-2 is activated CD4^+^ conventional T (T_conv_) cells. A negative feedback loop between T_reg_ cells and activated CD4^+^ T cells normally limits IL-2 provision, creating a stable T_reg_ cell niche^14^. In the brain, by contrast, IL-2 levels are ~tenfold lower than the serum (Fig. 1a), with the most common IL-2-producing cell type being neurons (Extended Data Fig. 1). As T_reg_ cells undergo elevated apoptosis during IL-2-starvation^13^, we sought to overcome IL-2 as a limiting factor using a transgenic model of IL-2 autocrine expression by T_reg_ cells (Fig. 1b). By effectively bypassing IL-2 silencing, *Foxp3*^*Cre*^ *Rosa*-*IL-2* mice exhibit a profound expansion of peripheral T_reg_ cell numbers (Fig. 1c). Notably, however, expansion does not occur in the brain (Fig. 1c), with the expansionary effect of increased IL-2 production in the periphery primarily observed on T_reg_ cells of the circulating phenotype, rather than the brain-resident CD69^+^ population (Fig. 1d and Extended Data Fig. 2a). These results limit the practical utility of peripheral IL-2 dosing to treat neuroinflammatory pathology.

**Extended Data Fig. 3:**
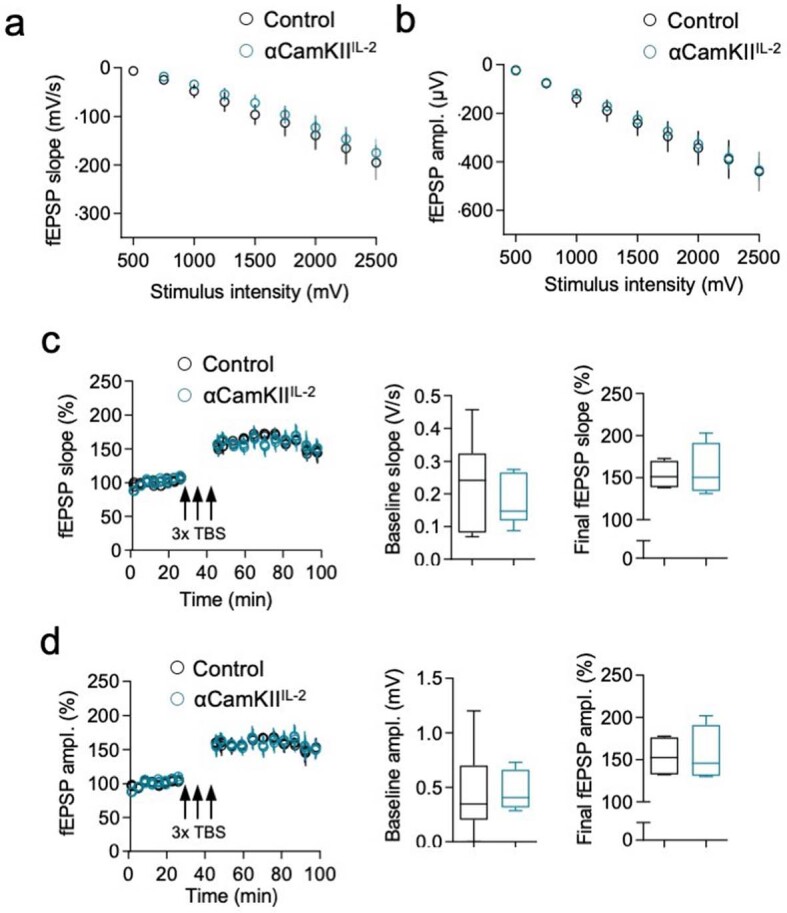
Normal long-term potentiation in αCamKII^IL-2^ mice. Field excitatory post-synaptic potentials (fEPSPs) were recorded from Schaffer collateral-CA1 neuronal synapses in brain slices from wildtype and αCamKII^IL-2^ littermates. Input-output curves were recorded for each slice by applying single-stimuli ranging from 500 to 2750 mV with 250 mV increments. **a** Slope and **b** amplitude were analyzed (n = 4,4). Long-term potentiation (LTP) was induced by applying three high frequency trains (theta-burst stimulation (TBS): 100 stimuli; 100 Hz) with 5 minutes intervals between trains. After baseline determination, fEPSPs were measured for 55 minutes. Changes in the **c** slope and **d** amplitude have been analyzed across time. Boxplots represent quantification of the baseline (left) (Amplitude: Minimum (0, 02845), 25% Percentile, (0.2020, 0.3137), Median (0.3471, 0.4084), 75% Percentile (0.6997, 0.6627), Maximum (1.203, 0.7302); Slope: Minimum (0.06911, 0.08740), 25% Percentile (0.08134, 0.1182), Median (0.2409, 0.1463), 75% Percentile (0.3236, 0.2653), Maximum (0.4574, 0.2747) and final LTP (right) (Amplitude: Minimum (132.1, 130), 25% Percentile (132.4, 130.8), Median (152.7, 145.7), 75% Percentile (176.5, 191.1), Maximum (177.9, 202); Slope: Minimum (138.2, 131.3), 25% Percentile (138.7, 134.4), Median (151.3, 150.3), 75% Percentile (170.3, 191.6), Maximum (173.0, 203.2)). Mean ± s.e.m. (n = 4,4). Statistical analyses were performed using unpaired, nonparametric Mann–Whitney U-test. Source data

**Extended Data Fig. 4:**
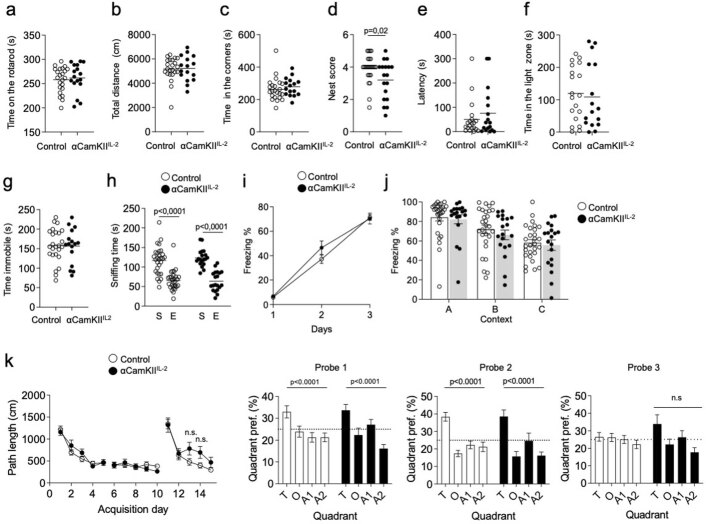
Normal behavior in mice with expanded brain regulatory T cells. Behavioral assessment of αCamKII^IL-2^ and littermate control mice. **a**, Time spent on the rod, average of 4 repeated tests of 300 seconds (n = 23, 17). **b**, Open field, total distance moved and **c** time spent in the corners (n = 23, 16). **d**, Nest building scoring (n = 24, 18). **e**, Light-dark test latency to enter light zones and **f** time spent in the light zone (n = 20, 17). **g**, Time immobile during forced swim test (n = 24, 16). **h**, Sociability test trials to monitor the interaction with a stranger mouse (S) compared to an empty chamber (E) (n = 28, 18). **i**, Freezing behavior over time during context acquisition conditioning (n = 28, 18). **j**, Contextual discrimination during generalization test (n = 28, 18). **k**, Spatial learning in the Morris water maze. Path length to finding the hidden platform (n = 28, 18), probe tests after 5 days and 10 days and after reversal learning (n = 28, 18). All data are displayed as mean ± s.e.m. All experiments were repeated two times independently. Statistical analyses were performed using Two-way ANOVA with multiple comparison correction **(h, i, j)** or Two-way Repeated Measures (RM) ANOVA **(k)**. Source data

We therefore sought to exploit the impermeable nature of the blood–brain barrier through ectopic expression of IL-2 by brain cells. Using a transgenic expression system to drive cell-type-specific expression of low levels of IL-2, we could activate IL-2 expression in oligodendrocytes or neurons using Cre drivers (Fig. 1b,e). While the PLP-Cre driver in oligodendrocytes gave additional systemic effects, αCamKII-Cre-mediated IL-2-expression in neurons resulted in brain-specific expansion of T_reg_ cells (Fig. 1f,g). FlowSOM cluster analysis of T_reg_ cells demonstrated that, compared to T_reg_ cells from the wild-type brain, T_reg_ cells in the αCamKII^IL^^-2^ brain were substantially depleted for the naïve cluster (CD26L^hi^CD44^lo^) and highly significantly enriched in a cluster expressing multiple residential markers (CD69, CD103, ST2 and KLRG1) and a CD25^hi^CD69^+^PD-1^+^ cluster (Fig. 1h and Extended Data Fig. 2b,c). Together, this implies enrichment in resident T_reg_ cells, with the elevation in CD25 and programmed cell death protein 1 (PD-1) known to be induced by IL-2 exposure^15^. Using imaging-based approaches, elevated numbers of T_reg_ cells were observed beyond the vasculature (Fig. 1i, Supplementary Fig. 1 and Supplementary Videos 1 and 2), distributed across large areas of brain (Supplementary Fig. 2). Co-staining with the vascular basement membrane marker laminin α4 produced similar results, with elevated numbers of T_reg_ cells sparsely distributed across entire coronal sections in αCamKII^IL-2^ mice (Supplementary Fig. 3 and Supplementary Resource 1). By flow cytometry, increases in T_reg_ cell numbers were observed across the brain regions assessed (Supplementary Fig. 4). Only minor changes were observed in the non-T_reg_ cell brain-resident leukocyte populations of α^-2^ mice, with limited shifts in population frequency and marker expression (Extended Data Fig. 2d–k). Single-cell sequencing demonstrated that, within the CD4^+^ T cell compartment, only the residential T_reg_ cell cluster was affected in frequency by brain IL-2 provision (Supplementary Fig. 5c). Aside from this numerical expansion, no major transcriptional alterations were observed in the T_reg_ cell population (Supplementary Fig. 6). Brain T_reg_ cell expansion did not alter the electrophysiology of neurons (Extended Data Fig. 3), and resulted in no major adverse behavioral changes (Extended Data Fig. 4) or excess mortality (aged to 18 months with 90% survival versus 93% in the control littermates; *n* = 11, 15).

To investigate the transcriptional changes induced in the targeted population, namely CD4^+^ T cells and microglia (the primary immunological cell type in the brain), we turned to single-cell sequencing. Bulk CD4^+^ T cells and CD11b^+^ myeloid cells were sorted and T_reg_ cells and microglia identified based on the expression of canonical markers (Supplementary Fig. 7). Single-cell sequencing identified an increase in major histocompatibility complex class II (MHCII)-related gene expression in microglia, potentially enabling enhanced interaction between brain T_reg_ cells and microglia, but otherwise no major transcriptional changes were detected (Supplementary Fig. 7). Increased MHCII protein expression was validated by flow cytometry, where ~15% of microglia expressed MHCII in αCamKII^IL-2^ mice, with the microglia otherwise normal and not expressing inflammatory or activation markers (Fig. 1j,k and Supplementary Fig. 7). The exception was programmed death-ligand 1 (PD-L1), which increased on microglia in αCamKII^IL-2^ mice (Supplementary Fig. 7), and corresponded with the increase in PD-1 expression in brain T_reg_ cells (Extended Data Fig. 2). As PD-1 engagement on T_reg_ cells protects the cells from apoptosis^15^, these interaction partners may contribute to the T_reg_ cell expansion observed following IL-2 upregulation. Together, these results demonstrate a rare example of the blood–brain barrier working in favor of an intervention, with brain-specific expression of IL-2 resulting in a local expansion of the resident T_reg_ cell population without off-target impacts on peripheral T_reg_ cells.

We previously characterized the brain-resident T_reg_ cell population as a semi-transient migratory population, with the majority of seeding cells rapidly dying or leaving within days, while ~5% gain a residential phenotype and dwell for weeks^5^. To determine whether brain-delivered IL-2 primarily works through enabling more efficient seeding or through the selective expansion (or retention) of already-resident T_reg_ cells, we performed parabiosis between αCamKII^IL-2^ and wild-type mice. This creates a system whereby a single circulatory system feeds into two brains, one (wild type) with normal, limiting, levels of IL-2, and the other (αCamKII^IL-2^) with IL-2 levels elevated to those of the serum. Following equilibration of circulating cells, we found that αCamKII^IL-2^ mice, but not controls or parabiotic pairs, exhibited an increase in brain-resident T_reg_ cells (Fig. 1l). This demonstrates that the expansion is restricted to the supplemented brain niche, and is not transmitted via circulatory factors. Second, in both wild-type brains and αCamKII^IL-2^ brains, the brain-resident T_reg_ cell population demonstrated equivalent representation of host and donor T_reg_ cells (Fig. 1m). Together, these results demonstrate that brain-specific IL-2 levels are the limiting factor in controlling the population kinetics of incoming T_reg_ cells, as a niche functionally distinct from the peripheral system.

**Fig. 2:**
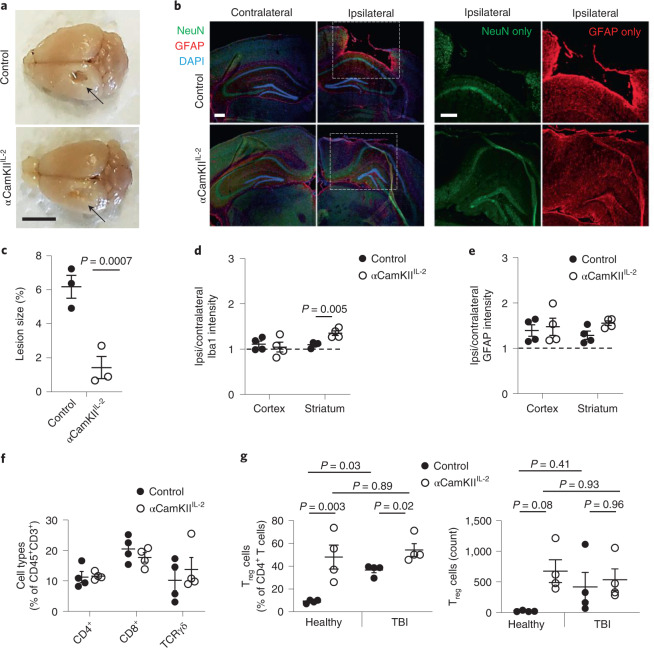
Protection from neuroinflammation following brain-specific expression of IL-2. **a**, Control littermates and αCamKII^IL-2^ mice were tamoxifen treated at 6 weeks and controlled cortical impacts to induce moderate TBI were given at 12 weeks. Mice were examined 15 d after TBI (*n* = 3, 3). Representative photos illustrating damage to the surface of the brain at the injury site. Arrow, site of impact. Scale bar, 0.5 cm. **b**, Representative immunofluorescence staining of the cortical tissue 14 d after cortical impact. GFAP (astrocytes), NeuN (neurons), DAPI (nuclei). Scale bars, 50 µm. **c**, Lesioned area, shown as the percentage of the entire hemisphere (*n* = 3, 3). **d**,**e**, Relative Iba1 (**d**) and GFAP (**e**) expression levels in the cortex and striatum (ratio of expression in ipsilateral versus contralateral hemispheres; *n* = 4, 4). **f**, TBI-induced perfused brains from wild-type and CamKII^IL-2^ mice were compared at 15 d after TBI by high-dimensional flow cytometry (*n* = 4, 4). Frequency of CD4^+^, CD8^+^ and gamma delta (γδ) T cells within CD45^+^CD11b^−^ cells. **g**, TBI-induced perfused brains from wild-type and αCamKII^IL-2^ mice were compared before TBI, or at 15 d after TBI by high-dimensional flow cytometry (*n* = 4 per group). Frequency of T_reg_ cells within CD4^+^ T cells (left) and absolute number of T_reg_ cells (right). Data from **c**–**g** are shown as the mean ± s.e.m. Data are presented as individual biological replicates, *n* = 3 or 4 mice per group. Statistical analyses were performed using unpaired two-tailed Student’s *t*-test or one-way ANOVA (**g**). Source data

Having identified that brain and peripheral T_reg_ cell populations are reliant on compartmentalized IL-2 niches, we next sought to determine whether supplementing brain IL-2 could alleviate pathological neuroinflammation-driven pathology. While IL-2 and T_reg_ cell therapy have been reported in other contexts^16–19^, these approaches expanded the systemic T_reg_ cell population, and thus the effects observed cannot be definitively attributed to local CNS effects. To test whether brain-specific supplementation of IL-2 could influence neuroinjury, independent of systemic immunosuppression, we used a controlled cortical impact model of TBI to deliver an acute insult. In wild-type mice, this injury typically leads to widespread inflammatory-mediated neurodegeneration 14 d after injury (Supplementary Fig. 8). αCamKII^IL-2^ mice, by contrast, exhibited a high degree of protection against damage at the injury site (Fig. 2a), with reduced lesion size and partial preservation of neuronal tissue (Fig. 2b,c). Microgliosis was not observed in the post-TBI cortex, but increased Iba1 intensity in αCamKII^IL-2^ mice was seen in the post-TBI striatum (Fig. 2d). Astrogliosis was unaffected by the mouse genotype (Fig. 2e). Despite the partial anatomical preservation in αCamKII^IL-2^ mice, the leukocyte influx in the brain remained relatively unchanged, apart from the increased T_reg_ cell frequency already present before TBI (Fig. 2f,g and Supplementary Fig. 9). These results demonstrate proof of principle for local brain-specific IL-2 production as a potent suppressor of neuroinflammation-induced pathology.

### A dual-lock IL-2 system drives expansion of brain T_reg_ cells

**Fig. 3:**
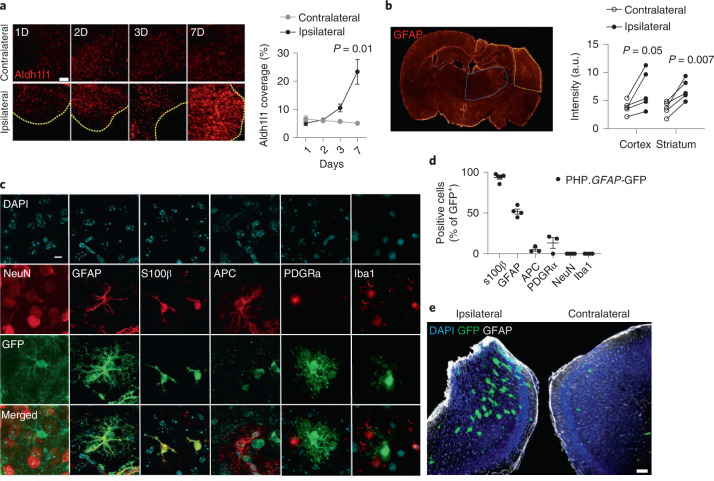
Synthetic delivery to the brain via a dual-lock gene-delivery system. **a**, Wild-type mice were given controlled cortical impacts to induce moderate TBI and examined at 1, 2, 3 and 7 d after TBI (*n* = 5). Representative images (left) and quantification (right) of astrocyte coverage in the cortex adjacent to the lesion (delineated in yellow) or corresponding contralateral cortical area, ascertained via Aldh1l1 immunostaining (*n* = 3). Scale bar, 100 µm. Statistical analysis was performed using a *t*-test with multiple-test correction. **b**, Representative staining (left) and quantified expression (right) of GFAP in the cortex (yellow) and striatum (blue), 14 d after TBI (*n* = 5), with quantification. Scale bar, 50 µm. **c**, The *GFAP* promoter restricts gene expression (as assessed using GFP scoring) to astrocytes in adult mouse brain, based on characteristic cell morphology and by immunostaining for the astrocyte-specific markers, GFAP and S100β. Off-target expression was not detected when slices were counterstained for NeuN (neurons), APC (oligodendrocytes), PDGFRα (NG2^+^ cells) and Iba1 (microglia). Scale bar, 20 µm. Data are representative of three slices from at least three mice receiving a PHP.*GFAP*-GFP (control) vector. **d**, Quantification of GFP colocalization with cell lineage markers in PHP.*GFAP*-GFP-treated mice. **e**, Wild-type mice were given controlled cortical impacts to induce moderate TBI, treated with PHP.*GFAP*-GFP and examined at 14 d after treatment. Representative image of GFP production in the ipsilateral region surrounding the impact site or the corresponding contralateral cortical area. Scale bar, 100 µm. Data from **a**, **b** and **d** are shown as the mean ± s.e.m. Statistical analyses were performed using unpaired two-tailed Student’s *t*-test with multiple-comparisons test. a.u., arbitrary units. Source data

We next sought to translate our transgenic system to a gene-delivery approach with clinical potential. To improve the biological properties of the IL-2 micro-targeting while avoiding neurons as targets, we shifted IL-2 production to astrocytes, using the *GFAP* promoter to drive IL-2 expression. *GFAP*-mediated IL-2 expression has several theoretical advantages: (1) astrocytes are efficient secretory cells, widely distributed across the brain; (2) localized astrogliosis occurs during neuroinflammatory events such as TBI (Fig. 3a), and (3) the *GFAP* promoter is more active during astrogliosis, concentrating cargo production in the inflamed region of the brain (Fig. 3b). To take advantage of astrocytes as an expression system, we designed a ‘dual-lock’ delivery system for IL-2, using AAV-PHP.B combined with a modified *GFAP* promoter capable of driving robust expression widely in astrocytes (Fig. 3c). The system combines the enhanced CNS gene delivery (brain and spinal cord) seen with the PHP.B capsid following systemic delivery^20,21^, with the secondary specificity of using a modified endogenous promoter restricted to astrocytes (*GFAP*), such that (peripheral) off-target transduction would be unable to drive cargo expression. This ‘dual-lock’ system results in astrocyte-driven cargo expression, as assessed by green fluorescent protein (GFP) expression using an AAV-PHP.B.*GFAP*-GFP (PHP.*GFAP*-GFP; Fig. 3c). Both S100β^+^GFAP^+^ and S100β^+^GFAP^−^ cortical astrocytes were able to express the transgene, with limited coexpression observed with neurons, microglia, oligodendrocytes or NG2^+^ cells (Fig. 3c,d). Elevated expression of the cargo was observed around the TBI impact site (Fig. 3e), demonstrating that the upregulation seen in endogenous promoter activity (Fig. 3b) was faithfully recapitulated by the exogenous *GFAP* promoter in the vector (Fig. 3b).

**Fig. 4:**
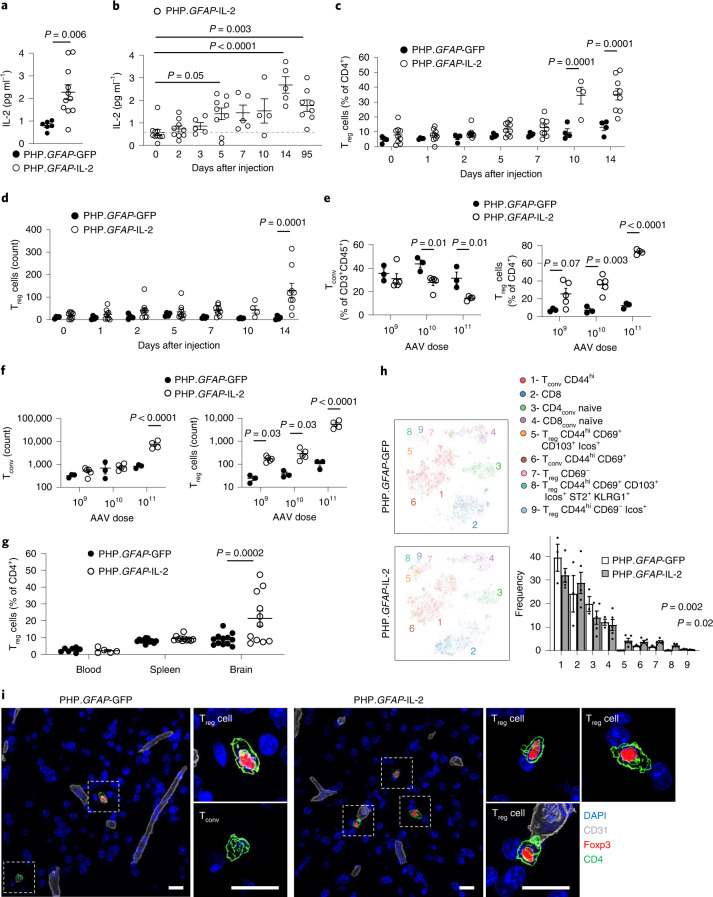
Dual-lock delivery of IL-2 to the brain expands local T_reg_ cells. **a**, IL-2 levels detected by ELISA in the brains of wild-type mice, 14 d after treatment with PHP.*GFAP*-GFP or PHP.*GFAP*-IL-2 (*n* = 6, 11). **b**, Time course of IL-2 levels in the brains of mice treated with PHP.*GFAP*-IL-2 (*n* = 10, 9, 5, 9, 5, 4, 5 and 8). **c**,**d**, Time course of T_reg_ cell expansion, as a proportion (**c**) or absolute number (**d**) of CD4^+^ T cells in the brains of mice treated with PHP.*GFAP*-GFP or PHP.*GFAP*-IL-2 (*n* = 4, 9). **e**, Wild-type mice were administered 1 × 10^9^ (*n* = 3, 5), 1 × 10^10^ (*n* = 3, 5) or 1 × 10^11^ (*n* = 3, 4) vector genomes (total dose) of PHP.*GFAP*-GFP or PHP.*GFAP*-IL-2 by intravenous injection and assessed for the frequency (**e**) or absolute number (**f**) of conventional T cells (left) and T_reg_ cells (right) in the perfused brain 14 d after treatment (*n* = 3, 5 for the 1 × 10^9^ and 1 × 10^10^ groups; *n* = 3, 4 for the 1 × 10^11^ group). **g**, Blood, spleens and perfused mouse brains from PHP.*GFAP*-GFP- and PHP.*GFAP*-IL-2-treated mice were compared by high-dimensional flow cytometry for T_reg_ cell numbers (*n* = 7, 5 blood; *n* = 12, 11 spleen and brain). **h**, *t*-SNE of CD45^+^CD11b^−^CD19^−^CD3^+^ T cells built on key markers (CD4, CD8, Foxp3, CD62L, CD44, CD103, CD69, CD25, PD-1, Nrp1, ICOS, KLRG1, ST2, Ki67, Helios and CTLA4) from perfused brains. Colors indicate annotated FlowSOM clusters; results are quantified in the bar graph (*n* = 3, 5). Mean ± s.e.m. **i**, Representative images (surface-rendered confocal sections) of T_reg_ cells in the midbrain of PHP.*GFAP*-GFP and PHP.*GFAP*-IL-2-treated mice. A representative picture of three individual mouse samples is shown. Scale bar, 10 µm. Data from **a**–**h** are shown as the mean ± s.e.m. All experiments were repeated independently (≥ twice). Statistical analyses were performed using an unpaired two-tailed Student’s *t*-test (**a** and **h**), one-way ANOVA with Dunnett’s multiple-comparisons test (**b**) or two-way ANOVA with Bonferroni correction (**c**–**g**). Source data

**Extended Data Fig. 5:**
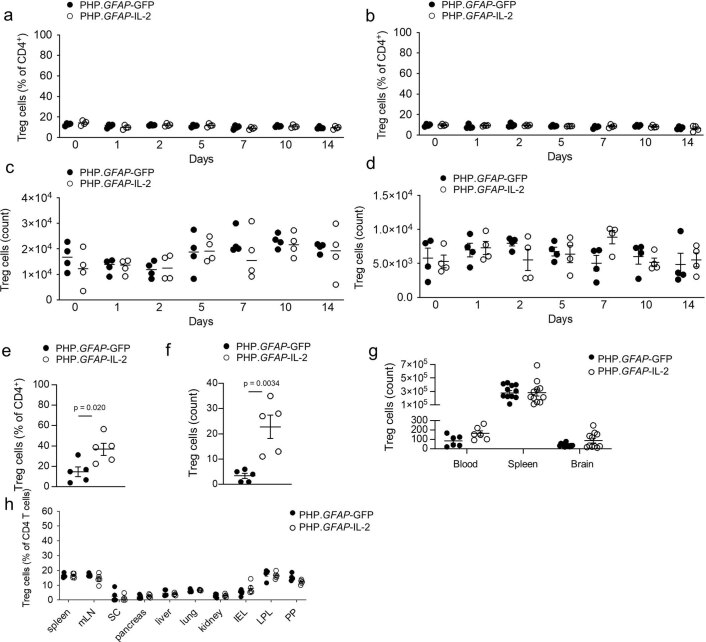
PHP.*GFAP*-IL-2 expands regulatory T cells in the brain without impacting draining lymph nodes. Mice were treated with PHP.*GFAP*-GFP or PHP.*GFAP*-IL-2 and assessed for Treg numbers by flow cytometry of perfused mice (n = 4-5, 4-9). **a**, Frequency of Treg cells, as a proportion of CD4 T cells in the superficial cervical lymph nodes (n = 4, 4), and **b** deep cervical lymph nodes (n = 4, 4). **c**, Absolute number of Treg cells in superficial cervical lymph nodes (n = 4, 4), and **d** deep cervical lymph nodes (n = 4, 4). **e**, Frequency and **f** absolute number of Treg cells in the pia mater, 14 days after PHP.*GFAP*-GFP or PHP.*GFAP*-IL-2 treatment (n = 5,5). **g**, Blood, spleen and perfused mouse brain from PHP.*GFAP*-GFP and PHP.*GFAP*-IL-2-treated mice were compared by high-dimensional flow cytometry for Treg numbers (n = 6, 6 blood; 12, 11 spleen and brain). **h**, Perfused organs from PHP.*GFAP*-GFP and PHP.*GFAP*-IL-2-treated mice were compared by flow cytometry for Treg frequency (n = 5/group). mLN, mesenteric lymph nodes; SC, spinal cord; IEL, intraepithelial leukocytes; LPL, lamina propria leukocytes; PP, Peyer’s Patch. Statistical analyses were performed using unpaired, non-parametric Mann–Whitney U-tests. Source data

**Extended Data Fig. 6:**
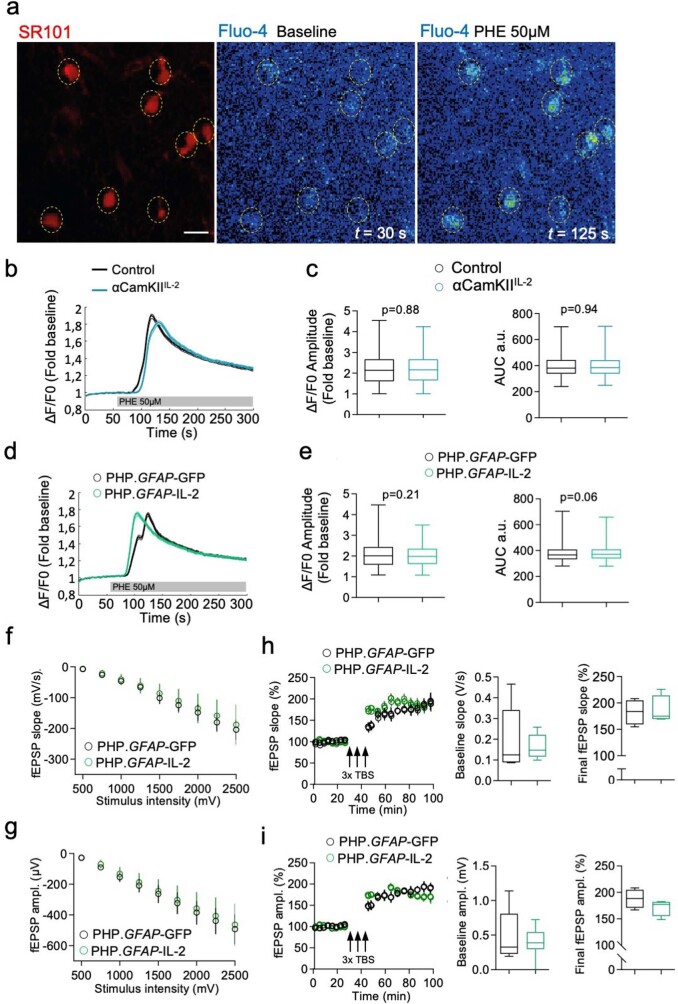
Elevated IL-2 did not produce detectable effects on astrocyte or neuron function. Astrocyte Ca^2+^ imaging in acute brain slices. **a**, Left, SR101^+^ astrocytes (dotted circles). Right, Fluo4 signal at baseline and following Phenylephrine (PHE) application. **b**, Averaged ΔF/F0 traces ± s.e.m. **c**, Boxplots of ΔF/F0 amplitude (left) and Area Under the Curve (AUC) (right). n = 3 mice/group and n_astrocytes_ = 710, 774 for control and αCamKII^IL-2^. Whiskers represent maximal and minimal values. For AUC, Minimum (240.1, 249.1), 25% Percentile (338.1, 338.7), Median (381.8, 385.1), 75% Percentile (443.8, 442), Maximum (699.3, 702.3). For ΔF/F0 amplitude, Minimum (1.006, 1.007), 25% Percentile (1.604, 1.648), Median (2.139, 2.161), 75% Percentile (2.664, 2.665), Maximum (4.542, 4.239) **d**, Averaged ΔF/F0 traces ± s.e.m. **e**, Boxplots of the ΔF/F0 amplitude (left) and AUC (right). n = 3 mice/group, and n_astrocytes_ = 609, 646 for PHP.*GFAP*-GFP and PHP.*GFAP*-IL-2, respectively. Whiskers represent maximal and minimal values. For ΔF/F0 amplitude: Minimum (1.086, 1.075), 25% Percentile (1.578, 1.617), Median (2.007, 1.980), 75% Percentile (2.431, 2.348), Maximum (4.463, 3.489). For AUC: Minimum (280.6, 279.3), 25% Percentile (332.3, 339.7), Median (366.4, 371), 75% Percentile (408.7, 409.2), Maximum (704.4, 659.5). Neuronal function was measured using fEPSPs in PHP.*GFAP*-GFP and PHP.*GFAP*-IL-2 treated mice (n = 4,4). Input-output curves were recorded by applying single-stimuli. Slope **f**, and **g**, amplitude were analyzed. LTP was induced by applying three high frequency stimulus trains (100 stimuli; 100 Hz; arrows theta burst stimulation - TBS). After baseline, fEPSPs were measured. Changes in **h**, slope and **i**, amplitude were analyzed across time. Boxplots of the baseline (left) and final LTP (right). Mean ± s.e.m. Whiskers represent the maximal and minimal values. For slope at baseline: Minimum (0,08681, 0,09894), 25% Percentile (0.08875, 0.1164), Median (0.1248, 0.1473), 75% Percentile (0.3407, 0.2212), Maximum (0.4659, 0.2584); for slope of final LTP: Minimum (154.8, 169.4), 25% Percentile (159.4, 169.7), Median (184, 174.9), 75% Percentile (204.8, 214.1), Maximum (208.3, 225.8). For amplitude at baseline, Minimum (0.1941, 0), 25% Percentile (0.2298, 0.2979), Median (0.3279, 0.3897), 75% Percentile (0.8083, 0.5444), Maximum (1.139, 0.7234). For amplitude of final LTP: Minimum (166.9, 148.7), 25% Percentile (171, 155.3), Median (188.7,177.2), 75% Percentile (204.8, 181.9), Maximum (208.4, 1828). Unpaired, non-parametric Mann–Whitney U-test. Source data

**Extended Data Fig. 7:**
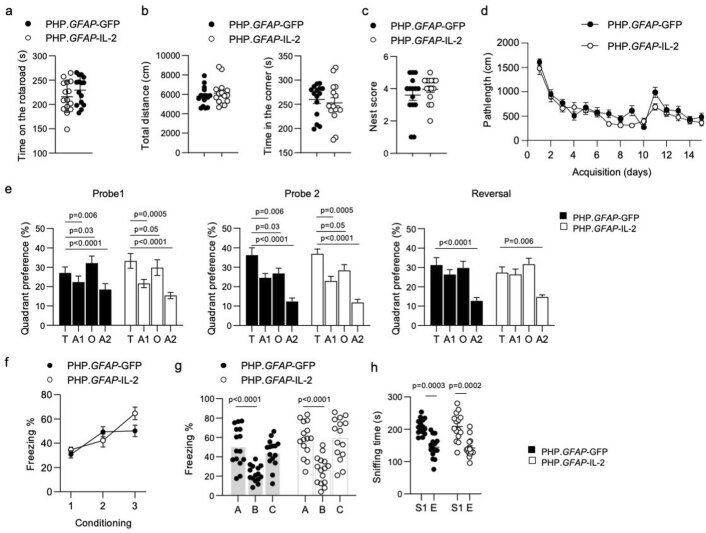
Normal behavior in PHP.*GFAP*-IL-2-treated mice. Behavioral assessment of wildtype mice treated with PHP.*GFAP*-GFP or PHP.*GFAP*-IL-2. **a**, Time spent on the rod, average of 4 repeated tests of 300 seconds (n = 15,15). **b**, Open field, total distance moved and time spent in the corners (n = 15,15). **c**, Nesting behavior (n = 15,15). **d**, Spatial learning in the Morris water maze. Path length to finding the hidden platform (n = 15,15). **e**, Probe tests after 5 days and 10 days and after reversal learning (n = 15,15). **f**, Freezing behavior over time during context acquisition conditioning (n = 15,15). **g**, Contextual discrimination during generalization test (n = 15,15). **h**, Sociability test trials to monitor the interaction with a stranger mouse (S1) compared to an empty chamber (E) (n = 15,15). Mean ± s.e.m. All experiments were repeated twice independently. Statistical analyses were performed using an unpaired two-tailed Student’s t-test. Source data

**Extended Data Fig. 8:**
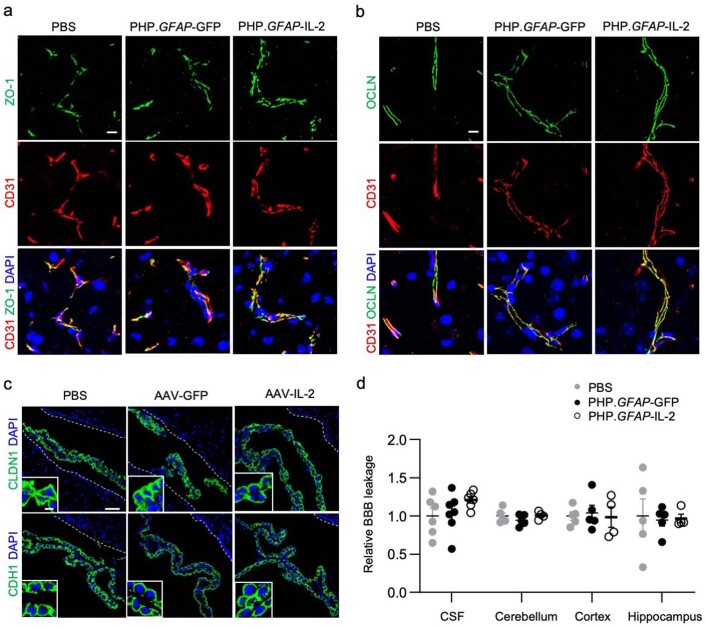
Intact blood-brain barrier integrity following PHP treatment. Wildtype mice treated with PBS, PHP.*GFAP*-GFP or PHP.*GFAP*-IL-2 were assessed at post-injection day 14 for blood-brain barrier (BBB) integrity. **a**, Histological assessment for CD31, Zonula occludens-1 (ZO-1) and DAPI or **b** CD31, Occludin (OCLN) and DAPI. Scale bar, 10 µm. **c**, Histological assessment for CLDN1 and DAPI or E-cadherin/CDH1 and DAPI. Scale bar, 50 µm. Insert scale bar, 10 µm. **d**, Mice were injected i.v. with 4 kDa FITC-dextran, followed by quantification in the cerebral spinal fluid (CSF), cerebellum, cortex and hippocampus (n = 6, 7, 6 CSF; 5, 5, 4 Cerebellum, Cortex, Hippocampus). Mean ± s.e.m. Source data

**Extended Data Fig. 9:**
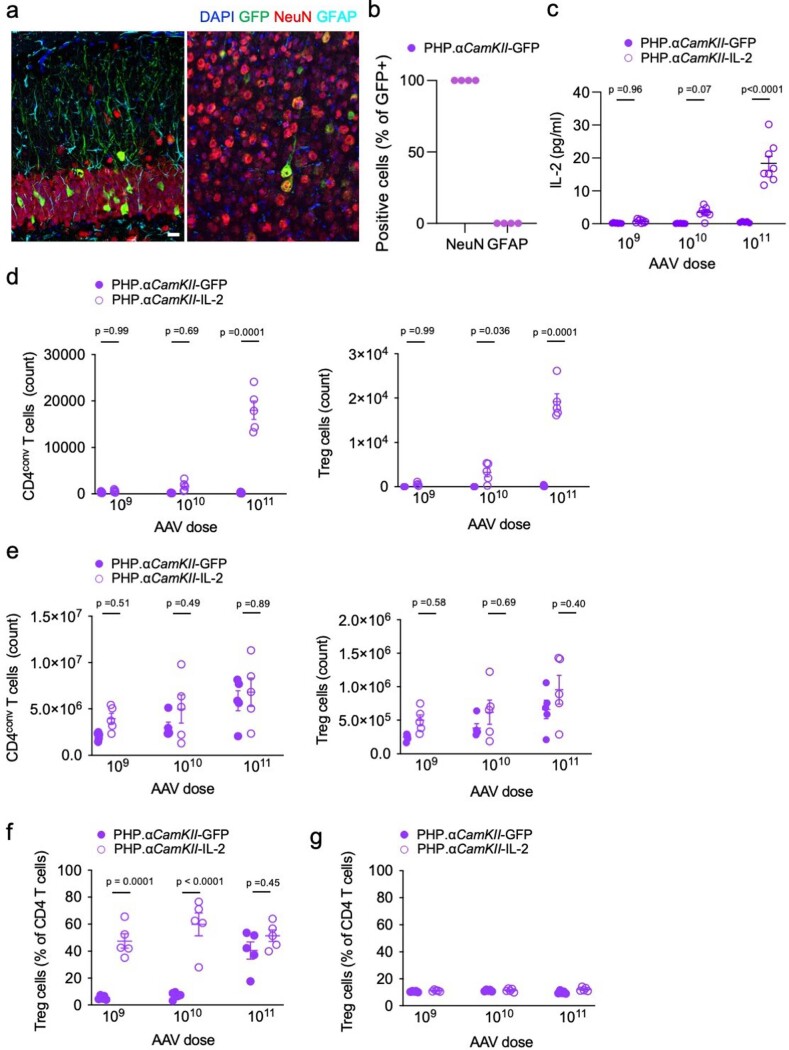
Brain-specific Treg expansion following PHP.*CamKII*-IL-2 treatment. The *CamKII* promoter restricts gene expression (as assessed using GFP scoring) to neurons (NeuN positive) in adult mouse brain. Off-target expression was not detected when slices were counter-stained for GFAP (astrocytes). Left panel, hippocampus; right panel, cortex. Scale bar, 20 µm. Data are representative images seen in 1 slice from each of 4 independent mice receiving a PHP. *CamKII*-GFP (control) vector. **b**, Quantification of GFP colocalization with NeuN and GFAP in PHP.*CamKII*-GFP-treated mice. **c**, Levels of IL-2 were measured from tissue samples obtained from wildtype mice administered with 1×10^9^, 1×10^10^ or 1×10^11^ total vector genomes of PHP.*CamKII*-GFP (control) or PHP. *CamKII*-IL-2 (n = 7,7,5,8,8,8). **d**, Wildtype mice were administered, intravenously, 1×10^9^, 1×10^10^ or 1×10^11^ vector genomes (total dose) of PHP.*CamKII*-GFP or PHP.*CamKII*-IL-2 and assessed for the number of conventional T cells (left) and Treg cells (right), 14 days after treatment (n = 5 group) in the perfused brain or **e** spleen. **f**, Treg cells as a percentage of CD4 T cells in the brain and **g** spleen. Statistical analysis was performed using two-way ANOVA with Sidak correction. Source data

Having validated the AAV-PHP.B.*GFAP* system for brain-specific expression, we sought to determine whether it could be applied to IL-2 delivery. Using AAV-PHP.B.*GFAP*-IL-2 (PHP.*GFAP*-IL-2) delivery, we observed a threefold increase in brain IL-2 production (Fig. 4a), observed over the course of 14 d (Fig. 4b). The increase in brain IL-2 concentrations was paralleled by an increase in brain T_reg_ cell frequency (Fig. 4c) and absolute number (Fig. 4d). The increase in brain T_reg_ cells was not observed in the superficial or deep cervical lymph nodes (Extended Data Fig. 5a–d), but was mirrored in the pia mater (Extended Data Fig. 5e,f). The expansion of the T_reg_ cell population was dose dependent (Fig. 4e,f), and restricted to the brain, without expansion of T_reg_ cells in the blood, spleen or other peripheral tissues (Fig. 4g and Extended Data Fig. 5g,h). The expanded T_reg_ cells were of the CD69^+^ residential phenotype (Fig. 4h), and were observed in the brain tissue beyond the vasculature (Fig. 4i, Supplementary Fig. 10 and Supplementary Videos 3 and 4). Critically, no major off-target effects were observed, in terms of either peripheral T_reg_ cell expansion (Fig. 4h) or the population size and phenotype of non-T_reg_ cells in the brain (Supplementary Fig. 11). PHP.*GFAP*-IL-2 treatment was well tolerated, with no excess mortality through 300+ days of monitoring (*n* = 14). Both neuronal function (measured electrophysiologically) and astrocyte function (Ca^2+^ imaging) were unaffected by PHP.*GFAP*-IL-2 treatment (Extended Data Fig. 6), and no behavioral abnormalities were observed in treated mice (Extended Data Fig. 7). The blood–brain barrier remained histologically and functionally intact following gene delivery (Extended Data Fig. 8). A similar degree of T_reg_ cell expansion was observed with neuronal-derived IL-2, following treatment with PHP.*CamKII*-IL-2 treatment (Extended Data Fig. 9), demonstrating source-independent effects of IL-2 on T_reg_ cell expansion. Therefore, our dual-lock PHP.*GFAP*-IL-2 approach combines the key desired attributes for treatment of neuroinflammatory pathology: increased IL-2 production in the brain, a rapid and sustained effect, and a restricted locus of action to avoid generalized immune suppression.

### IL-2 delivery provides protection against neuroinflammation

**Fig. 5:**
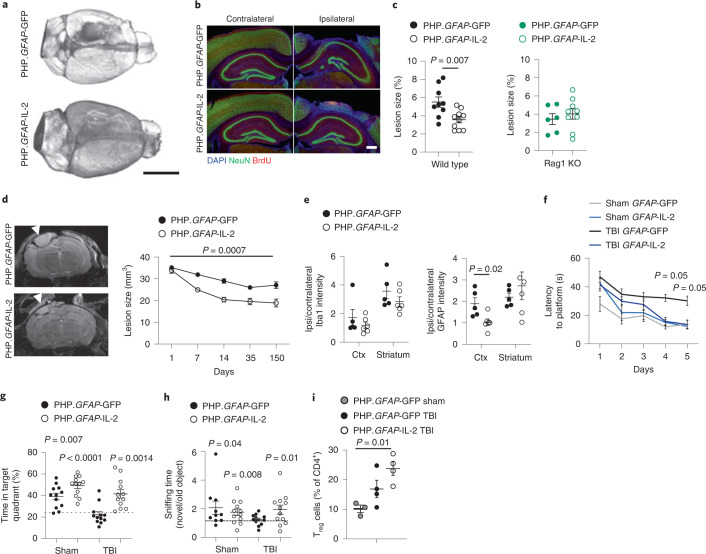
Gene delivery of IL-2 to the brain effectively prevents neurological damage during traumatic brain injury. **a**, Wild-type mice treated with PHP.*GFAP*-IL-2 (day -14) or PHP.*GFAP*-GFP control were given controlled cortical impacts to induce moderate TBI and examined at 14 d after TBI (*n* = 5, 6). Representative tomography of the surface of the brain. Scale bar, 0.5 cm. **b**, Representative immunofluorescence staining of the cortical tissue 14 d after TBI (*n* = 5, 6). NeuN (neurons), BrdU (proliferation), DAPI (nuclei). Scale bar, 50 µm. **c**, Quantification of lost cortical area 14 d after TBI in wild-type mice (left), treated with PHP.*GFAP*-IL-2 or control vector on day -14 (*n* = 9, 10), or *Rag1*-knockout (KO) mice (right), treated with PHP.*GFAP*-IL-2 or control vector (*n* = 6, 9). **d**, Representative MRI and MRI-based quantification of lesion size in PHP.*GFAP*-GFP or PHP.*GFAP*-IL-2-treated mice on days 1, 7, 14, 35 and 150 after TBI (control *n* = 16, 16, 12, 11, 10; IL-2 *n* = 16, 16, 16, 12, 9). Arrowhead indicates the impact site. **e**, Relative Iba1 and GFAP expression levels in the cortex and striatum (ratio of expression in ipsilateral versus contralateral hemispheres; *n* = 5, 6). **f**, Latency to find a hidden platform in the Morris water maze test during acquisition learning, for PHP.*GFAP*-GFP and PHP.*GFAP*-IL-2-treated mice, with and without (sham) TBI. *P* values are for TBI PHP.*GFAP*-GFP against TBI PHP.*GFAP*-IL-2 (*n* = 12, 12). **g**, Percentage of total time spent in the target quadrant during the probe trial (*n* = 12, 12). **h**, Ratio of time spent exploring a novel object over an old object during day 2 of the novel object recognition paradigm (*n* = 10, 12, 12, 12). **i**, Mice treated with PHP.*GFAP*-GFP control or PHP.*GFAP*-IL-2 (day -14) were given controlled cortical impacts and examined at 15 d after TBI (*n* = 3, 4, 4); a sham TBI was included in the PHP.*GFAP*-GFP group. Brains from sham, TBI and PHP.*GFAP*-IL-2-treated TBI mice were compared by flow cytometry for the frequency of T_reg_ cells as a proportion of CD4^+^ T cells. Data from **c**–**i** are shown as the mean ± s.e.m. All experiments were repeated independently (≥ twice). Statistical analyses were performed using non-parametric Mann–Whitney *U* test (**c** and **e**), one-way ANOVA (**i**) against chance level (**g** and **h**) or two-way ANOVA (**c**, **f** and **d**). Source data

To test the therapeutic potential of the dual-lock PHP.*GFAP*-IL-2, we treated mice with a control PHP.B (encoding GFP) or PHP.*GFAP*-IL-2 and then exposed them to TBI. The strong protective effect was apparent at a gross morphological level (Fig. 5a), with reduced loss of cortical tissue at 14 d after injury, as shown by histology (Fig. 5b,c) and magnetic resonance imaging (MRI; Fig. 5d). Astrogliosis was significantly reduced in the damaged cortex of PHP.*GFAP*-IL-2-treated mice (Fig. 5e). The neuroprotective effect was also observed at the behavioral level, where the poor performance of post-TBI mice in the Morris water maze and novel object recognition behavioral tests was completely reversed in PHP.*GFAP*-IL-2-treated mice (Fig. 5f–h). These results validate the neuroprotective potential of synthetic IL-2 delivery.

**Fig. 6:**
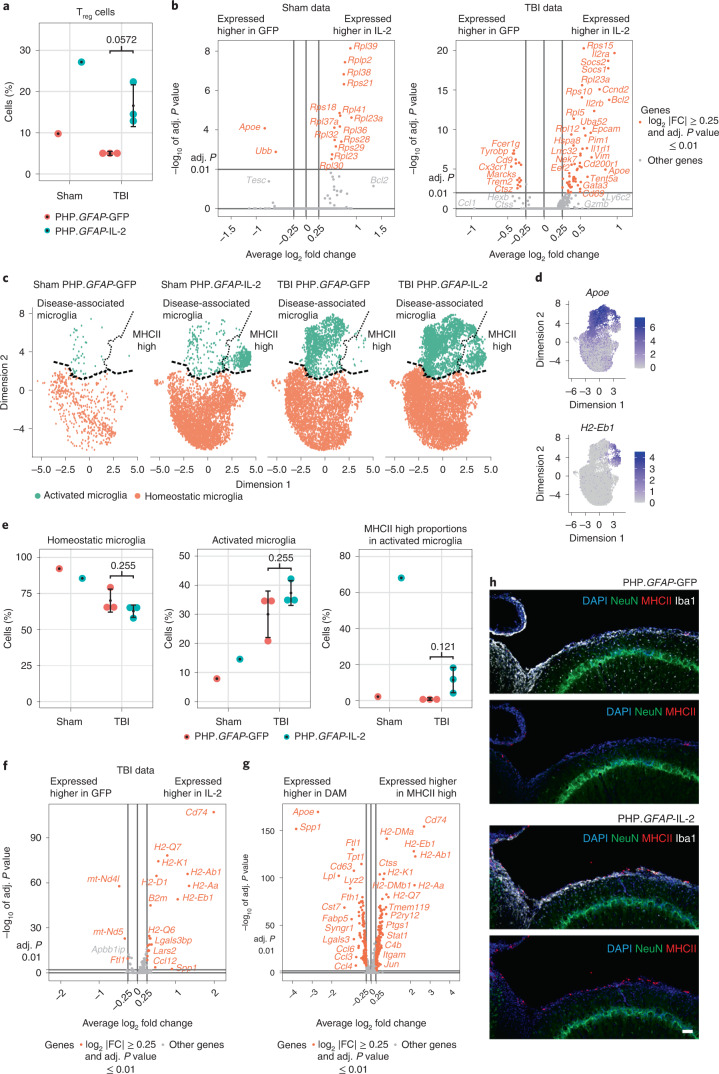
Brain-specific delivery of IL-2 drives microglial transcriptional divergence during TBI. **a**–**h**, Wild-type mice, treated with PHP.*GFAP*-IL-2 (or PHP.*GFAP*-GFP control vector) on day -14 were subjected to controlled cortical impacts to induce moderate TBI or sham surgery. At 14 d after TBI, T cells and microglia were sorted from the ipsilateral hemisphere of the perfused brains for 10x single-cell transcriptomics. **a**, T cells were clustered and annotated, based on markers defined in Extended Data Fig. 10a,b. Quantification of the T_reg_ cell cluster based on group. **b**, Volcano plot showing differential gene expression in the T_reg_ cell cluster between PHP.*GFAP*-GFP-treated mice and PHP.*GFAP*-IL-2-treated mice, for sham and TBI groups. **c**, Microglia uniform manifold approximation and projection (UMAP) representation, showing the location of cells per cluster for each treatment group. **d**, Cluster annotation based on expression of *Apoe* and *H2-Eb1*; expression of additional inflammatory markers is shown in Extended Data Fig. 10e. **e**, Quantification of the homeostatic and activated microglial clusters, and, within the activated microglial cluster, the relative contribution of the DAM and MHCII^hi^ subclusters. **f**, Volcano plot showing differential gene expression in the activated microglial cluster between PHP.*GFAP*-GFP-treated mice and PHP.*GFAP*-IL-2-treated mice, for TBI. **g**, Volcano plot showing differential gene expression, independent of treatment group, for the DAM versus MHCII^hi^ subclusters. **h**, Representative immunofluorescence staining of the cortical tissue at 14 d after TBI (*n* = 5, 6). NeuN, MHCII, DAPI and Iba1. Scale bar, 50 µm. Data from **a** and **e** are shown as the mean ± s.d.; *n* = 3 per group for TBI and *n* = 1 per group for sham. Statistical analyses were performed using unpaired two-tailed Student’s *t*-test (**a** and **e**) and volcano plots used the negative binomial test for differential expression (**b**, **f** and **g**). Source data

**Extended Data Fig. 10:**
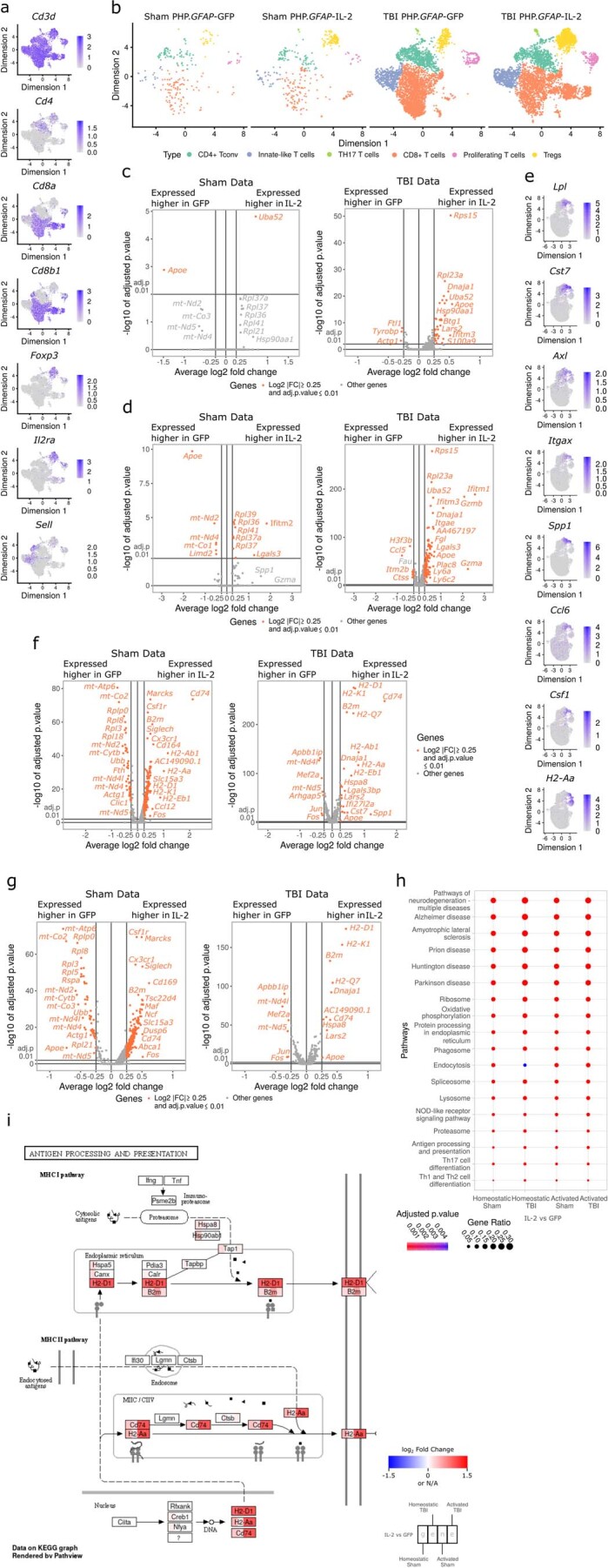
Transcriptional analysis following brain-specific delivery of IL-2 during TBI. Wildtype mice, treated with PHP.*GFAP*-IL-2 (or PHP.*GFAP*-GFP control vector) on day -14 were given controlled cortical impacts to induce moderate TBI or sham surgery. 14 days post-TBI, T cells and microglia were sorted from the perfused brains for 10x single-cell transcriptomics. **a**, UMAP expression plot of T cell data, with expression patterns of *CD3d, CD4, CD8, Foxp3, IL-2RA* and *Sel1* superimposed to identify various T cell populations. **b**, T cell UMAP representation, showing the relative numbers of various T cell types across treatment groups. **c**, Volcano plot showing differential gene expression in the CD4 Tconv cluster between PHP.*GFAP*-GFP and PHP.*GFAP*-IL-2-treated mice, for sham (left) and TBI (right). **d**, Volcano plot showing differential gene expression in the CD8 T cell cluster between PHP.*GFAP*-GFP and PHP.*GFAP*-IL-2-treated mice, for sham (left) and TBI (right). **e**, UMAP expression plot of microglia data, with differential expression patterns of *Lpl, Cst7, Axl, Itgax, Spp1, Ccl6, Csf1* and *H2-Aa* shown. **f**, Volcano plot showing differential gene expression for total microglia between PHP.*GFAP*-GFP and PHP.*GFAP*-IL-2-treated mice, for sham (left) and TBI (right). **g**, Volcano plot showing differential gene expression for homeostatic microglia between PHP.*GFAP*-GFP and PHP.*GFAP*-IL-2-treated mice, for sham (left) and TBI (right). **h**, Filtered overview of KEGG pathways based on differential gene enrichment in microglia from PHP.*GFAP*-GFP and PHP.*GFAP*-IL-2-treated mice, for sham and TBI conditions. **i**, Pathview plot for ‘Antigen processing and presentation’ (KEGG mmu04612), using the average log-fold changes between the four differential gene expression comparisons indicated. n = 3/group for TBI and n = 1/group for sham. Statistical analyses were performed using negative binomial test for differential expression, using the standard analysis pipeline in Seurat (f, g).

To test the mechanism of action, we first performed TBI and PHP.*GFAP*-IL-2 treatment on *Rag1*-knockout mice, deficient in adaptive immunity. Compared to wild-type mice subjected to TBI, lesions in *Rag1*-knockout mice following TBI were generally smaller (Fig. 5c), consistent with a partial role for adaptive immunity in TBI pathology. When given PHP.*GFAP*-IL-2, *Rag1*-knockout mice did not exhibit any beneficial effect from the brain-targeted IL-2 expression (Fig. 5c). These results formally exclude mechanisms of IL-2 action based on direct effects on the neuronal or glial compartments. The effect was, however, likely mediated through modification of the local environment, with little change observed to the inflammatory influx (Supplementary Fig. 12), other than the increase in brain T_reg_ cells (Fig. 5i) with enhanced amphiregulin production (Supplementary Fig. 12). Treatment prevented microgliosis formation, with the increase in microglia following TBI abrogated in PHP.*GFAP*-IL-2-treated mice (Supplementary Fig. 12a). We therefore performed single-cell transcriptomics analysis of T cells and microglia from treated and control mice, given TBI or sham surgery. Within the identified T cell clusters (Extended Data Fig. 10), the only population with shifts in frequency was T_reg_ cells, with increases in PHP.*GFAP*-IL-2-treated mice, both in sham and TBI animals (Fig. 6a). The transcriptome of expanded T_reg_ cells was largely conserved, with increases in IL-2 receptor components and the anti-apoptotic gene *Bcl2* (Fig. 6b), suggesting efficacy via numerical increase rather than the upregulation of unique effector molecules. Microglia were clustered into two superclusters, one representing homeostatic microglia and one representing activated microglia (Fig. 6c,d and Extended Data Fig. 10). While activated microglia were sharply elevated following TBI, the proportion of microglia in activated states was equivalent in IL-2-treated and control mice (Fig. 6e). Upregulation of genes encoding MHCII in IL-2-treated activated microglia was the prominent transcriptional change observed (Fig. 6f and Extended Data Fig. 10). Notably, MHCII^hi^ microglia from IL-2-treated mice formed a distinct subcluster within the activated microglia cluster (Fig. 6c,d). While the main activated subcluster expressed the classical disease-associated microglia (DAM) transcriptional profile^22^, the MHCII^hi^ subcluster was a low expressor of inflammatory mediators (Fig. 6g and Extended Data Fig. 10). IL-2 treatment was associated with a skewing of activated microglia away from the classical DAM phenotype and toward the atypical MHCII^hi^ phenotype (Fig. 6e). As MHCII^hi^ microglia accumulated at the lesion border (Fig. 6h), this unique population may serve as a buffer against neurotoxic inflammation.

**Fig. 7:**
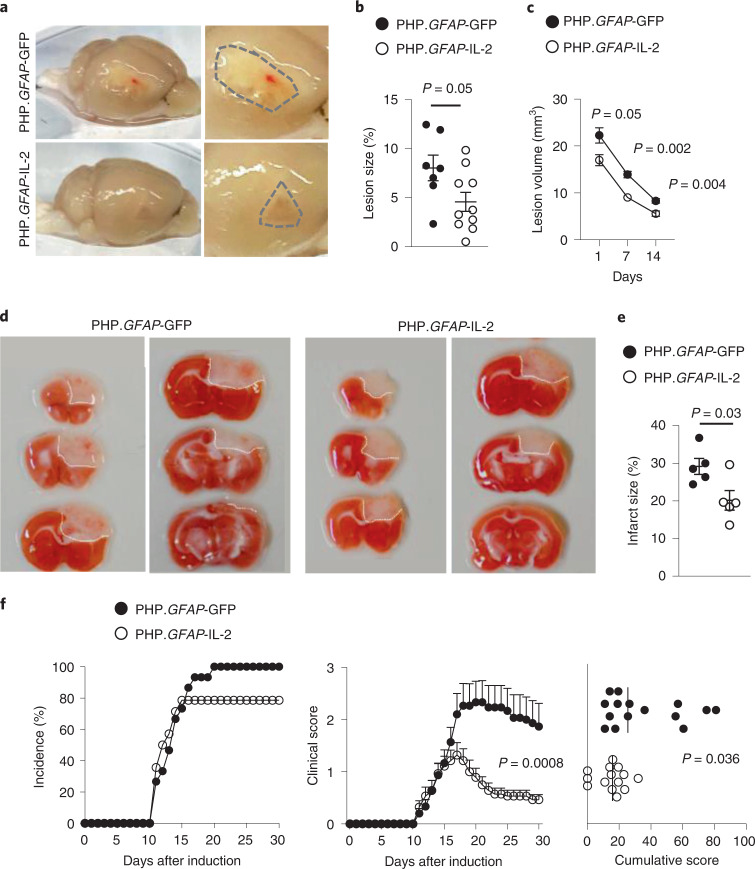
Neuroprotective utility for dual-lock IL-2 gene delivery across multiple neuroinflammatory pathologies. **a**, Wild-type mice, treated with control PHP.*GFAP*-GFP or PHP.*GFAP*-IL-2 on day -14, were given a dMCAO stroke and examined 15 d after stroke for macroscopic damage (outlined by dashed lines) with 2,3,5-triphenyl tetrazolium chloride (TTC)-aided quantification of stroke damage (*n* = 7, 10; **b**) and longitudinal MRI-based quantification of lesion size (*n* = 11, 17; **c**). **d**,**e**, Wild-type mice, treated with control PHP.*GFAP*-GFP or PHP.*GFAP*-IL-2 on day -14 (*n* = 5, 5), were given a photothrombotic stroke and examined 1 d after stroke for macroscopic damage (representative images, with lesion outlined; **d**) and TTC-aided quantification of stroke damage (**e**). **f**, EAE was induced in wild-type mice, following treatment with control vector (PHP.*GFAP*-GFP) or PHP.*GFAP*-IL-2 on day -14 (*n* = 15, 14). Incidence, daily clinical score (mean ± s.e.m.) and cumulative mean clinical score. All experiments were repeated independently (≥ twice). Statistical analyses were performed using unpaired two-tailed Student’s *t*-test (**b** and **e**), unpaired, non-parametric Mann–Whitney *U* test (**f**), or two-way ANOVA (**f** and **c**). Source data

To test the versatility of the dual-lock system, we extended these findings to other neuroinflammatory pathologies. We tested mouse models of ischemic stroke. Mice were pretreated with PHP.*GFAP*-IL-2 before the induction of a distal middle cerebral artery occlusion (dMCAO). Compared to control mice, PHP.*GFAP*-IL-2-treated mice developed a macroscopically smaller lesion (Fig. 7a), with a ~50% reduction in the histological lesion size at day 14 (Fig. 7b) and reduced lesion sizes detected by MRI from day 1 to 14 after induction (Fig. 7c). In the photothrombotic stroke model, mice pretreated with PHP.*GFAP*-IL-2 exhibited reduced macroscopic damage (Fig. 7d), and a ~30% reduction in infarct size as quantified by combined scar tissue and ischemic tissue identification (Fig. 7e). In both the dMCAO (Supplementary Fig. 13) and photothrombotic (Supplementary Fig. 14) models, analysis of the immunological compartment indicated elevated numbers of T cells (CD4^+^, CD8^+^ and T_reg_ cells) in the brain following injury, with no notable additional effect of treatment. We then turned to the EAE model of MS. Using pretreatment of mice, PHP.*GFAP*-IL-2 resulted in a lower incidence, reduced clinical time course and a lower cumulative clinical score in the MOG model (Fig. 7f). As with the stroke models, the immunological composition of the brain was largely unchanged at the time points assessed, including the number of T_reg_ cells present, although elevated production of anti-inflammatory cytokines was observed (Supplementary Fig. 15). The equilibration of brain-resident T_reg_ cells in control and treated mice, 4+ weeks after initial PHP.*GFAP*-IL-2 treatment, across both stroke and EAE, suggests either a maximal duration of efficacy following a single AAV dose, or a confounding effect of pathology-derived T_reg_ cells obscuring the treatment-derived T_reg_ cell increase. Further, the observed efficacy in these models, despite T_reg_ cell normalization at end stage, suggests that either the major effects were implemented during earlier phases of disease, or immune modulation of a local population, such as microglia, extends beyond the period of elevated T_reg_ cell numbers.

**Fig. 8:**
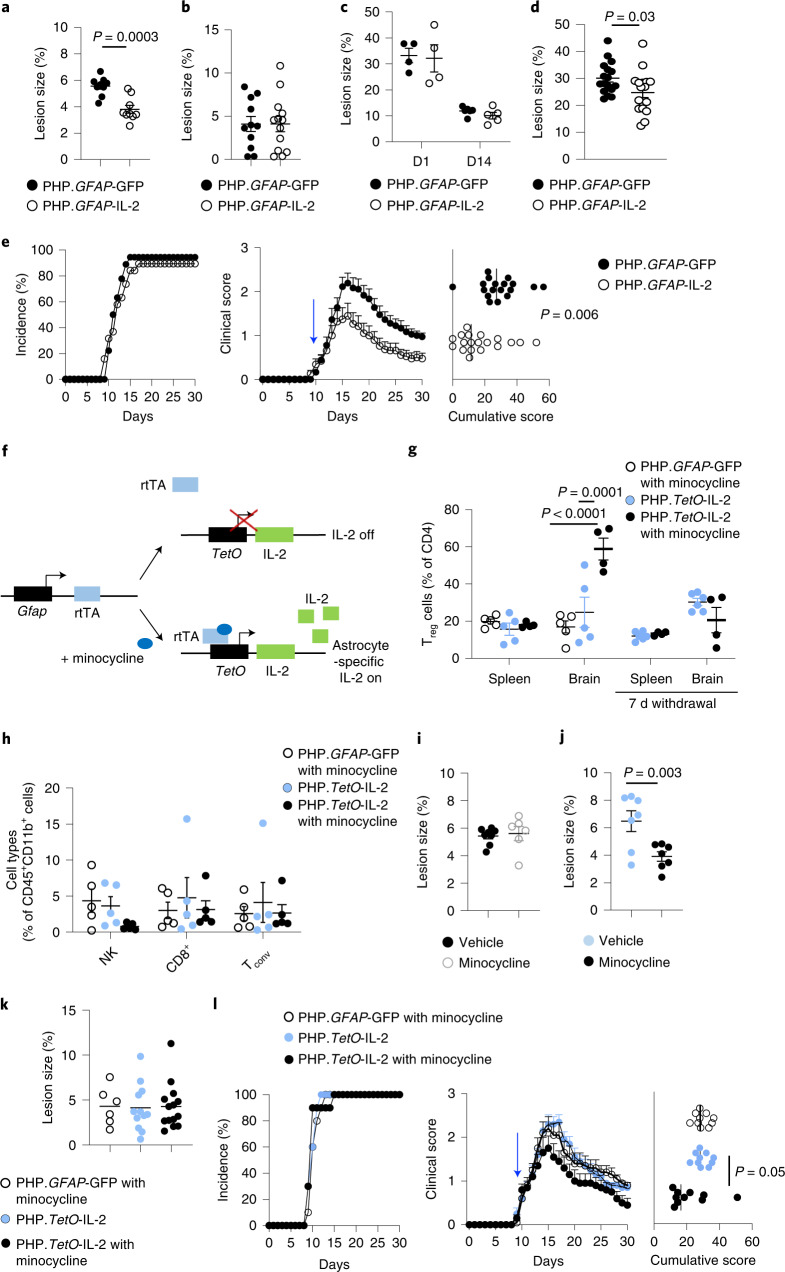
Neuroprotective utility for dual-lock IL-2 gene delivery across multiple neuroinflammatory pathologies. **a**, Mice were given TBI, followed by treatment with PHP.*GFAP*-IL-2 or PHP.*GFAP*-GFP, examined 14 d after TBI (*n* = 9, 9). Quantification of lost cortical area. **b**, Mice were given dMCAO stroke and treated with PHP.*GFAP*-GFP or PHP.*GFAP*-IL-2, and examined at 15 d after stroke for TTC-aided quantification (*n* = 11, 13). **c**, Mice were given photothrombotic stroke and treated with PHP.*GFAP*-GFP or PHP.*GFAP*-IL-2, and examined 1 d (*n* = 4, 4) or 14 d (*n* = 6,5) after stroke for TTC-aided quantification. **d**, TTC-aided quantification of post-secondary stroke (*n* = 17, 17). **e**, EAE was induced in wild-type mice and, 10 d after induction, mice were treated with PHP.*GFAP*-GFP or PHP.*GFAP*-IL-2 (*n* = 18, 19; blue arrow): incidence, daily clinical score, cumulative mean clinical score (*n* = 15, 14). **f**, Design of PHP.*TetO*-IL-2.*GFAP*-rtTA. **g**, Wild-type mice were administered control vector or PHP.TetO-IL-2.*GFAP*-rtTA and gavaged with PBS or minocycline. The number of brain T_reg_ cells was assessed 11 d after treatment (*n* = 5, 5, 4). Additional groups were assessed 1 week after minocycline withdrawal (*n* = 6, 4). **h**, Frequency of CD8^+^ T cells, natural killer (NK) cells and CD4^+^ T_conv_ cells in the brain (*n* = 5 per group). **i**, Wild-type mice were given TBI, followed by control vector. Quantification of cortical area lost on day 14 (*n* = 8, 6). **j**, Wild-type mice were given TBI, followed by treatment with PHP.*TetO*-IL-2.*GFAP*-rtTA, with or without minocycline. Quantification of lost cortical area on day 14 (*n* = 7, 7). **k**, Mice were given dMCAO stroke and treated with control vector plus minocycline, or PHP.*TetO*-IL-2.*GFAP*-rtTA, without or with minocycline. Mice were examined at 15 d after stroke for TTC-aided quantification (*n* = 6, 12, 14). **l**, EAE was induced in wild-type mice, following treatment with PHP.*TetO*-GFP.*GFAP*-rtTA or PHP.*TetO*-IL-2.*GFAP*-rtTA, with or without minocycline on day 10 (*n* = 10 per group) after induction. Incidence, daily clinical score and cumulative mean clinical score are shown. Data are the mean ± s.e.m. All experiments were repeated independently (≥ twice). Statistical analyses were performed using unpaired two-tailed Student’s *t*-test (**a**, **d** and **j**), unpaired, non-parametric Mann–Whitney *U* test (**e** and **i**) or two-way ANOVA with Tukey’s test (**g**). Source data

To investigate the translational potential of this approach, we tested PHP.*GFAP*-IL-2 treatment in the curative context. First, we used the controlled cortical impact model, where pretreatment with PHP.*GFAP*-IL-2 reduced the size of the developing lesion (Fig. 5b,c). Taking a curative approach, we first subjected mice to TBI and then treated the animals after injury with PHP.*GFAP*-IL-2. As with the preventative treatment, the curative approach reduced the size of the developing lesion compared to that observed in control-treated mice (Fig. 8a). In stroke, however, curative PHP.*GFAP*-IL-2 treatment after stroke induction did not reduce the resulting lesion size in either the dMCAO (Fig. 8b) or photothrombotic (Fig. 8c) models. This suggests that unfavorable kinetics of IL-2 production preclude efficacy during the rapid damage that occurs following stroke. We therefore developed a model of secondary stroke, where photothrombotic stroke was induced in one hemisphere, mice were treated with PHP.*GFAP*-IL-2 or control, and then 14 d later photothrombotic stroke was induced in the opposite hemisphere. In this context, treatment with IL-2 following the primary stroke resulted in significant reduction of lesion size in the secondary stroke (Fig. 8d). For MS, we again used the EAE model; however, we waited until the mice developed clinical manifestations and then treated with control PHP.B or PHP.*GFAP*-IL-2. Strikingly, the protective effect of PHP.*GFAP*-IL-2 was still observed, with separation of the clinical time course by day 15 and a sharp reduction in the cumulative clinical score (Fig. 8e).

The development of a brain-specific IL-2 delivery system provides the potential for use in clinical neuroinflammation contexts. Translation to the clinic requires, however, the ability for dose modification and withdrawal capacity. To add these clinically desirable features, we included a third layer of control through a ‘Tet-On’ system. In this system, IL-2 expression was shifted under the control of a TetO-dependent promoter, with the rtTA activator controlled by the *GFAP* promoter (Fig. 8f). A modified rtTA was used to allow response to the blood–brain barrier-permeable drug minocycline^23^. This ‘triple-lock’ AAV provided the same brain-specific expansion of T_reg_ cells as the dual-lock system, but only in the presence of minocycline (Fig. 8g). Other major leukocyte populations present in the brain were unaffected in frequency (Fig. 8h). Following the withdrawal of minocycline, brain T_reg_ cell numbers returned to baseline levels within 1 week (Fig. 8g). To validate this triple-lock system in the disease context, we first used TBI. Minocycline by itself did not alter the lesion size (Fig. 8i); however, the combination of the triple-lock and minocycline treatment, given after injury, substantially reduced the size of the resulting lesion (Fig. 8j). In stroke, by contrast, the treatment did not alter lesion size, supporting an incompatibility with the kinetics of pathology (Fig. 8k). Finally, in EAE, the combination of the triple-lock system and minocycline treatment, given after disease symptoms developed, resulted in earlier plateau of symptoms and lower cumulative pathology (Fig. 8l). These results validate the triple-lock gene-delivery system for brain-restricted IL-2 expression as having both of the key clinical requirements of efficacy and dose control.

## Discussion

Here we demonstrated the utility of brain-specific IL-2 as a neuroprotective agent and developed a delivery platform suitable for clinical application. Gene delivery resulted in increased brain IL-2 concentration followed by a lagging increase in resident T_reg_ cell frequency, consistent with the effect on clinical progression following treatment in the EAE context. In the context of stroke, where current treatments sharply lose efficacy beyond 3 h after stroke^24^, the delay in IL-2 could account for the disparity between the protective pre-injury regime and the ineffective post-injury treatment. While both direct and indirect mechanisms for IL-2-mediated protection are plausible, the most parsimonious explanation remains the capacity of IL-2 to expand the local T_reg_ cell population. The brain provides a relatively IL-2-deficient environment for T_reg_ cells, a state known to induce apoptosis and limit population size^13^. The dependency of IL-2 treatment on the presence of the adaptive immune system, as demonstrated through Rag-deficient mice given TBI and PHP.*GFAP*-IL-2, is consistent with T_reg_ cells being the key mediator of the neuroprotective effect of brain-delivered IL-2. Despite this, we do not discount the possibility that neurogenic IL-2 works, at least in part, through effects on other cell types. While an alternative mechanism is not identified, it is important to note that there is not a strict concordance between brain T_reg_ cell frequency and treatment efficacy. In each neuroinflammatory model, the pathology-induced immune changes outweigh the treatment-induced immune changes at the assessed time points. However, it is also important to consider that pathological modification and impact on clinical progression are not necessarily temporally coupled. Thus, a transient pulse of brain T_reg_ cell expansion early on during disease or injury may drive long-lasting improvements in pathology at time points where the treatment effect has been washed out.

Without discounting the possibility of non-canonical effects of IL-2, the expansion of brain-resident T_reg_ cells provides a functionally dynamic mediator for local immune modulation. T_reg_ cells are capable of producing multiple anti-inflammatory agents, many of which are used in clinical practice to treat neuroinflammation^25^. T_reg_ cells also possess key reparative functions, beyond their direct immunosuppressive role^7,26^. The use of IL-2 to expand the T_reg_ cell population bypasses the problem of identifying the ideal immunosuppressive mediator, and instead piggybacks on the adaptive properties of T_reg_ cells, which are capable of sensing and responding to local microenvironmental cues. The small number of brain-resident T_reg_ cells, however, even following local expansion, suggests a more common cell type is likely required as an effect amplifier. Microglia are an attractive candidate for this putative intermediatory, with their transcriptional profiles heavily modified by treatment. In particular, while activated microglia in control-treated TBI mice gained the classical inflammatory transcriptional profile, a substantial subset of activated microglia in IL-2-treated mice sharply upregulated MHCII expression, without additional inflammatory markers. This upregulation correlated with localization along the injury border, indicating a potential function as a buffer to the expanding zone of neurotoxicity. The association between MHCII upregulation and restraint in inflammatory marker expression is intriguing. Early upregulation of MHCII on microglia has previously been associated with enhanced protection and repair of the injured CNS^27,28^. MHCII expression indicates enhanced capacity for direct cognate interaction between microglia and T_reg_, and may allow increased local production of multiple anti-inflammatory mediators. Alternatively, *Cd74*, a chaperone for MHCII, is highly upregulated in our system and directly impedes inflammatory polarization of microglia^29^. Within the DAM compartment, IL-2 treatment also resulted in greater upregulation of anti-inflammatory *Spp1* (osteopontin)^30^, proposed as a therapeutic in TBI and other neuroinflammatory diseases^31^. We caution, however, against overly simplistic models of a single molecular mediator, with the multipotent functions of T_reg_ cells more compatible with complex synergistic effects.

Despite the biological potency of IL-2, incorporation into therapeutics has been slow. The short half-life of only 15 min necessitates either constant delivery or high doses, which in turn alters biological targets (including direct effects on the blood–brain barrier^32^). Proof-of-principle studies have demonstrated the capacity of IL-2 to delay neuroinflammatory or neurodegenerative disease^17–19^, including via AAV-mediated systemic delivery of IL-2 (ref. ^16^). However global immunosuppression is not a viable strategy to combat neuroinflammatory disease, especially in patients with enhanced susceptibility to infections^33^. Local IL-2 production provides an alternative approach, bypassing the unpalatable consequences of systemic IL-2 delivery.

Here we used αCamKII^+^ neurons as the source of local IL-2 production in the proof-of-concept phase, based on the observation that neurons are the primary source of IL-2 in the healthy brain^34^. In a therapeutic setting, however, astrocytes potentially have superior properties as a delivery source, facilitated by their highly efficient secretory system^35^. Here, we demonstrate that this system can be effectively ‘hijacked’ and exploited for local IL-2 production and secretion, with the additional advantage that astrocytic endfeet are in close proximity to the vasculature zones where brain-resident T cells are concentrated^5^. The involvement of astrocytes in the pathophysiology of neurological injury and disease potentially acts as a biological amplification process. Reactive gliosis is poorly understood at the molecular level but, when unresolved, appears largely deleterious, being concomitant to neuronal loss^36^. However, for our purposes, the upregulation of the *GFAP* promoter and astrogliosis served to concentrate IL-2 production near to regions of reactive astrogliosis. The system, therefore, contains the features of a natural ‘rheostat’, using the molecular signature of neurological damage to both amplify and anatomically direct the therapeutic response.

Gene-delivery systems, such as the triple-lock IL-2 system developed and validated here, have high potential for translation. While early setbacks delayed the clinical uptake of AAV-based systems, improved vectors with superior safety profiles are gaining regulatory approval^37^, including Zolgensma (onasemnogene abeparvovec; intravenous) for spinal muscular atrophy, with other CNS diseases under intensive investigation^38^. The ability of AAV-based vectors to transduce both dividing and nondividing cells, combined with low inherent immunogenicity, avoids many of the limitations of alternative vector types, such as adenovirus-based and lentivirus-based systems. Furthermore, as AAV-based vectors provide strong and sustained transgene expression (over 4 years in humans^39^ and 15 years in nonhuman primates^40^), use of such a system should ensure long-term therapeutic benefits—an attractive proposition in progressing or relapsing diseases such as MS, or injuries with a chronic component, such as TBI. Even in stroke, with a narrow treatment window, a lasting gene delivery of IL-2 may have clinical benefit; 10% of stroke patients experience a secondary stroke within 90 d^41^. While PHP.B was used here for its superior transduction of the murine CNS following intravenous injection^20^, poorer results have been observed in non-human primates^42^ and are expected, by extension, in humans. Both direct injections or intrathecal delivery can potentially overcome this problem, but remain highly invasive procedures. The adoption of alternative AAV capsids, showing more efficient CNS penetration in humans following systemic delivery, would retain the clinically non-invasive nature and potentially avoid preexisting immunity^43^ and potential off-target toxicity. As the delivery system developed here relies on a modified version of the endogenous promoter, rather than capsid, for lineage specification, it can be readily adapted to alternative AAV capsids for human use. Additional refinements may arise from the recent findings of molecular heterogeneity among astrocytes^44^, providing promoter elements allowing specific microanatomical targeting of therapeutic delivery. The coupling to small-molecule inducers, such as validated here, provides both a dose-escalation function and a safety-withdrawal capacity. Minocycline-based systems combine the appropriate biodistribution with the intriguing potential for synergy, as minocycline itself is a mild neuroprotective agent, with potential efficacy in TBI^45^. The lack of viable alternatives to treat the neuroinflammatory component of CNS pathologies warrants the further investigation of the triple-lock IL-2 delivery system for clinical development.

## Methods

### Mice

*Foxp3*-Cre transgenic mice^46^, *αCamKII*-CreERT2 transgenic mice^47^, *Plp1*-CreERT transgenic mice^48^, *Il-2*-GFP mice^49^ and *Rag1*-knockout mice^50^ were used on the C57BL/6 background. Rosa-IL-2 mice were generated through the insertion of a cassette containing a floxed-STOP sequence followed by an *Il-*2-IRES-*Gfp* sequence into the Rosa26 locus, using the endogenous *Rosa26* promoter^51^, and were used on the C57BL/6 background. Mice were housed under specific pathogen-free conditions, under a 12-h light/dark cycle in a temperature- and humidity-controlled room with *ad libitum* access to food and water. All animal procedures were approved by the KU Leuven Animal Ethics Committee (P035/2015, P015/2014, P209/2015, P043/2016, P082/2018, P124/2019), the University of Amsterdam (CCD 4925, AVD1110020184925) or the Babraham Institute Animal Welfare and Ethics Review Body (PP3981824) taking into account relevant national and European guidelines. Both male and female mice (8–12 weeks old) were used in this study, unless otherwise specified. Age and sex of study mice and treatment regime were selected in consultation with the Animal Ethics Committees. Tamoxifen (Sigma, T5648) was solubilized in corn oil (Sigma) at 10 mg ml^−1^. Five- to seven-week-old mice were injected three times, intraperitoneally, at 48-h intervals using a dose of 100 mg per kilogram body weight. Minocycline (PBS vehicle) was administrated at 50 mg per kg body weight, through daily oral gavage. Sample sizes for mouse experiments were chosen based on power calculations and pilot data, in conjunction with the Animal Ethics Committee, to allow for robust sensitivity without excessive animal use. Mice were selected randomly for inclusion into the various experimental groups, with the animal technicians performing experimental procedures and clinical measurements blinded as to the identity of experimental groups. For behavioral methods, see the Supplementary Information.

### Parabiosis

For parabiosis, pairs of 7- to 10-week-old female mice were co-housed for 14–21 d before surgery. C57BL/6.SJL-*Ptprc*^*a*^/BoyJ mice (CD45.1) were parabiosed to αCamKII^IL-2^ mice (CD45.2), pretreated with tamoxifen, for 10 weeks. Pairs of mice were anesthetized with inhaled isoflurane, 3.5% vol/vol induction and 2.5–3.0% vol/vol maintenance. Carprofen and buprenorphine were delivered intraperitoneally at a dose of 10 mg per kg body weight and 0.1 mg per kg body weight, respectively, before surgery. Fur was removed from the surgical site. Mice were laid supine and the surgical site was disinfected with betadine solution, followed by 70% ethanol. Longitudinal skin incisions were made to the shaved sides of each animal, starting at 0.5 cm above the elbow and extending all the way to 0.5 cm below the knee joint. The skin was gently detached from the subcutaneous fascia to create 0.5 cm of free skin, and sutured together to generate a parabiotic pair.

### Traumatic brain injury

A moderate cortical TBI was induced using the controlled cortical impact model with minor adjustments. Male mice were treated with tamoxifen (6 weeks old) or vector (10 weeks old). At 12 weeks, mice were anesthetized using 5% isoflurane and placed in a stereotaxic frame. Mice were kept anesthetized using 2% isoflurane throughout the procedure. A craniotomy was performed creating a window over the left hemisphere, ranging from lambda to bregma. The impact piston (Leica Impact One) had a 3-mm metal tip and was placed on top of the left cortex at a 20° angle. An impact was performed using the following settings: 5.5 m s^−1^ velocity, 1 mm impact depth and 300 ms dwell time. Immediately after the impact, the skull bone was replaced and attached using superglue. The skin was stitched to close the wound and mice were allowed to recover on a heat pad until fully awake. The sham group underwent craniotomy but did not receive the impact. The TBI-induced perfusion deficit in the brain was measured by MRI at 24 h and on days 7–14 after impact. Flow cytometry profiling and immunohistochemistry analysis were performed 14 d after impact.

### Experimental autoimmune encephalomyelitis

EAE was induced in female mice, aged 8–12 weeks. To induce active EAE, mice were immunized with 50 μg of MOG_35–55_ peptide (Covalab) emulsified with Complete Freund Adjuvant containing 2 mg ml^−1^ of *Mycobacterium tuberculosis* (Sigma). Then, 200 ng ml^−1^ pertussis toxin (List Biochemicals) was given on day 0 and day 2 after immunization. Clinical score was evaluated blind by a technician on a scale from 0 to 5 (ref. ^52^).

### Photothrombotic stroke

Focal cortical ischemia was induced using the photothrombotic lesion model. Mice (male, 12 weeks old) were anesthetized with 2.5% isoflurane in an oxygen/air mixture, respiration was monitored and rectal temperature was maintained at 37 ± 0.5 °C with a heating plate (TCAT-2LV Controller, Physitemp Instruments). After fixation in a stereotaxic frame attached to a digital display (David Kopf Instruments), the skull was exposed by a 1-cm midline incision of the skin. Then, 100 µl rose bengal (Sigma) at a concentration of 3 mg ml^−1^ in saline, was injected via the tail vein. For illumination, a 2.4-mm laser beam with a wavelength of 565 nm (L4887-13, Hamamatsu Photonics) was focused on the motor cortex responsible for forepaw function (0.5 mm rostral, 1.8 mm lateral of bregma). At 5 s after rose bengal injection, the brain was illuminated through the intact skull for a total duration of 5 min. After illumination, the incision was sutured and animals were given 500 µl saline and 0.05 mg per kg body weight Vetergesic (Ecuphar) subcutaneously. During recovery, mice were placed in a separate cage with half of the cage placed under an ultraviolet lamp before returning to their home cage and housing facility. After stroke, mice were monitored on a daily basis during the first week; after that, animal health was checked weekly. In mice with a secondary stroke, treatment with AAV occurred intravenously after the primary stroke. Mice were then allowed to recover for 2 weeks before receiving a secondary stroke on the opposite hemisphere, using the same procedure. To prevent unnecessary suffering of animals, mice were euthanized if they showed severe weakness or lost 20% of their body weight within 5 d.

At 24 h after stroke, mice were anesthetized with an overdose of Dolethal (20 mg ml^−1^; Vetoquinol) and transcardially perfused with PBS. Brains were collected and cut in 1-mm sections using a mouse brain matrix (Zivic Instruments). For each animal, a total of six sections surrounding the infarct were collected and incubated in a 1% (wt/vol) TTC (Sigma-Aldrich) in PBS solution for 25 min at 20 °C, while protected from light. When stained, sections were put on glass plates and pictures were taken with a regular photo camera (Nikon). To correct for edema developed during the early phase of ischemia, stroke area was calculated according to the method provided by Swanson and colleagues^53^: [lesion area] = [area of the contralateral side] − [total undamaged area] and presented as a percentage of the contralateral hemisphere. The identification of the undamaged ipsilateral area involved mapping the zone surrounding the combined missing scar tissue and TTC-stained ischemic zones.

### Permanent distal middle artery occlusion

Ten-week-old male mice were subjected to permanent dMCAO, essentially as described^54^, but with minor modifications. Briefly, the mice were anesthetized with 2% (vol/vol) isoflurane and placed in a lateral position on a heat pad to maintain body temperature. Eye ointment (Duratears; Alcon) was applied to prevent dehydration of the eyes. The surgical site was shaved and disinfected with ethanol and a vertical skin incision was made between the left ear and eye. Next, a surgical window was opened in the skin and the temporal muscle was separated, dorsal and apical, with surgical scissors to expose the temporal bone without removing the muscle. The middle cerebral artery (MCA) was identified and a hole was burred with a microdrill (Stoelting) at the site of MCA bifurcation. The remaining bone and the overlying dura mater were subsequently removed with forceps. Permanent occlusion of the MCA was then performed with bipolar coagulation forceps (0.4-mm tip; ERBE) with the electrosurgical unit (ERBE ICC 50) set at 8 W. During surgery, the surgical site was kept hydrated using saline. After visual confirmation of MCA occlusion (reduced blood flow), the muscle was put back into place and the wound was sutured and disinfected, and the animals were allowed to recover in a preheated environment. Mice that developed subarachnoid hemorrhage during surgery were excluded from the study. Sham animals followed the same surgical procedure except for the final coagulation step.

### Magnetic resonance imaging

Mice with TBI were scanned 1 day, 1 week, 2 weeks, 4 weeks and 3 months after injury. Mice with dMCAO were scanned 1 day, 1 week and 2 weeks after injury. MRI measurements were performed on a 9.4T Bruker BioSpec small-animal MR system (20-cm horizontal bore; Bruker BioSpin), using a quadrature resonator with an inner diameter of 7.2 cm for transmission and an actively decoupled mouse brain surface coil for receiving (Rapid Biomedical). The scanner was equipped with an actively shielded gradient set of 600 mT m^−1^. Mice were scanned under isoflurane anesthesia (1–2% (vol/vol) isoflurane in 100% (vol/vol) O_2_ administered through a snout mask). Rectal temperature and respiratory rate were continuously monitored (SAII), and isoflurane levels were adjusted to maintain a respiratory rate of 80–100 breaths per minute. Rectal temperature was maintained at 37 °C (36–37.5 °C).

Following an initial localizer scan, the MRI protocol used an axial T_2_-weighted spin-echo sequence with a repetition time (TR) of 4.5 s, effective echo time (TE) of 40 ms, rare factor 8, 1 average, matrix 256 × 256, field of view (FOV) 20 × 20 mm and 24 slices of 500-µm thickness. A multi-slice-multi-echo sequence was acquired for the calculation of parametric T2 maps using the same slice orientation as for the T_2_-weighted MRI and the following parameters: TR of 4.0 s, 12 TE increments of 12 ms, 1 average, matrix 128 × 128, FOV 20 × 20 mm and 24 slices of 500-µm thickness. A diffusion-weighted MRI was acquired for the calculation of parametric apparent diffusion coefficients using the following parameters: TR of 2.0 s, TE of 20 ms, 1 average, matrix 128 × 128, FOV 20 × 20 mm, 20 slices of 500-µm thickness with a 200-µm gap in between slices, b-values of 0, 100, 300, 500, 800, 1,000 and 1,500. Finally, a three-dimensional (3D) gradient echo sequence (FLASH) with the following parameters was acquired: TR of 30 ms, TE of 7 ms, 20° pulse, matrix 160 × 160 × 96, FOV 20 × 20 × 12 mm, resulting in an isotropic resolution of 125 µm. Operators were masked to the experimental group. All MR images were processed using the Bruker BioSpin software Paravision 6.1. Parametric T_2_ and apparent diffusion coefficient maps were calculated in Paravision 6.1, through a pixel-wise mono-exponential fit. For quantification of lesion volumes, Paravision 6.1 software was used.

### AAV vector production and purification

AAV-PHP.B production was performed by Vigene Sciences or VectorBuilder, using the classical tri-transfection method, with subsequent vector titration performed using a quantitative PCR-based methodology^21,55^. For AAV-PHP.B.*GFAP*-IL-2 and PHP.B.*αCamKII*-IL-2, the mouse IL-2 coding sequence, together with 5′ and 3′ untranslated regions (accession no. BC116845) was cloned into a single-stranded AAV2-derived expression cassette, containing a 2.2-kb human *GFAP* promoter^56^ or full length murine *CamKII* promoter (gene ID: 12322), woodchuck hepatitis post-transcriptional regulatory element and bovine growth hormone polyadenylation sequence. Control vectors were prepared by swapping the IL-2 coding sequence for that encoding enhanced green fluorescent protein (EGFP, Vector Biolabs).

PHP.B.TetO:IL-2.sGFAP: rtTA(V7/V14) was constructed with a 7xTetO sequence and minimal CMV promoter driving IL-2, and a short human *GFAP* promoter driving an rtTA fusion protein modified to include the Val7/Val14 mutations for enhanced minocycline response.

In both cases, vector (100 µl total volume) was administered to mice intravenously at 1 × 10^9^ vector genomes per dose, unless otherwise specified. Batch concentration was normalized using brain T_reg_ cell expansion as a biological readout. Mice were used for experimental procedures at least 14 d after AAV injection, unless otherwise indicated.

### Flow cytometry

Mice were deeply anaesthetized with an intraperitoneal injection of a ketamine (87 mg per kg body weight) and xylazine (13 mg per kg body weight) mixture. Blood was collected from the right ventricle before transcardial perfusion with ice-cold PBS. Blood was prepared by red blood cell lysis; single-cell suspensions from lymphoid organs were prepared by mechanical dissociation; single-cell suspensions from brain tissue were prepared by digestion for 30 min at 37 °C with 1 mg ml^−1^ collagenase IV (Thermo Fisher), 300 µg ml^−1^ hyaluronidase (Sigma-Aldrich) and 40 μg ml^−1^ DNase I (Sigma-Aldrich) in RPMI 1640 supplemented with 2 mM MgCl_2_, 2 mM CaCl_2_ 20% FBS and 2 mM HEPES pH 7.4 (Gibco), followed by mechanical disruption, filtration (through 100 µm mesh) and enrichment for leukocytes by gradient centrifugation (40% Percoll GE Healthcare, 600*g*, 10 min, no brake). For estimation of absolute cell numbers, counting beads were ‘spiked in’ at the initial step, allowing for calculation of cell loss during preparation. Non-specific binding was blocked using 2.4G2 supernatant. To assess intracellular cytokine production, cells were cultured for 4 h in the presence of phorbol myristate acetate (1 µg ml^−1^; Sigma-Aldrich), ionomycin (1 µg ml^−1^; Sigma-Aldrich) and brefeldin A (2 µg ml^−1^; BD). Cells were fixed and permeabilized with the eBioscience Foxp3 staining kit (eBioscience). Cellular phenotypes were assessed using high-parameter flow cytometry panels, containing markers to identify cell types and markers to assess activation states. Data were acquired on a BD FACSymphony, with panels covering (1) CD45, CD4, CD8α, CD3, CD19, NK1.1, Foxp3, eBioscience Fixable Viability Dye eFluor 780, CD103, CD62L, GITR, CD25, neuropilin-1, ST2, PD-1, CTLA4, KLRG1, Helios, CD69, ICOS, CD44, T-bet, TCRγδ and Ki67; or (2) CCR6, CD80, TCRγδ, CD45, Foxp3, MHCII, eBioscience Fixable Viability Dye eFluor 780, pro-IL-1β, CD25, Ly6G, ST2, CX3CR1, PD-L1, TNF, CD44, Ki67, CD4, Ly6C, TrkB, CD19, CD69, CD8α, LAMP1, CD64, CD11b, CD3, TGF-β and streptavidin; or (3) Foxp3, eBioscience Fixable Viability Dye eFluor 780, IL-17, CD4, IFN-γ, CD8α, TNF, CD3, amphiregulin, IL-10, CD11b, CD19, granulocyte macrophage-colony stimulating factor (GM-CSF), TCRγδ, pro-IL-1β, TCRβ, IL-2 and NK1.1. For brain panels, the entire brain sample was acquired. Data were compensated using AutoSpill^57^. Examples for mouse cells were always presented as concatenated biological replicates. Cell sorting was performed using a BD FACSAria III with a panel including CD4, CD11b, CD45, TCRβ, eBioscience Fixable Viability Dye eFluor 780 and CD25. Flow cytometry data collection was performed using FACSDiva version 8.0.2 (BD) or SpectroFlo (Cytek). Flow sorting was performed using FACSDiva version 8.0.1 (BD). Representative gating for brain T_reg_ cell quantification is shown in Supplementary Fig. 16.

### Fluorescence immunostaining

Mice were deeply anaesthetized with intraperitoneal injection of a ketamine (87 mg per kg body weight) and xylazine (13 mg per kg body weight) mixture and transcardially perfused with PBS followed by 4% buffered formalin solution. The brain was removed and fixed in 10% buffered formalin solution overnight and stored in 30% sucrose until preservation in tissue-freezing medium (Shandon Cryomatrix embedding resin, Thermo Scientific), and stored at −80 °C. Sections (20–50 µm) were washed for 15 min in 50 mM NH_4_Cl-PBS and pre-blocked with 10% normal donkey serum in 0.5% Triton X-100-PBS for 1 h at 20 °C. Aldh1l1 immunofluorescence required heat-induced epitope retrieval at 80 °C for 30 min in a 10 mM sodium citrate buffer (pH 6.0). Sections were incubated overnight at 4 °C with primary antibodies directed against Foxp3 (1:500 dilution; MAB8214, R&D systems), CD4 (1:250 dilution, 100506, BioLegend), Iba1 (1:1,000 dilution; 014-19741, Wako), GFAP (1:500 dilution; ab4674, Abcam), CD31 (1:100 dilution; MA3105, Invitrogen), S100ß (1:1,000 dilution; S2532, Sigma-Aldrich), APC (1:250 dilution; ab16794, Abcam), NeuN (1:500 dilution; ABN90P, Millipore), GFP (1:300 dilution, 132002, Synaptic Systems; 1:1,000 dilution, 600-401-215, Rockland; 1:500 dilution, 600-101-215, Rockland), GFAP (1:1,000 dilution; 173004, Synaptic Systems), PDGRα (1:200 dilution; APA5, BD Pharmingen), Aldh1l1 (1:200 dilution; ab87117, Abcam), MHCII (1:400 dilution; eBiosciences, 14-5321-82) and laminin-α (1:500 dilution; af3837, R&D Systems). Subsequently, the sections were incubated for 90 min at 20 °C with appropriate fluorophore-conjugated secondary antibodies (Thermo Scientific, BioLegend). After each antibody incubation, slices were washed three times for 10 min with 0.1% Triton X-100-PBS. All sections were incubated with DAPI (1:1,000 dilution) for 15 min and mounted with ProLong Gold (Invitrogen) or Fluoromount-G (Southern Biotech). Images were obtained using a Zeiss LSM 780 confocal microscope (×60 Apochromat/NA 1.4), an automated upright Leica DM5500 B microscope (×20 HC Plan-Apochromat/NA 0.70), a Nikon A1R Eclipse Ti confocal (×60 Apochromat/NA 1.4) or a Zeiss Axioscan Z.1 slide scanner (×20 Plan-Apochromat/NA 0.8) equipped with a Hamamatsu Orca Flash 4.0 V3 camera. Axioscan images were exported and compressed to 10% file size. Fluorescence measurements were corrected for background. Subsequent image processing was performed using the National Institutes of Health ImageJ software (https://imagej.nih.gov/ij/download.html).

Structural integrity of the blood–brain barrier was assessed following transcardial perfusion of mice with ice-cold 4% PFA-PBS. Subsequently, brains were extracted from the skull and split into two halves (in the midsagittal plane). The right hemispheres were embedded in Frozen Section Medium (Thermo Fisher) immediately in cryomolds (Sakura), which were frozen on dry ice and stored at −80 °C until further use. The left hemispheres were postfixed overnight in 4% PFA-PBS at 4 °C. After dehydration, samples were embedded in paraffin in cryomolds and stored at 20 °C until further use. The brains were cut into 5-μm slices for paraffin sections (HM 340 E, Thermo Fisher) or 20-μm slices for cryosections (CryoStar NX70, Thermo Fisher). Cryopreserved sections were used to stain for ZO-1 (1:500 dilution; 617300, Invitrogen), claudin-1 (1:200 dilution; 51-9000, Thermo Fisher), E-cadherin (1:500 dilution; 610181, BD) and CD31 (1:100 dilution; DIA-310, Dianova). Paraffin sections were used to stain for occludin (1:100 dilution; 33-1500, Invitrogen) and CD31 (1:100 dilution; DIA-310, Dianova). Sections were permeabilized in 0.3% Triton X-100-PBS. Following blocking with 5% normal goat serum in 0.3% Triton X-100-PBS at 20 °C for 1 h, sections were incubated with primary antibodies in blocking solution and left at 4 °C overnight. After washing with PBS, sections were stained with fluorophore-conjugated secondary antibodies (Alexa Fluor-488 goat anti-rabbit/Alexa Fluor-488 goat anti-mouse (1:400 dilution; A11008/A11001, Thermo Fisher) or Alexa Fluor-633 goat anti-rat (1:400 dilution; A21094, Thermo Fisher)) in PBS or 0.1% Triton X-100-PBS at 20 °C for 1–1.5 h. Counterstaining was done with Hoechst reagent (Sigma-Aldrich; 1:1,000 dilution in PBS). Confocal laser scanning microscopy was performed using a Zeiss LSM780 confocal microscope equipped with a ×40 objective (NA 1.4). Images files were exported and further analysis was performed in ImageJ.

### Surface morphology imaging

To visualize potential deformations and assess the overall shapes of mouse brains, the entire organ was imaged in a Bioptonics 3001 OPT scanner. Samples were imaged using autofluorescence f-OPT^58^ and reflected light^59^. Four hundred images with a 0.9° angle pitch were acquired to image a full rotation of the sample. Consequently, 3D volumes were reconstructed using NRecon software (version 1.7.1.6; Bruker) and visualized with Arivis (version 2.12.5; Rostock).

### Single-cell RNA sequencing

Single-cell suspensions were prepared as described in the flow cytometry section. Actinomycin D (Sigma) 5 μM was added during cell isolation and staining procedures. Male mice aged between 12 and 16 weeks and from the same litter were used. Live CD11b^+^CD45^+^ and either CD4^+^CD45^+^CD11b^−^ or TCRβ^+^CD45^+^CD11b^−^ cells were sorted using a BD FACSAria III, and suspended in 0.04% BSA-PBS. After sorting, the cell number and viability were confirmed using a LUNA-FL dual fluorescence cell counter (Logos Biosystems). For each experiment, approximately 8,700 cells were added to each channel for a targeted cell recovery of 5,000 cells. After cell count and quality control, the samples were immediately loaded onto the 10x Genomics Chromium Controller and library preparation performed using the Single Cell 3′ Kit v3, according to manufacturer’s instructions. Library quality was checked at the recommended points using a Qubit 2 Fluorometer (Thermo Fisher) and a Bioanalyzer HS DNA kit (Agilent). Libraries were sequenced on an Illumina NovaSeq 6000 or Illumina HiSeq platform using the recommended paired-end sequencing workflow (v3 read parameters, 28-8-0-91 cycles). On average, libraries were sequenced to a depth of 50,000 reads per cell.

Data were preprocessed with Cell Ranger v.3.1 (αCamKII^IL-2^ dataset) or v.6.0 (PHP.*GFAP*-IL-2 dataset) from 10x Genomics. The resulting count matrices, showing the number of transcripts (unique molecular identifiers) for each gene in a given cell, were analyzed with R v.3.6.3 (https://www.r-project.org/)^60^ and Seurat (https://satijalab.org/seurat/; v.3.1.5)^61^ (αCamKII^IL-2^ dataset), or v.4.0.1 and v.4.0.5 (PHP.*GFAP*-IL-2 dataset), following the standard pipeline with default parameters, unless stated otherwise. Before analysis, the data were filtered based on different quality metrics calculated to only include *bona fide* single cells of high quality. Genes detected in less than five cells were filtered out. Low-quality cells or empty droplets (identified as those with less than 200 genes) and cells with less than 500 unique molecular identifier counts were also filtered out. Finally, libraries with extensive mitochondrial reads, indicative of dying cells, were filtered out. The feature expression measurements for each cell within the combined datasets were normalized by the total expression and log transformed. Before clustering, unwanted variation due to Unique Molecular Identifiers (UMI) number and mitochondrial gene expression was removed. A linear transformation (‘scaling’) of gene expression was also performed, normalizing across cells for variations in gene expression.

For the identification of various cell populations, dimension-reduction approaches were applied on gene expression data using the Seurat analysis package. Initially, linear dimension reduction was applied in the form of principal-component analysis, using the PCElbowPlot() function, to obtain the principal components, followed by dimension-reduction approaches, based on similarities in the expression data. *t*-SNE and UMAP reduction was used for non-linear dimensionality reduction on the subset of principal-component analyses representing the most variation in the gene expression data (determined via elbow plots). The A *k*-nearest neighbor algorithm was then applied to the projection to generate the shared nearest neighbor graph, which was used to generate the clusters using the FindClusters() function with the Louvain algorithm, using a resolution of 0.4 and 1,000 iterations. Thus, cells that are similar in gene expression cluster together in these ‘communities’. Once clusters were generated, expression of known marker genes was used to assign cell-type identity. Comparisons of cell proportions were calculated using a *t*-test with Bonferroni correction. Pathway analysis was performed using GAGE (v.2.40.2)^62^, Pathview (v.1.30.1)^63^ and clusterProfiler (v.3.18)^64^.

### High-sensitivity mouse IL-2 immunoassay

Serum was obtained from whole blood by incubation of blood at 20 °C for 30 min followed by centrifugation at 2,000*g* for 10 min. Serum samples were diluted (1:40) in assay dilution buffer (Life Technologies). Tissue samples (5 mg) were placed in 300 μl protein quant sample lysis buffer (Life Technologies) containing protease inhibitor cocktail (Life Technologies). Tissues were homogenized in a FastPrep instrument (MP Biomedicals) with Lysing Matrix D, according to the manufacturer’s recommendation, and then incubated on a shaker for 20 min at 4 °C. The lysate obtained was centrifuged at 16,000*g* for 1 min at 4 °C. IL-2 levels from tissue lysates and serum were examined using a ProQuantum High-Sensitivity mouse IL-2 immunoassay, according to the manufacturer’s instructions (Life Technologies).

### Functional imaging in acute brain slices

Brain slice procedures, including functional imaging of astrocytes and multi-electrode array electrophysiology of neurons, is described in the Supplementary Information.

### Quantification of blood–cerebrospinal fluid barrier and blood–brain barrier permeability

Blood–cerebrospinal fluid (CSF) barrier and blood–brain barrier permeability^65^ were determined by injecting intravenously with 75 mg per kg body weight of 4-kDa FITC-dextran (Sigma) 1 h before CSF collection. CSF was obtained from the fourth ventricle using the cisterna magna puncture method. Subsequently, mice were perfused with 0.2% heparin-PBS and brain tissue was isolated. CSF samples were diluted 100-fold in sterile PBS, and blood–CSF barrier leakage was determined by measurement of fluorescence at λ_ex_ of 485 nm and λ_em_ of 520 nm. Brain samples were cut into small pieces, incubated overnight at 37 °C in formamide while shaking. Supernatant was collected after centrifugation for 15 min at maximum speed. This supernantant was then diluted twofold in sterile PBS, and blood–brain barrier leakage was determined by measurement of fluorescence at λ_ex_ of 485 nm and λ_em_ of 520 nm.

### Statistics

Sample sizes were based off previously published studies with appropriate power. Data collection and analysis were performed blind to the conditions of the experiments. Mice were randomized into treatment groups at experimental initiation. Data distribution was assumed to be normal. Exemplar histological images were selected that closely resembled expression patterns seen overall in the experimental group. Comparisons between two groups were performed using unpaired two-tailed Student’s *t*-tests. Post hoc Holm’s or Sidak’s multiple-comparisons tests were performed, when required. Two-way ANOVA was used when appropriate. Non-parametric testing was performed when data were not normally distributed (QQ plot for visual check and Shapiro–Wilk normality test on pooled residuals). The value of *n* reported within figure legends represents the number of animals, unless otherwise specified. Values are represented as the mean ± s.e.m., with differences considered significant when *P* < 0.05. Graphs were prepared with GraphPad Prism (GraphPad Software v9.2.0).

*t*-SNE, FlowSOM and heat map analysis were performed in R (version 3.6.2) using an in-house script (66[Roca et al, 2021 arXiv]). FlowSOM clusters are formed based on multi-marker similarity in a non-supervised manner. Clusters were annotated based on post-clustering comparison of marker expression, aligning the unique marker profile of each cluster to literature-based nomenclature. Key annotations for T_reg_ cell clusters included naïve (CD62L^hi^CD44^lo^), activated (CD62L^lo^CD44^hi^), resident (activated, plus enriched for expression of CD69, CD103, KLRG1 and ST2) and peripheral (Nrp1^−^), with additional clusters annotated based on the unique marker distribution. Differences between *t*-SNE plots were calculated following the same approach as in the *t*-SNE algorithm (manuscript in preparation). From these point probabilities, the distribution of cross-entropy in the *t*-SNE space relative to the original space was obtained per plot. Then, all pairwise comparisons between plots were evaluated with Kolmogorov–Smirnov tests on the differences between the cross-entropy distributions. Resulting *P* values were corrected with the Holm method. Dendrograms were obtained from hierarchical clustering, using the Kolmogorov–Smirnov statistic as a distance measure^66^.

### Reporting Summary

Further information on research design is available in the Nature Research Reporting Summary linked to this article.

## Online content

Any methods, additional references, Nature Research reporting summaries, source data, extended data, supplementary information, acknowledgements, peer review information; details of author contributions and competing interests; and statements of data and code availability are available at 10.1038/s41590-022-01208-z.

## Supplementary information

Supplementary Information Supplementary Figs. 1–16, Resources 1–3, methods, references and source data

Reporting Summary

Supplementary Video 1 **CD4**^**+**^ **T cells in wild-type brain.** 3D surface rendering of a wild-type perfused brain, stained for CD4 (green), Foxp3 (red), CD31 (white) and DAPI (blue). Representative video of a conventional CD4^+^ T cell and a T_reg_ cell, in the midbrain.

Supplementary Video 2 **Brain T**_**reg**_ **cells in αCamKII**^**IL-2**^ **brain.** 3D surface rendering of an αCamKII^IL-2^ perfused brain, stained for CD4 (green), Foxp3 (red), CD31 (white) and DAPI (blue). Representative video of a CD4^+^ T cell cluster, consisting of two conventional CD4^+^ T cells and four T_reg_ cells, in the midbrain.

Supplementary Video 3 3D surface rendering of a PHP.GFAP-GFP-treated perfused brain, stained for CD4 (green), Foxp3 (red), CD31 (white) and DAPI (blue). Representative video of a conventional CD4^+^ T cell and a T_reg_ cell, within the midbrain.

Supplementary Video 4 **Brain T**_**reg**_ **cells in a PHP.GFAP-IL-2-treated brain.** 3D surface rendering of a PHP.GFAP-IL-2-treated perfused brain, stained for CD4 (green), Foxp3 (red), CD31 (white) and DAPI (blue). Representative video of a CD4^+^ T cell cluster consisting of three T_reg_ cells, in the midbrain.

## Data Availability

The single-cell RNA-sequencing datasets generated in this study are available on the Gene expression Omnibus under accession codes GSE153427 and GSE179176. Material requests should be made to the corresponding authors. Source data are provided with this paper.

## Source data

Source Data Fig. 1 Individual data points, statistical source data.
Source Data Fig. 2 Individual data points, statistical source data.
Source Data Fig. 3 Individual data points, statistical source data.
Source Data Fig. 4 Individual data points, statistical source data.
Source Data Fig. 5 Individual data points, statistical source data.
Source Data Fig. 6 Individual data points, statistical source data.
Source Data Fig. 7 Individual data points, statistical source data.
Source Data Fig. 8 Individual data points, statistical source data.
Source Data Extended Data Fig. 2 Individual data points, statistical source data.
Source Data Extended Data Fig. 3 Individual data points, statistical source data.
Source Data Extended Data Fig. 4 Individual data points, statistical source data.
Source Data Extended Data Fig. 5 Individual data points, statistical source data.
Source Data Extended Data Fig. 6 Individual data points, statistical source data.
Source Data Extended Data Fig. 7 Individual data points, statistical source data.
Source Data Extended Data Fig. 8 Individual data points, statistical source data.
Source Data Extended Data Fig. 9 Individual data points, statistical source data.

## Code availability

αCamKII^IL-2^ analysis code is available in Supplementary Resource 2, and PHP.*GFAP*-IL-2 analysis code is available in Supplementary Resource 3.
