# Supplementary material for: Astrocyte-targeted gene delivery of interleukin 2 specifically increases brain-resident regulatory T cell numbers and protects against pathological neuroinflammation
Source: Nat Immunol. 2022 May 26;23(6):878–91. doi: 10.1038/s41590-022-01208-z (PMC9174055; doi:10.1038/s41590-022-01208-z)
Supplement: Supplementary file 1 — Supplementary Figs. 1–16, Resources 1–3, methods, references and source data [file 41590_2022_1208_MOESM1_ESM.pdf]

nature portfolio

https://doi.org/10.1038/s41590-022-01208-z

Supplementary information

Astrocyte-targeted gene delivery of interleukin 2 specifically increases brain-resident regulatory T cell numbers and protects against pathological neuroinflammation

In the format provided by the authors and unedited

Supplementary Methods

Behavioral experiments

Behavioral experiments in healthy mice were performed in 13-18 weeks old female mice. Mice were habituated to their new environment for at least 5 days and tests were conducted during the light phase of their activity cycle. Behavioral experiments in TBI mice were initiated on day 16 post-TBI and were conducted during the dark phase of their activity cycle. The general health and weight of the mice were routinely recorded during the testing. Tests were performed and analyzed by an observer blind to the experimental group. If tracking error(s) occurred, mice were excluded. Behavioral experiments were recorded using infrared cameras. Sham operated littermates were used as controls.

Open-field: Open-field exploration was tested in a 40 cm × 40 cm × 30 cm (w×l×h) square arena illuminated by indirect light. Animals were dark adapted for 30 min and tested in the arena for 10 min. Movements of the mice in the arena were video-tracked for 10 minutes with Ethovision 7 software (Noldus).

Nest building: For the nest building test, mice were single-housed with 1.5 g of cocoon nesting pellets and left undisturbed for 24 hours. Nest building was scored on a 5-points scale. A score of 1 is given when the pellet is largely untouched and score of 5 when > 90% of the pellet is torn up, the nest is a crater, with walls on more than 50% of its circumference [1].

Sociability: Sociability was evaluated using the three-chamber test [2, 3]. The set-up consisted of a rectangular transparent plexiglass box divided into three compartments, which were separated by two partitions. The central chamber (42 x 26 cm) was connected to a left and right chamber (26 x 26 cm) connected by guillotine doors (6 x 8 cm). The test consisted of two consecutive stages: acclimation stage and sociability stage. After habituation to the central compartment (5 min), wire cages (11 x 10 cm) were placed in the external chambers, which either contained a 'stranger' mouse (same sex, 'stranger 1') or were left empty: approach behavior to either of the wire cages was recorded for 5 min (sociability). Animal behavior was recorded using a webcam and ANY-maze™ Video Tracking System software (Stoelting Europe).

Forced swim test: Mice were individually placed into a glass cylinder (20 × 14 cm) filled with water (16 cm depth, 25 ± 1°C). Individual mice were placed gently in the water and left for 6 min: the degree of immobility (passive floating, no active swimming or escape behavior) was scored for the final 4 min using ANY-maze™ Video Tracking System software (Stoelting Europe).

Light/dark test: The apparatus used for the light/dark test consisted of a cage (40 × 40 × 30 cm) divided into two compartments of equal size by a partition with a door. One compartment was brightly illuminated, while the other was dark. Mice were placed in the dark compartment and allowed to move freely between the two chambers for 10 min. Mouse activity in the light compartment was tracked with Ethovision 7 equipment and software (Noldus).

Rotarod test: Motor coordination and equilibrium were tested with a rotarod setup (MED Associates Inc.). Mice were first trained at constant speed (4 rpm for 2 min) and then tested on four trials (inter-trial interval, 10 min). During the test trials, the animals had to balance on a rotating rod that accelerated from 4 rpm to 40 rpm over a period of 300 seconds. The latency to fall off the rod was recorded (up to a 5 min cut-off point).

Contextual discrimination fear conditioning: Two identical conditioning boxes (25 × 25 × 25 cm) with stainless steel grid floors to deliver shocks were located in sound-attenuating dark cubicles. Animal movement was monitored by motion-sensitive platforms connected to an interfaced computer using Panlab Freezing v1.2.0 software. The degree of motion measured ranged from 0 to 100 (arbitrary units). Freezing, defined as the absence of movement except for breathing, was recorded when activity remained below a validated threshold of 2.5 arbitrary units for at least 1 second. Contextual fear conditioning was measured in three different contexts. Two contexts (A and C) were very similar in tactile (grid floor), olfactory (odor) and visual (dark) dimensions, but C had in addition an inserted 'A-frame' roof (adapted from [4]). In contrast, context B was different in all dimensions. On days 1 - 3, mice were placed in context A, and after 3 minutes a single mild foot shock (2 s, 0.5 mA) was applied to induce contextual fear conditioning. After another minute, the animals were placed back in their home cage. On days 4 and 5, fear memory to A, B, or C was measured by placing the animal for 4 minutes in each of the contexts and measuring the amount of

freezing. The amount of freezing in C compared to A indicated the amount of contextual generalization.

Morris water maze (MWM): Spatial learning and cognitive flexibility were tested in the hidden platform MWM. For the healthy mouse experiments, a circular pool (150 cm diameter) was filled with opaquified (0.01% Acusol OP301, Dow Chemicals) water (26 ± 1°C). The platform (15 cm diameter) was hidden 1 cm underneath the surface of the water. For spatial learning, the mice were trained for 10 days to a fixed platform position [5, 6]. To evaluate reference memory, probe trials (100 seconds) were conducted on days 6 and 11 during acquisition learning. During probe trials, floater mice were excluded. For cognitive assessment in TBI mice, a modified MWM was performed on days 19-26 post-TBI or sham surgery [7]. On day 19 mice were habituated to the water maze over 2 sessions lasting 1 minute each. Starting on day 20, the escape platform was positioned in the target quadrant and spatial cues were placed on the wall surrounding the maze. All animals underwent 2 sessions per day, with a 30 minute interval, for 5 consecutive days to assess spatial acquisition. During each session, mice were placed in randomized starting quadrants facing the maze wall. Trials lasted a maximum of 60 seconds, after which mice were placed on the escape platform if they did not succeed themselves. On day 26, a probe trial was carried out to assess reference memory. The escape platform was removed from the pool and mice were allowed to explore the maze for 60 seconds. For assessments in both healthy and TBI mice, swim paths were tracked with Ethovision 17 software (Noldus).

Novel object recognition: The novel object recognition (NOR) test [8] was performed on mice on day 17 post-TBI or sham surgery. The mice were habituated to the test environment on day 15, followed by training on day 16 (free exploration of the environment containing two identical objects for five minutes) and testing on day 17 (replacement of one familiar object by a novel object with five minutes of exploration). The ratio of time spent exploring the novel object over the familiar object was calculated and used as a proxy for learning and memory. Activity was tracked and semi-automatically quantified using Ethovision 14 software (Noldus).

Functional imaging in acute brain slices

Recordings were done in (i) αCamKII^{IL-2} mice and littermate controls (mixed gender, 2-3 months old, or (ii) female age-matched AAV injected mice (2-3 months old). Preparation of acute brain slices was adapted from a published protocol [9]. Briefly, animals were anesthetized using intraperitoneal administration of nembutal (50 mg/kg). Transcardial perfusion was performed using 20 ml of ice-cold N-methyl-D-glucamine-based artificial cerebrospinal fluid dissection solution (NMDG-ACSF), containing (in mM): 93 NMDG, 2.5 KCl, 1.25 NaH_2PO_4, 30 $NaHCO_3$, 10 $MgSO_4$, 0.5 $CaCl_2$, 20 HEPES pH 7.4, 25 D-glucose, 5 L-ascorbic acid, 2 thiourea, 3 sodium pyruvate, 10 N-acetyl-L-cysteine. The solution was adjusted to 305–310 mOsm/l, pH 7.4 (HCl) and bubbled in 95% O_2/5% CO_2 gas for 20 min before use and throughout the experiment. Following decapitation, the brain was swiftly removed. Coronal slices containing the visual cortex (350 µm thick) were obtained using a VT1200s vibratome (Leica Biosystems). Slices were further cut along the midline to separate the cerebral hemispheres, and placed in a chamber containing NMDG-ACSF at 33°C. Slices were maintained under these conditions for 25 min, with the controlled reintroduction of Na^+ achieved by gradual addition of 2 M NaCl to the chamber. Slices were then transferred to a storage chamber containing 20 °C holding ACSF (in mM): 92 NaCl, 2.5 KCl, 1.25 NaH_2PO_4, 30 $NaHCO_3$, 2 $MgSO_4$, 2 $CaCl_2$, 15 D-Glucose, 20 HEPES pH 7.4, 5 L-ascorbic acid, 2 thiourea, 3 sodium pyruvate, 10 N-acetyl-L-cysteine (pH 7.4 HCl; 95% O_2/5% CO_2 gas; osmolarity 305–310 mOsm/l), until they were used for dye loading and recordings. For recordings, we used recording ACSF (in mM): 1.24 NaCl, 2.5 KCl, 1.25 NaH_2PO_4, 26 $NaHCO_3$, 2 $MgSO_4$, 2 $CaCl_2$, 15 D-Glucose (pH 7.4 HCl; 95% O_2/5% CO_2 gas; osmolarity 305–310 mOsm/l). For imaging, slices were transferred to a specialized recording chamber and superfused with normal ACSF (including pharmacological reagents where appropriate) at 22°C.

To identify astrocytes, cells were labeled with sulforhodamine 101 (SR101). To avoid potential issues associated with high levels of SR101 loading (such as induction of seizure like activity [10]), slices were incubated for 20 min in a six-well culture dish containing 1 µM SR101 (Sigma-Aldrich) in holding ACSF at 33°C. Next, slices were loaded in a solution containing Fluo4-AM. The dye was supplied as a 50 µg ampoule (Thermo Fisher) and was solubilized using a mixture of 7 µl dimethyl sulfoxide (DMSO), 2 µl 20% Pluronic F-127 (Tocris Biosciences) in DMSO and 1 µl 0.5% Kolliphor EL (Sigma-Aldrich) in DMSO. The

ampoule was then incubated at 41°C with constant agitation (1400 RPM) for 15 min using a thermomixer. Concentrated Fluo4-AM was then added to a well containing 3 ml of standard ACSF, giving a final concentration of 15.2 µM of Fluo4-AM. Slices were loaded in this solution for 45–60 min at 35°C. At the end of this period, excess AM dye was removed by washing in 20 °C holding ACSF for at least 1 hour prior to imaging.

All functional imaging recordings followed the same protocol. A field of view containing the primary visual cortex was chosen. First, baseline activity was recorded for 60s. Next, (R)-(-)-phenylephrine hydrochloride, 50 µM (PHE, Tocris Bioscience) was bath applied. Slices were imaged over a total period of 300 s. Live imaging of cells in acute slices was performed using a two-photon imaging system (VIVO 2-Photon platform, Intelligent Imaging Innovations GmbH), equipped with a tunable multiphoton laser (MaiTai laser, Spectra-Physics). Imaging was performed using a Zeiss Axio Examiner Z1, equipped with a W PlanApochromat 20×/NA1.0 objective. To excite both SR101 and Fluo4, the excitation wavelength was tuned to 840 nm. Signals were detected using two fast-gated GaAsP PMTs (Hamamatsu Photosensor Modules H11706), equipped with either a 525/40 nm or 612/69 nm emission filter. The system was further equipped with a 580 nm edge BrightLine® single-edge imaging-flat dichroic beamsplitter (Semrock FF580-FDi01-28x38). Images were 512 × 512 pixels in size and acquired at a frequency between 3 and 3.5 Hz. Acquisition was controlled using Slidebook v.6 software (Intelligent Imaging Innovations GmbH). Laser power was limited to a maximum of 30 mW at the specimen. The focal plane used was usually 30–100 µm deep within the slice.

Images were initially processed using Fiji software with standard plugins to correct for image drift and noise. First, a sum projection of all frames was used to generate one image of the SR101 channel, which was then used to draw a region of interest (ROI) surrounding astrocyte cell bodies. The average fluorescence for each ROI per frame of the Fluo4 channel was then measured and exported to MATLAB (The Mathworks). Next, we used custom-written scripts to evaluate the relative variations in intracellular Ca^{2+}, estimated as changes in Fluo4 signal relative to the baseline ($\Delta F/F0$). The baseline (F0) was defined for each ROI to be the average fluorescence before phenylephrine was added to the recording chamber. We calculated the peak amplitude of the response to PHE, as well as the total area under the curve (AUC) of the $\Delta F/F0$ trace using custom MATLAB scripts.

Multi-electrode array electrophysiology

LTP recordings were done in littermate αCamKII^{IL-2} and control mice (mixed sex, 2-3 months old), or age-matched female AAV-injected mice (2-3 months old). For tissue preparation, mice were anesthetized with isoflurane and decapitated. Brains were rapidly removed and 300 μm thick parasagittal brain slices prepared using a Leica VT1200 vibratome. Slicing was performed in a sucrose-based cutting solution (ACSF) containing (in mM): 87 NaCl, 2.5 KCl, 1.25 NaH_2PO_4, 10 glucose, 25 $NaHCO_3$, 0.5 $CaCl_2$, 7 $MgCl_2$, 75 sucrose, 1 kynurenic acid, 5 ascorbic acid, 3 pyruvic acid (pH 7.4 (HCl) ; 95% O_2/5% CO_2 gas). Slices were allowed to recover at 34°C for 35 min, and then maintained at 20 °C in the same solution for at least 30 min before using. For recordings, slices were placed onto a multielectrode array (MEA 2100, Multi Channel Systems) and continuously perfused with artificial cerebrospinal fluid (aCSF) solution containing (in mM): 119 NaCl, 2.5 KCl, 1 NaH_2PO_4, 11 glucose, 26 $NaHCO_3$, 4 $MgCl_2$ and 4 $CaCl_2$ (pH 7.4 (HCl) ; 95% O_2/5% CO_2 gas; 34°C). Field excitatory post-synaptic potentials (fEPSPs) were recorded from Schaffer collateral-CA1 synapses by stimulating and recording from the appropriate (visually identified) electrodes. Input-output curves were recorded for each slice by applying single-stimuli ranging from 500 to 2750 mV with 250 mV increments. A stimulus strength that corresponds to 35% of the maximal response in the input-output curve was used for recordings. For long-term potentiation (LTP) experiments, stable fEPSPs were recorded for 30 minutes to establish a baseline. Next, we applied three high frequency trains (100 stimuli; 100 Hz) with 5 minutes intervals. Subsequently, post-LTP fEPSPs were measured every 5 minutes (average of three consecutive stimuli at 15 seconds intervals) for 55 minutes. Recordings were processed and analyzed using Multi Channel Experimenter software (Multi Channel Systems).

Supplementary references

1. Deacon, R. Assessing burrowing, nest construction, and hoarding in mice. *J Vis Exp*, e2607 (2012).

2. Nadler, J.J. *et al.* Automated apparatus for quantitation of social approach behaviors in mice. *Genes Brain Behav* **3**, 303-314 (2004).

3. Naert, A., Callaerts-Vegh, Z. & D'Hooge, R. Nocturnal hyperactivity, increased social novelty preference and delayed extinction of fear responses in post-weaning socially isolated mice. *Brain Res Bull* **85**, 354-362 (2011).

4. van Boxelaere, M., Clements, J., Callaerts, P., D'Hooge, R. & Callaerts-Vegh, Z. Unpredictable chronic mild stress differentially impairs social and contextual discrimination learning in two inbred mouse strains. *PLoS One* **12**, e0188537 (2017).

5. Callaerts-Vegh, Z. *et al.* Concomitant deficits in working memory and fear extinction are functionally dissociated from reduced anxiety in metabotropic glutamate receptor 7-deficient mice. *J Neurosci* **26**, 6573-6582 (2006).

6. D'Hooge, R. & De Deyn, P.P. Applications of the Morris water maze in the study of learning and memory. *Brain Res Brain Res Rev* **36**, 60-90 (2001).

7. Vorhees, C.V. & Williams, M.T. Morris water maze: procedures for assessing spatial and related forms of learning and memory. *Nat Protoc* **1**, 848-858 (2006).

8. Lueptow, L.M. Novel Object Recognition Test for the Investigation of Learning and Memory in Mice. *J Vis Exp* **126**, 55718 (2017).

9. Ting, J.T. *et al.* Preparation of Acute Brain Slices Using an Optimized N-Methyl-D-glucamine Protective Recovery Method. *J Vis Exp* **132**, 53825 (2018).

10. Rasmussen, R., Nedergaard, M. & Petersen, N.C. Sulforhodamine 101, a widely used astrocyte marker, can induce cortical seizure-like activity at concentrations commonly used. *Sci Rep* **6**, 30433 (2016).

Supplementary Resources available as: Liston, Adrian (2021), "Expansion of Treg cells and prevention of pathological neuroinflammation following local delivery of IL-2", Mendeley Data, V1, doi: 10.17632/24tw36gbcd.1 at https://data.mendeley.com/datasets/24tw36gbcd/1

Supplementary Resource 1. High resolution Treg localization in coronal sections of wildtype and αCamKII^{IL-2} mice. Healthy perfused mouse brains from wildtype and αCamKII^{IL-2} mice were compared by immunofluorescent confocal imaging. CD4 (green), Foxp3 (red), laminin α4 (vascular basement membrane, white) and DAPI (blue). Left, wildtype mouse. Right, αCamKII^{IL-2} mouse. High resolution allows zooming and panning for localization of Treg cells across the entire coronal section.

Supplementary Resource 2. Code for single-cell RNA-seq analysis for wildtype vs *αCamKII*$^{IL-2}$ mice. Code is provided in .pdf and .Rmd formats.

Supplementary Resource 3. Code for single-cell RNA-seq analysis for PHP.GFAP-GFP- vs PHP.GFAP-IL-2-treated mice. Code is provided in .pdf and .Rmd formats.

Supplementary Video 1. CD4 T cells in wildtype brain. 3D surface rendering of a wildtype perfused brain, stained for CD4 (green), Foxp3 (red), CD31 (white) and DAPI (blue). Representative video of a conventional CD4 T cell and a Treg, in the mid-brain region.

Supplementary Video 2. Brain Treg cells in αCamKII^{IL-2} brain. 3D surface rendering of an αCamKII^{IL-2} perfused brain, stained for CD4 (green), Foxp3 (red), CD31 (white) and DAPI (blue). Representative video of a CD4 T cell cluster, consisting of two conventional CD4 T cell and four Treg cells, in the mid-brain region.

Supplementary Video 3. CD4 T cells in control brain. 3D surface rendering of a PHP.GFAP-GFP-treated perfused brain, stained for CD4 (green), Foxp3 (red), CD31 (white) and DAPI (blue). Representative video of a conventional CD4 T cell and a Treg, within the mid-brain.

Supplementary Video 4. Brain Treg cells in PHP.GFAP-IL-2-treated brain. 3D surface rendering of a PHP.GFAP-IL-2-treated perfused brain, stained for CD4 (green), Foxp3 (red), CD31 (white) and DAPI (blue). Representative video of a CD4 T cell cluster consisting of three Treg cells, in the mid-brain region.

Supplementary Figure 1: Confocal identification of brain Treg cells in αCamKII^{IL-2} mice. Healthy perfused mouse brains from wildtype and αCamKII^{IL-2} mice were compared by immunofluorescent confocal imaging. CD4 (green), Foxp3 (red), CD31 (vasculature, white) and DAPI (blue). Single and combined channel representative images of CD4 T cells in the mid-brain, with close-up imaging of identified CD4 T cells. A representative picture of three individual mouse samples is shown (n = 3). Scale bar, 10 µm.

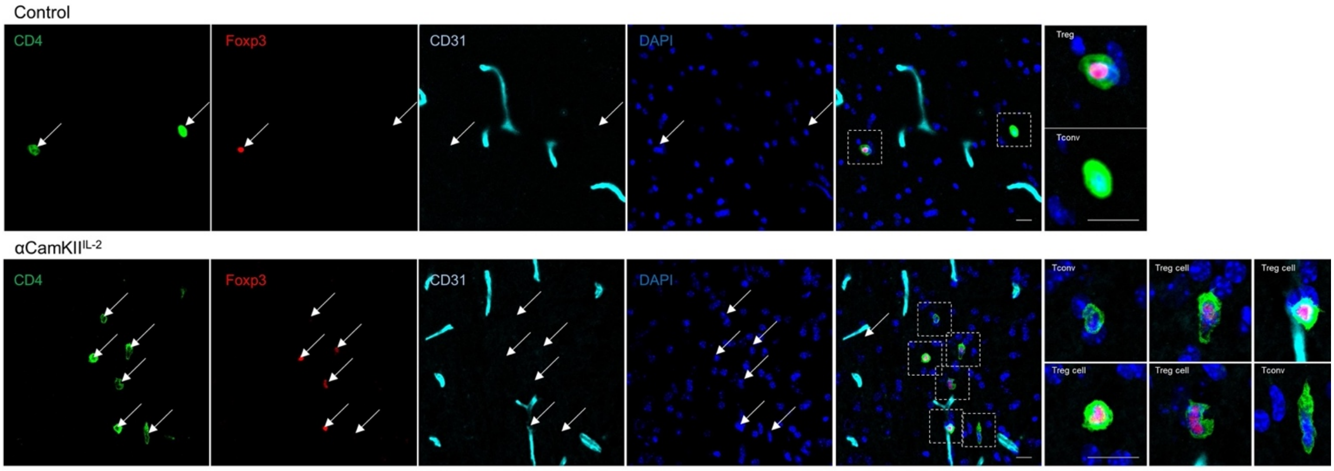

Supplementary Figure 2: Brain Treg localization in sagittal sections of αCamKII$^{IL\text{-}2}$ mice. Healthy perfused mouse brains from **a** wildtype and **b** αCamKII$^{IL\text{-}2}$ mice were compared by immunofluorescent confocal imaging. CD4 (green), Foxp3 (red), CD31 (vasculature, white) and DAPI (blue). Sagittal sections (top) were used to select large brain regions (main image). Arrowheads represent CD4 Tconv cells (blue) and Treg cells (red). Letters indicate selected cells shown in insets. A representative picture of three individual mouse samples is shown (n = 3). Scale bar main image, 100 µm. Scale bar insets, 10 µm.

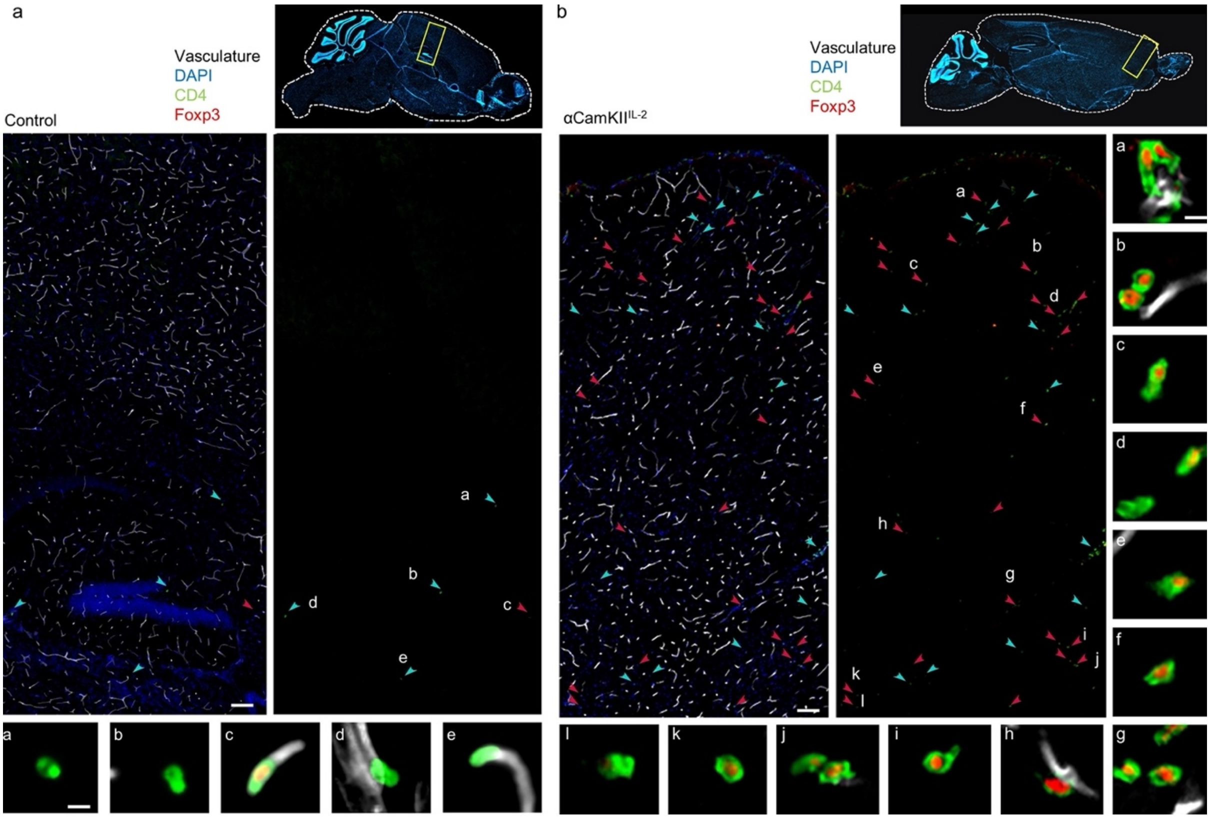

Supplementary Figure 3: Brain Treg localization in coronal sections of αCamKII^{IL-2} mice. Healthy perfused mouse brains from **a** wildtype and **b** αCamKII^{IL-2} mice were compared by immunofluorescent confocal imaging. CD4 (green), Foxp3 (red), laminin α4 (vascular basement membrane, white) and DAPI (blue). Entire coronal sections (left) were imaged, with the high resolution (single cell level) images available in Supplementary Resource 1. Five representative large regions were selected (middle), with identified CD4 Treg cells in these regions annotated by meningeal/perivascular (orange arrow) versus parenchymal (white arrow) localization. Insets (right) visualize identified cells. A representative picture is shown (n = 3, 1). Scale bar coronal section, 100 µm. Scale bar regional image, 100 µm. Scale bar insets, 10 µm.

A

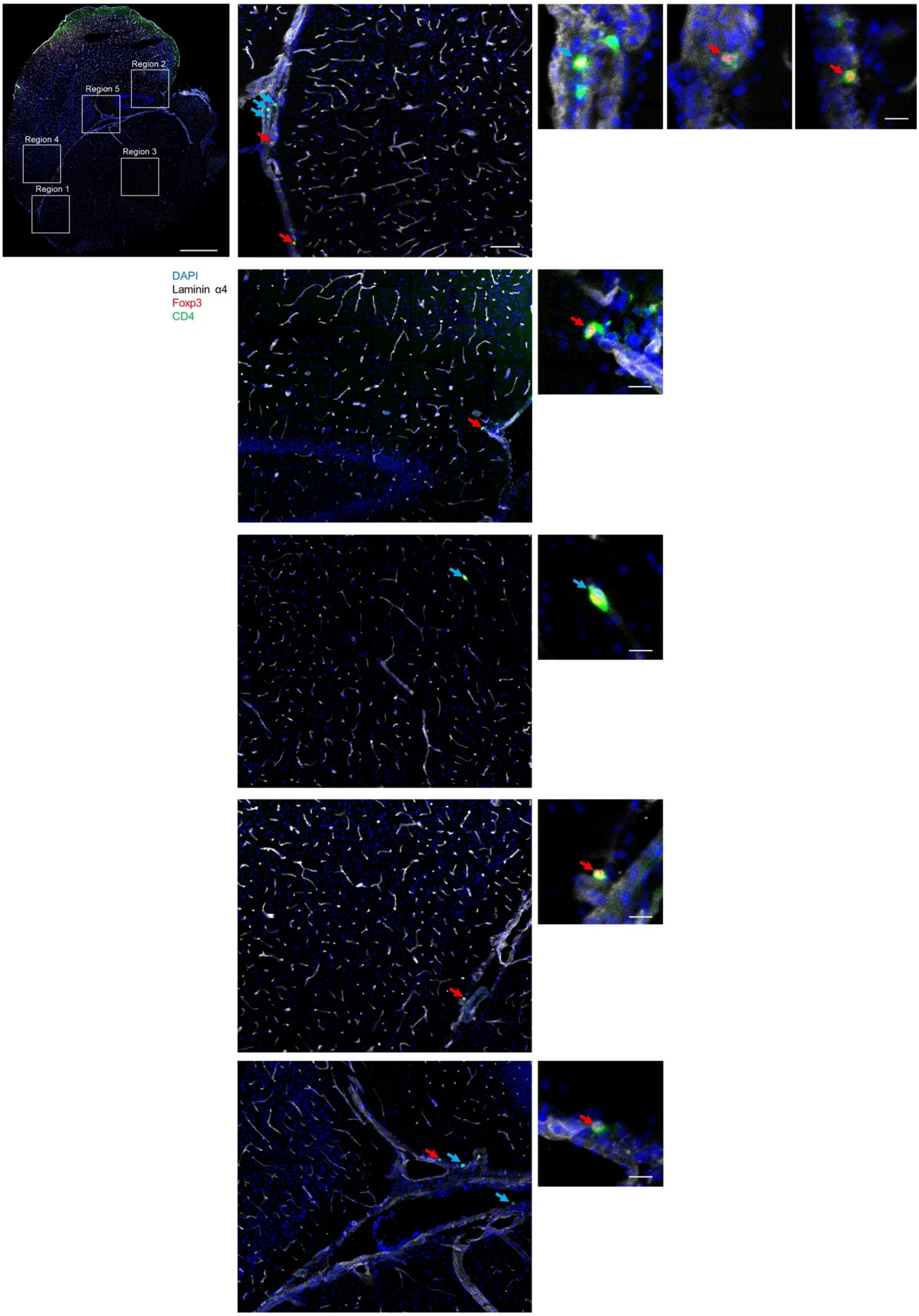
Region 2
Region 5
Region 4
Region 3
Region 1
DAPI
Laminin α4
Foxp3
CD4

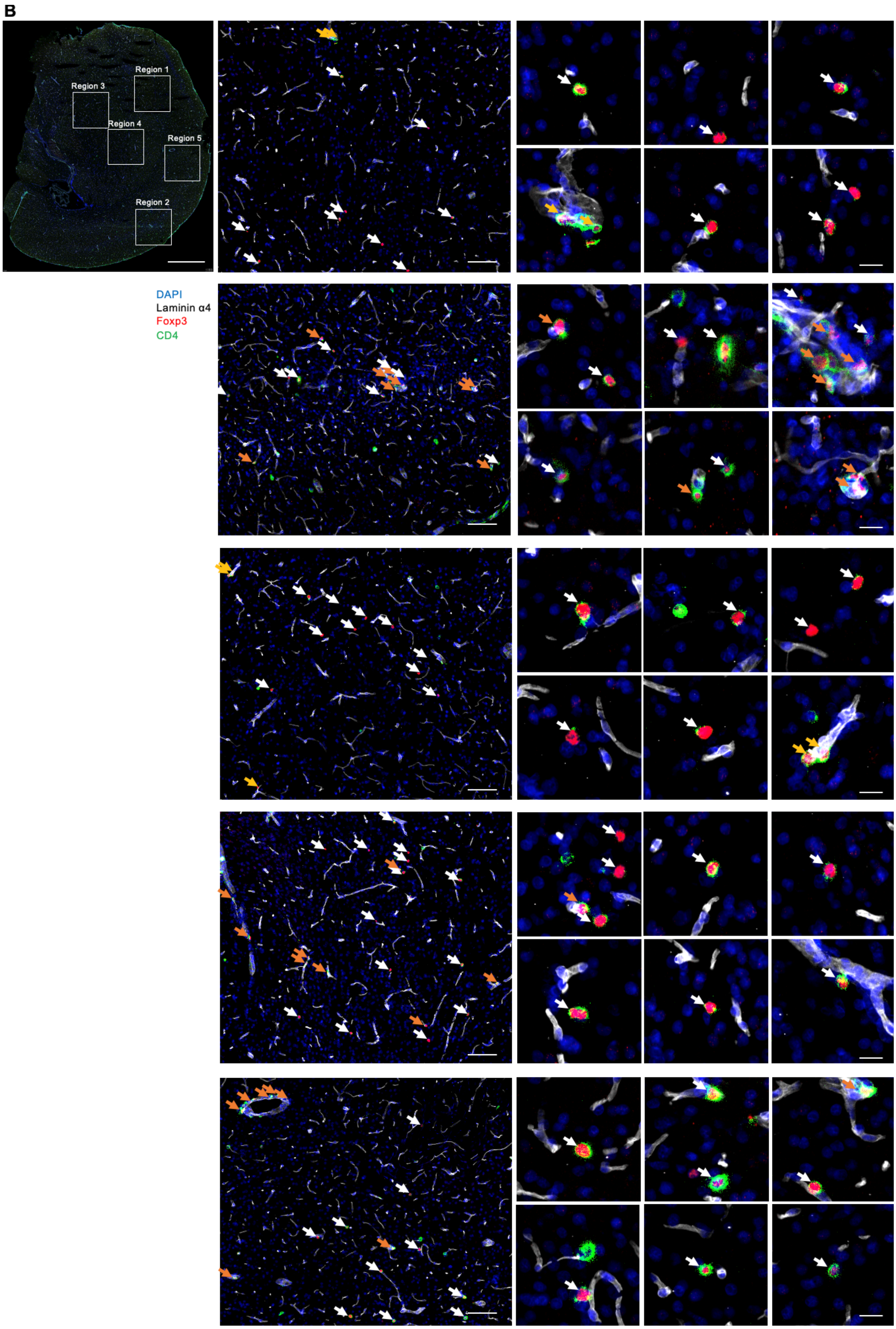
B
Region 1
Region 3
Region 4
Region 5
Region 2
DAPI
Laminin α4
Foxp3
CD4

Supplementary Figure 4: Treg cells in brain regions in αCamKII^{IL-2} mice. Healthy perfused brain from wildtype and αCamKII^{IL-2} mice was dissected into major regions for flow cytometric analysis of Treg cells (n = 5,3). **a**, Frequency of Treg cells within the CD4 population, and **b**, absolute numbers of Treg cells. Data are displayed as mean ± s.e.m.

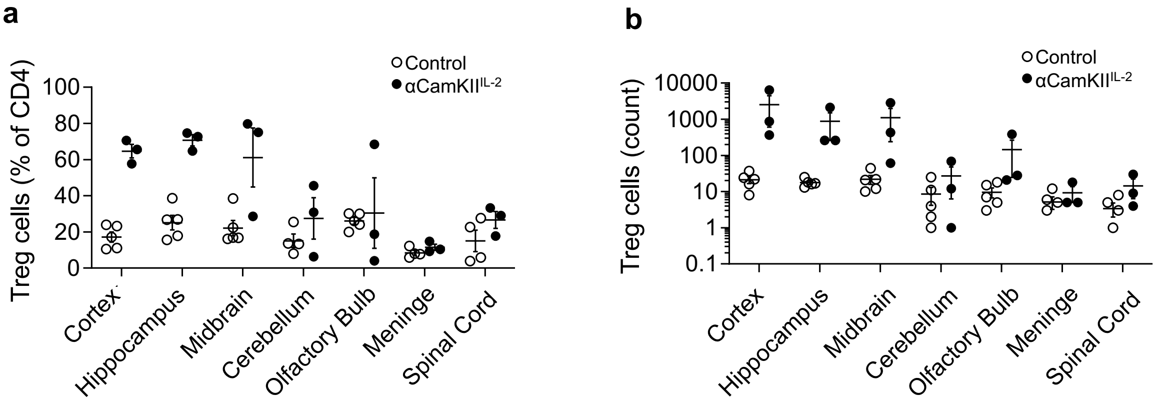

Supplementary Figure 5: Synthetic expansion of brain regulatory T cells preserves the subset distribution. Brain $CD11b^+$ cells, brain $CD45^+CD4^+$ cells and blood $CD45^+CD4^+$ positive cells were sorted from wildtype or $\alpha CamKII^{IL\text{-}2}$ mice for 10X single-cell sequencing (n=2). Data processing and filtering identified 28,590 cells, of which 20,021 were classified as microglia, 6332 were classified as T cells and 2237 were classified as non-target leukocytes. **a**, tSNE projection of the full dataset, with **b** quantification of clusters based on **c** the expression of key lineage markers, visualized as colored single cells on tSNE plots. **d**, clusters containing $CD4^+$ conventional and regulatory T cells were projected on a tSNE and reclustered, or **e** displayed based on origin from wildtype or $\alpha CamKII^{IL\text{-}2}$ mice. **f**, Quantification of clusters as a fraction of cells from each origin, with cluster annotation based on **g** key lineage markers.

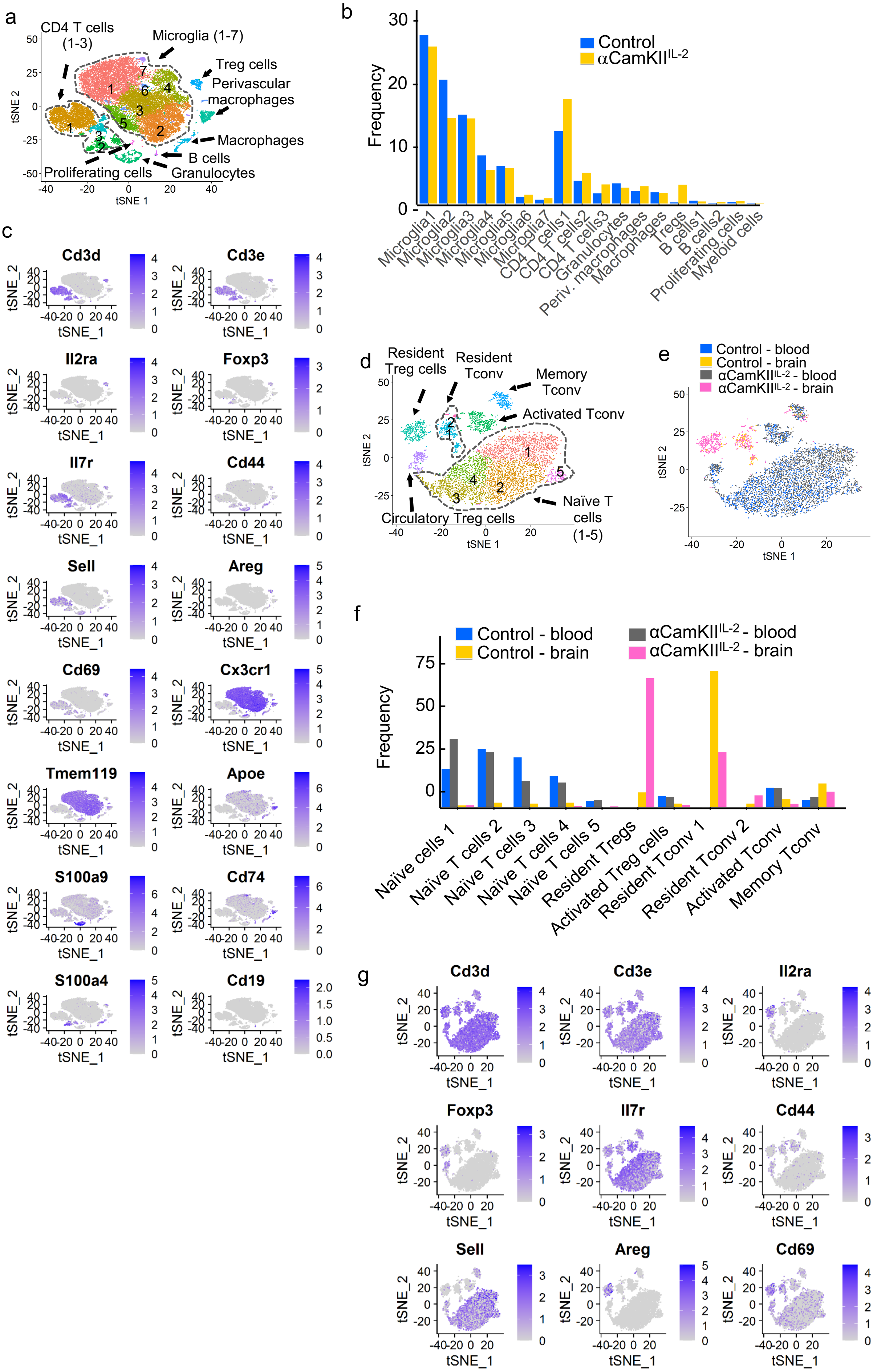
a
CD4 T cells (1-3)
Microglia (1-7)
Treg cells
Perivascular macrophages
Macrophages
B cells
Granulocytes
Proliferating cells
tSNE 1
tSNE 2
b
Frequency
Control
αCamKII^{IL-2}
Microglia1
Microglia2
Microglia3
Microglia4
Microglia5
Microglia6
Microglia7
CD4 T cells1
CD4 T cells2
CD4 T cells3
Granulocytes
Periv. macrophages
Macrophages
Tregs
B cells1
B cells2
Proliferating cells
Myeloid cells
c
Cd3d
Cd3e
Il2ra
Foxp3
Il7r
Cd44
Sell
Areg
Cd69
Cx3cr1
Tmem119
Apoe
S100a9
Cd74
S100a4
Cd19
tSNE_1
tSNE_2
d
Resident Treg cells
Resident Tconv
Memory Tconv
Activated Tconv
Naïve T cells (1-5)
Circulatory Treg cells
tSNE 1
tSNE 2
e
Control - blood
Control - brain
αCamKII^{IL-2} - blood
αCamKII^{IL-2} - brain
tSNE 1
tSNE 2
f
Control - blood
Control - brain
αCamKII^{IL-2} - blood
αCamKII^{IL-2} - brain
Frequency
Naïve cells 1
Naïve T cells 2
Naïve T cells 3
Naïve T cells 4
Naïve T cells 5
Resident Tregs
Activated Treg cells
Resident Tconv 1
Resident Tconv 2
Activated Tconv
Memory Tconv
g
Cd3d
Cd3e
Il2ra
Foxp3
Il7r
Cd44
Sell
Areg
Cd69
tSNE_1
tSNE_2

Supplementary Figure 6: Synthetic expansion of brain regulatory T cells preserves the transcriptional profile. Brain $CD11b^+$ cells, brain $CD45^+CD4^+$ cells and blood $CD45^+CD4^+$ positive cells were sorted from wildtype or $\alpha CamKII^{IL-2}$ mice for 10X single-cell sequencing (n=2). Data processing, filtering and reclustering (as in Supplementary Fig. 5) identified 6332 cells classified as T cells. **a**, Differential gene expression displayed for brain Treg cells from wildtype vs $\alpha CamKII^{IL-2}$ mice, **b** blood Treg cells from wildtype vs $\alpha CamKII^{IL-2}$ mice, **c** brain conventional T cells from wildtype vs $\alpha CamKII^{IL-2}$ mice, **d** blood conventional T cells from wildtype vs $\alpha CamKII^{IL-2}$ mice, or **e** blood vs brain Treg cells. Vertical lines mark fold changes 0.4 and -0.4 and horizontal lines mark the adjusted P value of 0.05. Selected significant changes are annotated. Statistical analyses were performed using Wilcoxon rank sum test to perform differential expression analysis. The P value adjusted is based on Bonferroni correction using all features in the dataset.

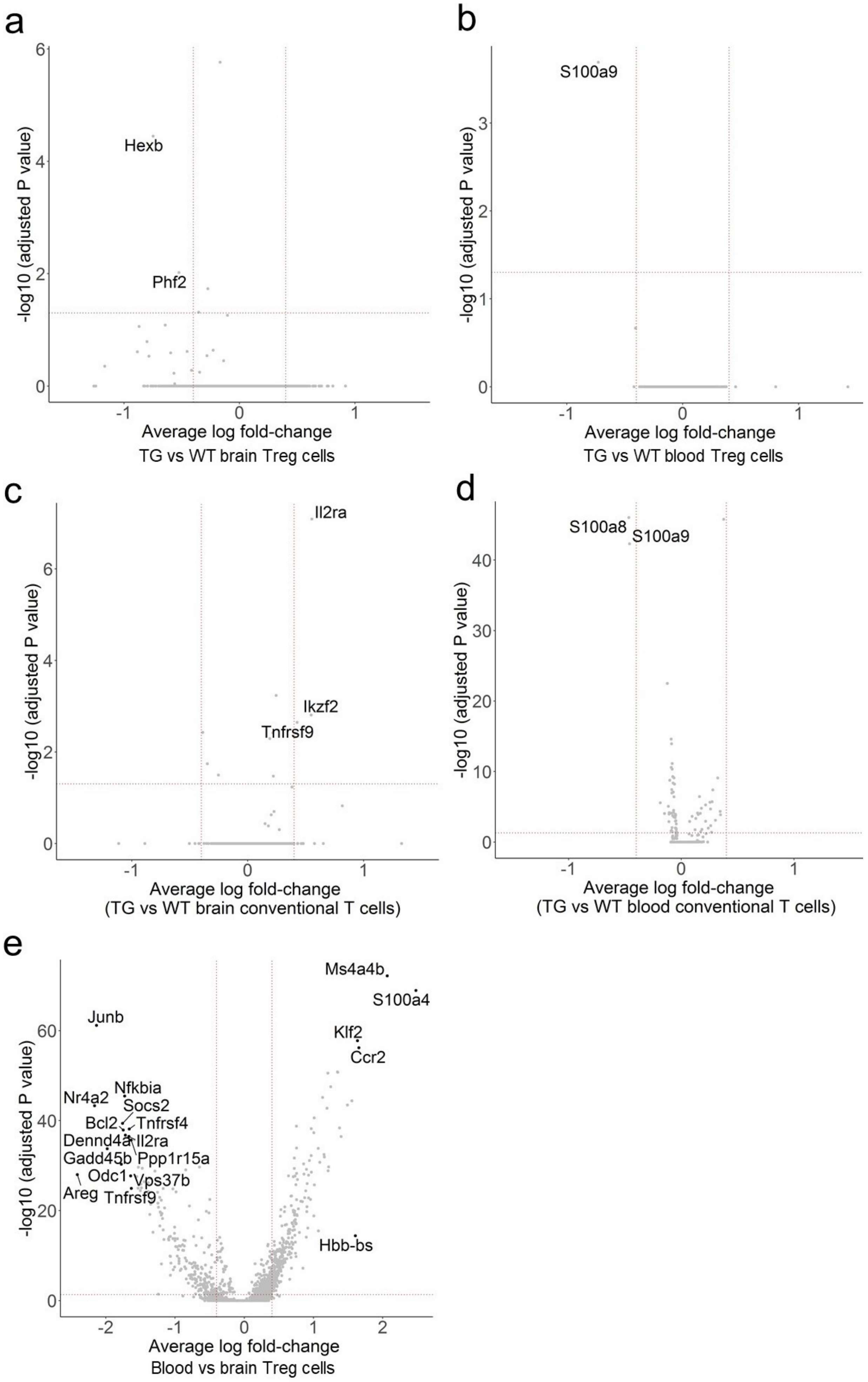
a
Hexb
Phf2
-log10 (adjusted P value)
Average log fold-change
TG vs WT brain Treg cells
b
S100a9
-log10 (adjusted P value)
Average log fold-change
TG vs WT blood Treg cells
c
Il2ra
Ikzf2
Tnfrsf9
-log10 (adjusted P value)
Average log fold-change
(TG vs WT brain conventional T cells)
d
S100a8
S100a9
-log10 (adjusted P value)
Average log fold-change
(TG vs WT blood conventional T cells)
e
Ms4a4b
S100a4
Junb
Klf2
Ccr2
Nfkbia
Nr4a2
Socs2
Bcl2
Tnfrsf4
Dennd4a
Il2ra
Gadd45b
Ppp1r15a
Odc1
Vps37b
Areg
Tnfrsf9
Hbb-bs
-log10 (adjusted P value)
Average log fold-change
Blood vs brain Treg cells

Supplementary Figure 7: Intact microglial transcriptional signature in αCamKII^{IL-2} mice. **a**, tSNE projection of 20,021 microglial cells from wildtype or αCamKII^{IL-2} mice. Following alignment, each cell was grouped into clusters denoted by number and color **b**, tSNE projection of microglial cells labelled as originating from control or αCamKII^{IL-2} mice, with **c** the proportion of cells from control or αCamKII^{IL-2} mice belonging to the identified populations. **d**, Volcano plots showing differentially expressed genes in wildtype vs αCamKII^{IL-2} microglial cells as annotated in panel A. Vertical lines mark fold changes 0.4 and -0.4 and horizontal line mark the adjusted P value of 0.05, genes above these thresholds are annotated. **e**, Visualization of expression of key lineage markers (colored single cells) on tSNE plots. **f**, Microglia from perfused brains of αCamKII^{IL-2} transgenic mice and littermate controls were assessed by high parameter flow cytometry. tSNE of microglia, gated on CD11b^{+} CX3CR1^{+} CD64^{+} CD45^{mod} Ly6G^{-} (n = 4/group). Microglia clusters, based on FlowSOM expression analysis, were assessed for expression of CD64, MHCII, TGFβ, LAMP1, CD44, CD69, PDL1, ST2, Ki67, CD80 and IL1β using a heatmap and dendrogram. **g**, Expression of key markers MHCII, PDL1 and CD80 visualized using tSNE projections. **h**, Frequency of expression of MHCII, TNF, TGFβ, LAMP1, PDL1, Ki67, CD80 and IL1β in control and αCamKII^{IL-2} microglia (n = 6, 11). Mean ± s.e.m. Statistical analyses were performed using an unpaired two-tailed Student's t-test with multiple test correction (h). Statistical analyses were performed using Wilcoxon rank sum test to perform differential expression analysis. The P value adjusted is based on Bonferroni correction using all features in the dataset (d).

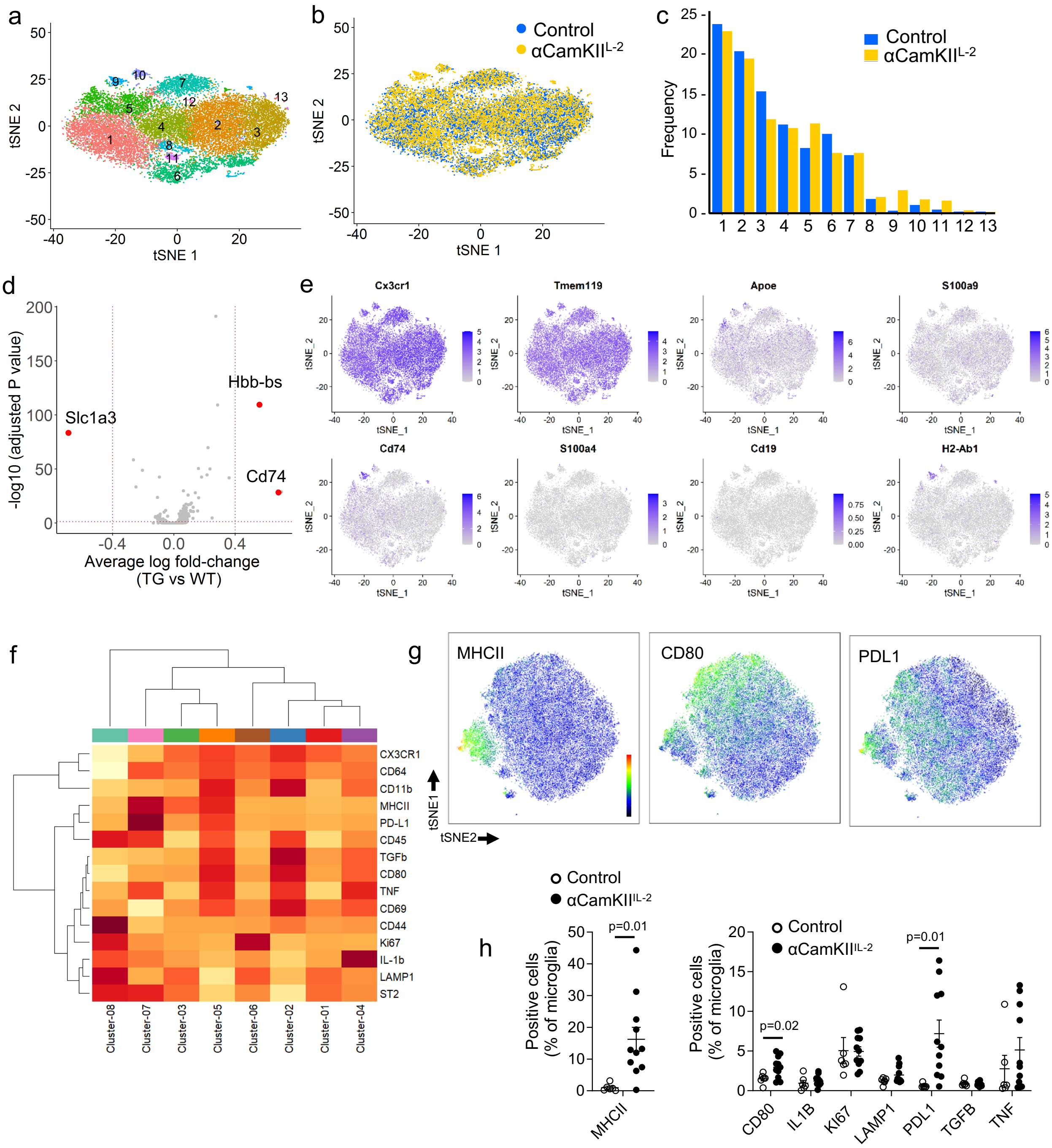
a
tSNE 2
tSNE 1
b
Control
αCamKII^L-2
tSNE 2
tSNE 1
c
Control
αCamKII^L-2
Frequency
d
Slc1a3
Hbb-bs
Cd74
-log10 (adjusted P value)
Average log fold-change
(TG vs WT)
e
Cx3cr1
Tmem119
Apoe
S100a9
Cd74
S100a4
Cd19
H2-Ab1
tSNE_1
tSNE_2
f
CX3CR1
CD64
CD11b
MHCII
PD-L1
CD45
TGFb
CD80
TNF
CD69
CD44
Ki67
IL-1b
LAMP1
ST2
Cluster-08
Cluster-07
Cluster-03
Cluster-05
Cluster-06
Cluster-02
Cluster-01
Cluster-04
g
MHCII
CD80
PDL1
tSNE1
tSNE2
h
Control
αCamKII^IL-2
p=0.01
Positive cells
(% of microglia)
MHCII
Control
αCamKII^IL-2
p=0.02
p=0.01
Positive cells
(% of microglia)
CD80
IL1B
KI67
LAMP1
PDL1
TGFB
TNF

Supplementary Figure 8: Progressive neurological damage following traumatic brain injury. Controlled cortical impact was performed to induce moderate TBI, with examination on days 1, 2, 3 and 7 post-TBI (n = 3, 3, 1, 3). **a**, Macroscopic damage to the surface of the brain at the injury site, representative photo. Scale bar, 0.5 cm. **b**, Representative immunofluorescence staining of the cortical tissue after controlled cortical impact. GFAP (astrocytes), Iba1 (microglia), DAPI (nuclei). Scale bars, 500 µm. **c**, Measure of total integrated GFAP intensity in the cortical area adjacent to the impact site at 1, 2, 3 and 7 days post-TBI. Mean ± s.e.m.

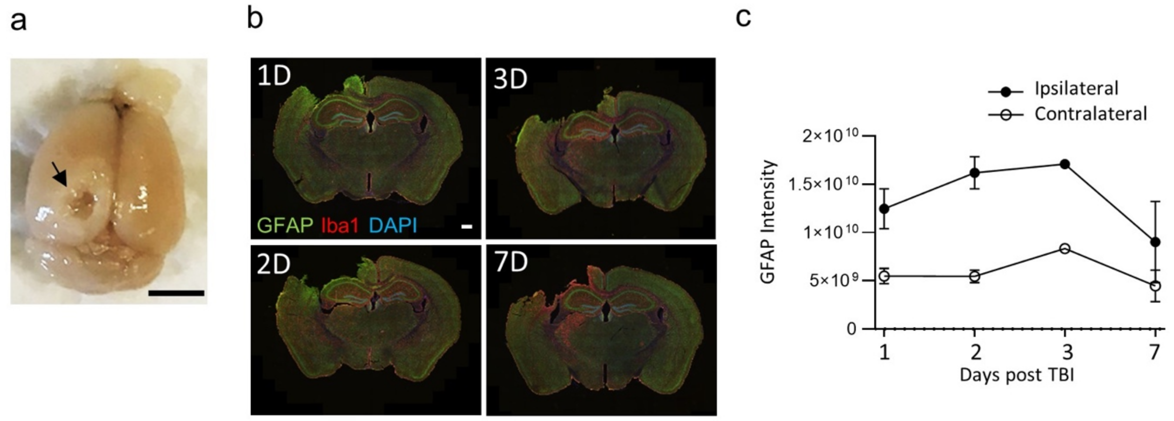

Supplementary Figure 9: Normal peripheral influx following traumatic brain injury in αCamKII^{IL-2} mice. Control (wildtype) littermates and αCamKII^{IL-2} mice were tamoxifen-treated at 6 weeks, and controlled cortical impacts to induce moderate TBI were given at 12 weeks. Mice were examined at 15 days post-TBI (n=4,4). TBI-induced perfused brains from littermate control and αCamKII^{IL-2} mice were compared by high-dimensional flow cytometry. **a**, Absolute number of CD4, Treg cells, CD8 and γδ T cells. **b**, Expression of CD25, CD44, CD69, Ki67 and PDL1 markers, or **c**, amphiregulin (Areg), IL10 and IL17 within the CD4 conventional T cell population. **d**, Expression of CD25, CD44, CD69, Ki67 and PDL1 markers, or **e** amphiregulin, IL10 and IL17 within the Treg population. Mean ± s.e.m. Statistical analyses were performed using a non-parametric Mann–Whitney U-test.

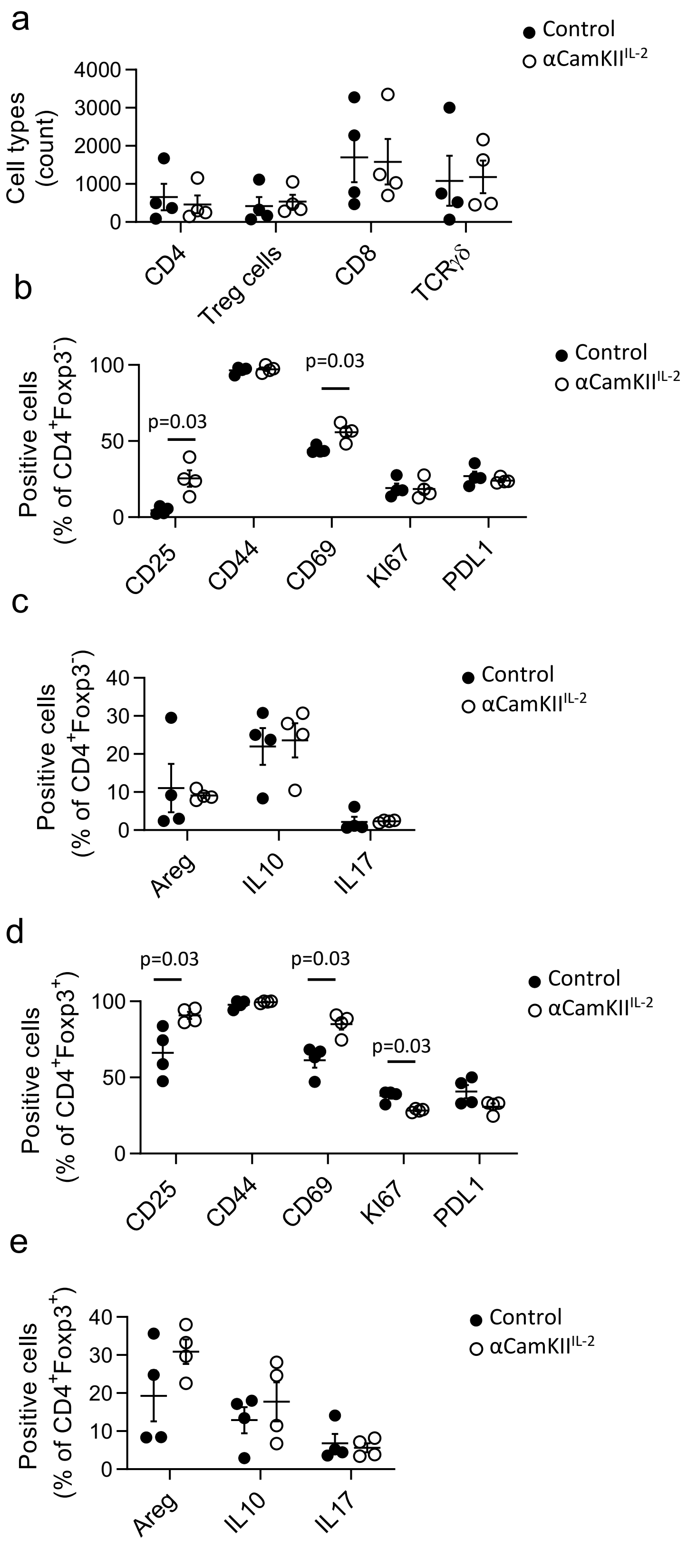

a
Control
αCamKII^IL-2
Cell types (count)
4000
3000
2000
1000
0
CD4
Treg cells
CD8
TCRγδ
b
Control
αCamKII^IL-2
Positive cells (% of CD4+Foxp3-)
100
50
0
p=0.03
p=0.03
CD25
CD44
CD69
KI67
PDL1
c
Control
αCamKII^IL-2
Positive cells (% of CD4+Foxp3-)
40
30
20
10
0
Areg
IL10
IL17
d
Control
αCamKII^IL-2
Positive cells (% of CD4+Foxp3+)
100
50
0
p=0.03
p=0.03
p=0.03
CD25
CD44
CD69
KI67
PDL1
e
Control
αCamKII^IL-2
Positive cells (% of CD4+Foxp3+)
40
30
20
10
0
Areg
IL10
IL17

Supplementary Figure 10: Confocal identification of brain Treg cells in PHP.*GFAP*-IL-2-treated mice. Healthy perfused mouse brains from wildtype mice treated with PHP.*GFAP*-GFP or PHP.*GFAP*-IL-2 were compared by immunofluorescent confocal imaging. CD4 (green), Foxp3 (red), CD31 (white) and DAPI (blue). Single and combined channel representative images of CD4 T cells in the mid-brain, with close-up imaging of identified CD4 T cells. A representative picture of three individual mouse samples is shown. Scale bar, 10 µm.

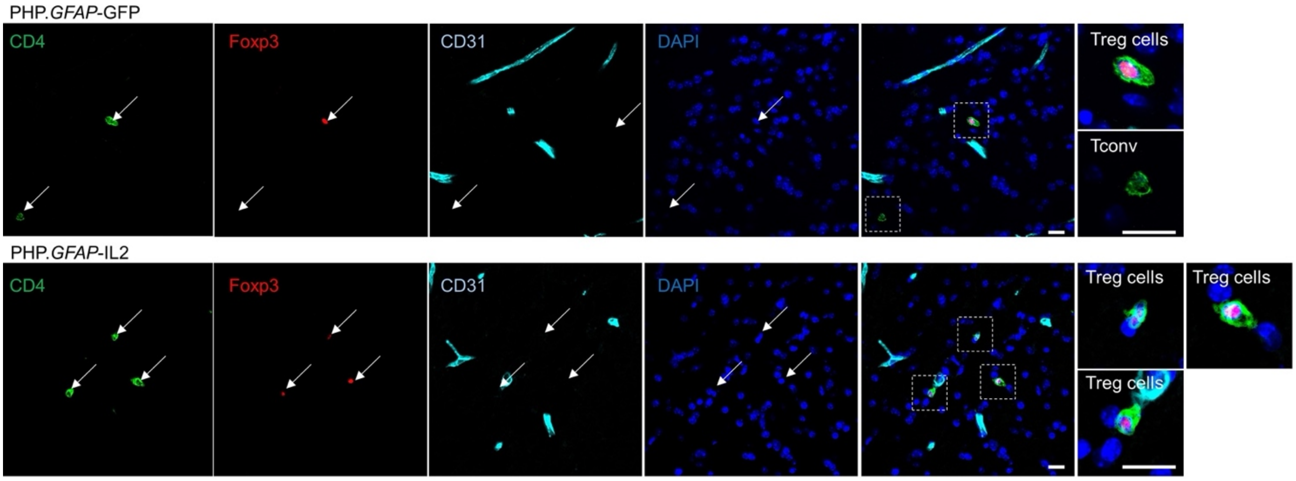

Supplementary Figure 11: Off-target effects of PHP.*GFAP*-IL-2 treatment are not observed. a, Healthy perfused brain, spleen and blood from PHP.*GFAP*-GFP control and PHP.*GFAP*-IL-2-treated mice were compared by high-dimensional flow cytometry (n = 3, 5). tSNE plots of leukocytes built on lineage markers (CD4, CD8, NK1.1, CD44, CD62L, CD69, CD25, Foxp3) with quantification of major populations. **b**, Absolute numbers of key leukocyte populations. **c**, tSNEs of brain CD4 conventional T cells built on key markers (CD62L, CD44, CD103, CD69, CD25, PD-1, Nrp1, ICOS, KLRG1, ST2, Ki67, CTLA4). Colors indicate annotated FlowSOM clusters, with quantification and **d** frequency of marker expression. **e**, tSNEs of brain CD8 T cells built on key markers (CD62L, CD44, CD103, CD69, CD25, PD-1, Nrp1, ICOS, KLRG1, ST2, Ki67, CTLA4). Colors indicate annotated FlowSOM clusters, with quantification and **f** frequency of marker expression. **g**, tSNEs of brain NK cells built on key markers (CD62L, CD44, CD103, CD69, CD25, PD-1, Nrp1, ICOS, KLRG1, ST2, Ki67, CTLA4). Colors indicate annotated FlowSOM clusters, with quantification and **h** frequency of marker expression. **i**, tSNEs of brain Treg cells built on key markers (CD62L, CD44, CD103, CD69, CD25, PD-1, Nrp1, ICOS, KLRG1, ST2, Ki67, CTLA4). Colors indicate annotated FlowSOM clusters, with quantification and **j** frequency of marker expression. Mean ± s.e.m. Statistical analyses were performed using an unpaired two-tailed Student's t-test. tSNE plots are compared using cross entropy tests.

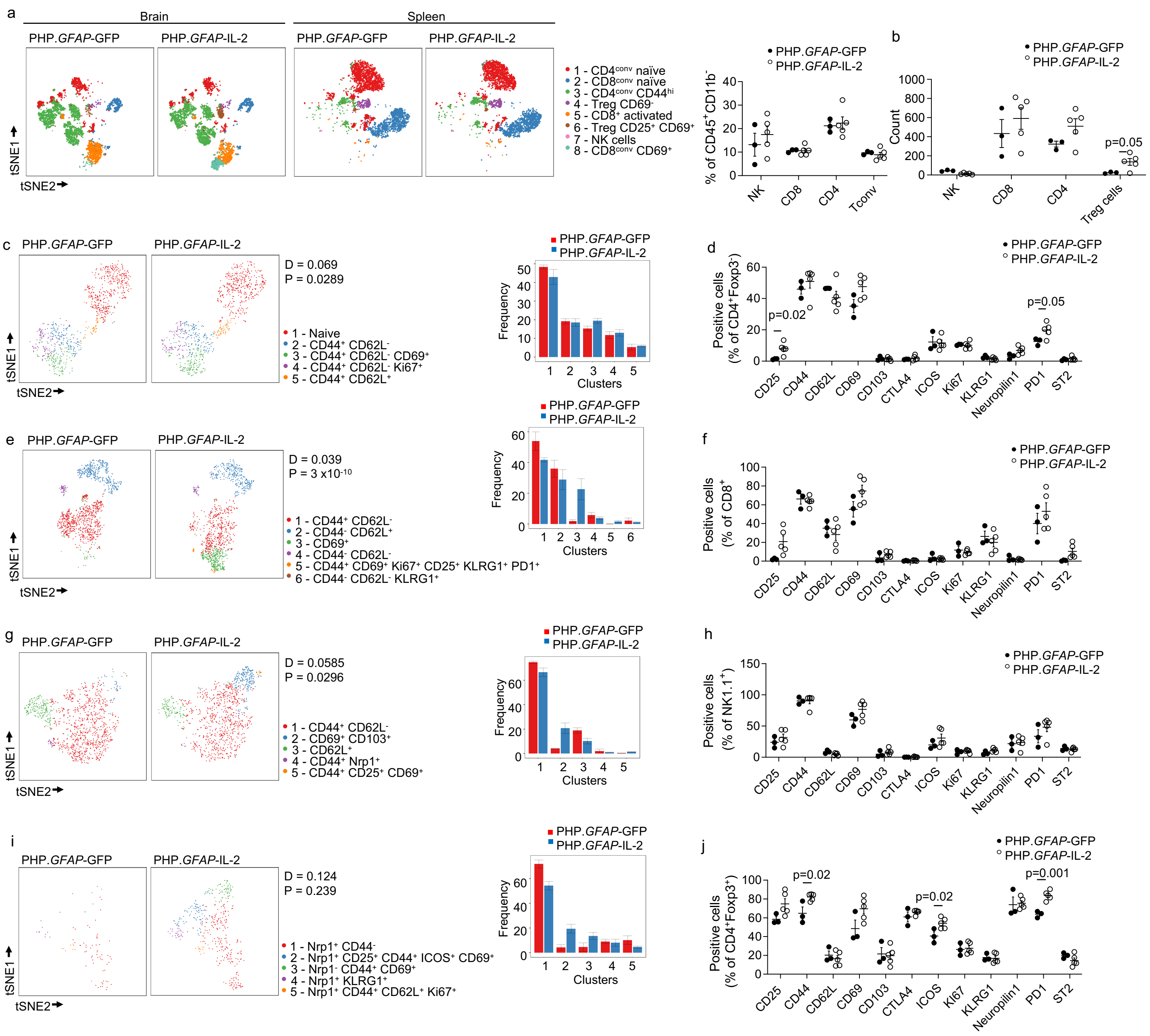
a
Brain
Spleen
PHP.GFAP-GFP
PHP.GFAP-IL-2
1 - CD4conv naïve
2 - CD8conv naïve
3 - CD4conv CD44hi
4 - Treg CD69-
5 - CD8+ activated
6 - Treg CD25+ CD69+
7 - NK cells
8 - CD8conv CD69+
tSNE1
tSNE2
% of CD45+CD11b-
NK
CD8
CD4
Tconv
b
Count
Treg cells
p=0.05
c
D = 0.069
P = 0.0289
1 - Naive
2 - CD44+ CD62L-
3 - CD44+ CD62L- CD69+
4 - CD44+ CD62L- Ki67+
5 - CD44+ CD62L+
Frequency
Clusters
d
Positive cells (% of CD4+Foxp3-)
p=0.02
p=0.05
CD25
CD44
CD62L
CD69
CD103
CTLA4
ICOS
Ki67
KLRG1
Neuropilin1
PD1
ST2
e
D = 0.039
P = 3 x10-10
1 - CD44+ CD62L-
2 - CD44- CD62L+
3 - CD69+
4 - CD44- CD62L-
5 - CD44+ CD69+ Ki67+ CD25+ KLRG1+ PD1+
6 - CD44- CD62L- KLRG1+
f
Positive cells (% of CD8+)
g
D = 0.0585
P = 0.0296
1 - CD44+ CD62L-
2 - CD69+ CD103+
3 - CD62L+
4 - CD44+ Nrp1+
5 - CD44+ CD25+ CD69+
h
Positive cells (% of NK1.1+)
i
D = 0.124
P = 0.239
1 - Nrp1+ CD44-
2 - Nrp1+ CD25+ CD44+ ICOS+ CD69+
3 - Nrp1- CD44+ CD69+
4 - Nrp1+ KLRG1+
5 - Nrp1+ CD44+ CD62L+ Ki67+
j
Positive cells (% of CD4+Foxp3+)
p=0.02
p=0.02
p=0.001

Supplementary Figure 12: Normal peripheral influx following PHP.*GFAP*-IL-2 treatment in traumatic brain injury mice. Mice treated with PHP.*GFAP*-GFP control or PHP.*GFAP*-IL-2 (day -14) were given controlled cortical impacts to induce moderate TBI and examined at 15 days post-TBI (n = 3, 4, 4); a sham TBI was included in the PHP.*GFAP*-GFP group. Perfused brains from sham, TBI and PHP.*GFAP*-IL-2-treated TBI mice were compared by high-dimensional flow cytometry. **a**, Absolute number of microglia, gated as $CD11b^{+}$ $CX3CR1^{+}$ $CD64^{+}$ $CD45^{mod}$ $Ly6G^{-}$ cells. **b**, CD8 and CD4 T cells, as a proportion of $CD45^{+}CD11b^{-}$ cells. **c**, Absolute number of Treg cells, CD4 Tconv and CD8 T cells. **d**, Frequency of CD25, CD44, CD69, Ki67 and PDL1 expressing-cells, and **e** frequency or **f** mean fluorescence intensity (MFI) of amphiregulin (Areg)-producing cells within the CD4 conventional T cell population. **g**, Frequency of CD25, CD44, CD69, Ki67 and PDL1 expressing-cells, and **h** frequency of IL-10 or Areg-producing cells, within the CD4 conventional T cell population. **i**, Mean expression of Areg, in Areg-producing CD4 conventional T cells. **j**, Representative histograms for CD25, CD44, CD69, Ki67, PDL1, IL-10 and Areg in CD4 conventional T cells, or **k** Treg cells, from sham, TBI and PHP.*GFAP*-IL-2-treated TBI mice. **l**, Mice treated with PHP.*GFAP*-GFP control or PHP.*GFAP*-IL-2 (day -14) were given controlled cortical impacts to induce moderate TBI and examined at 15 days post-TBI (n = 5/group). Superficial cervical lymph nodes were assessed for the percentage of CD4, CD8 and γδ T cells within the T cell compartment, and **m** Treg cells within the CD4 T cell compartment, **n** with calculation of absolute numbers. **o**, Cervical lymph nodes were assessed for the percentage of CD4, CD8 and γδ T cells within the T cell compartment, and **p** Treg cells within the CD4 T cell compartment, **q** with calculation of absolute numbers. Data are displayed as mean ± s.e.m. (**a** – **i**, **l** - **q**).

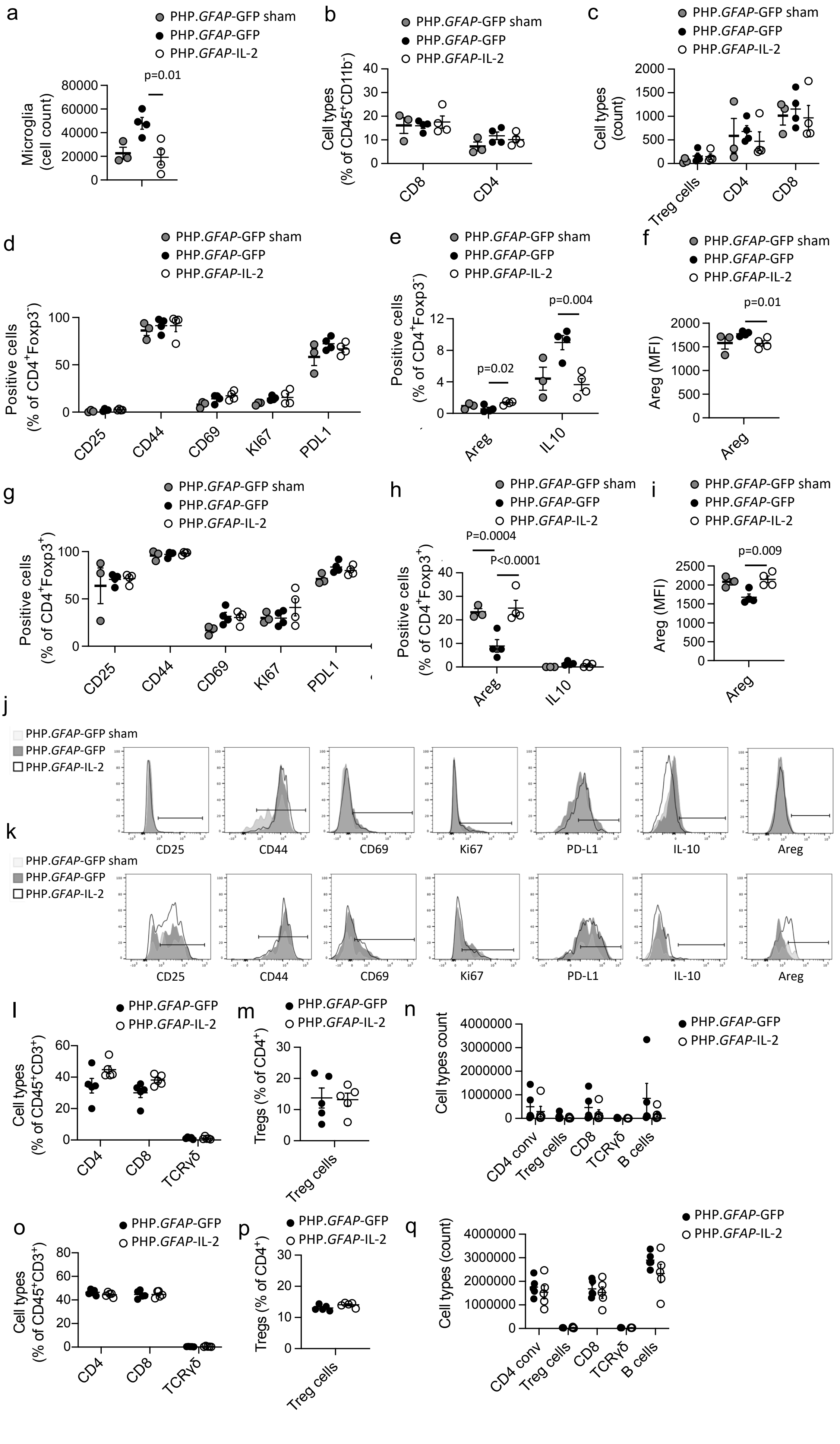

a
PHP.GFAP-GFP sham
PHP.GFAP-GFP
PHP.GFAP-IL-2
p=0.01
Microglia (cell count)
80000
60000
40000
20000
0
b
Cell types (% of CD45+CD11b-)
40
30
20
10
0
CD8
CD4
c
Cell types (count)
2000
1500
1000
500
0
Treg cells
CD4
CD8
d
Positive cells (% of CD4+Foxp3-)
100
50
0
CD25
CD44
CD69
KI67
PDL1
e
Positive cells (% of CD4+Foxp3-)
p=0.004
p=0.02
12
8
4
0
Areg
IL10
f
Areg (MFI)
p=0.01
2000
1500
1000
500
0
Areg
g
Positive cells (% of CD4+Foxp3+)
100
50
0
CD25
CD44
CD69
KI67
PDL1
h
Positive cells (% of CD4+Foxp3+)
P=0.0004
P<0.0001
40
30
20
10
0
Areg
IL10
i
Areg (MFI)
p=0.009
2500
2000
1500
1000
500
0
Areg
j
k
CD25
CD44
CD69
Ki67
PD-L1
IL-10
Areg
l
PHP.GFAP-GFP
PHP.GFAP-IL-2
Cell types (% of CD45+CD3+)
60
40
20
0
CD4
CD8
TCRγδ
m
Tregs (% of CD4+)
30
20
10
0
Treg cells
n
Cell types count
4000000
3000000
2000000
1000000
0
CD4 conv
Treg cells
CD8
TCRγδ
B cells
o
Cell types (% of CD45+CD3+)
80
60
40
20
0
CD4
CD8
TCRγδ
p
Tregs (% of CD4+)
30
20
10
0
Treg cells
q
Cell types (count)
4000000
3000000
2000000
1000000
0
CD4 conv
Treg cells
CD8
TCRγδ
B cells

Supplementary Figure 13: Normal peripheral influx during distal middle cerebral artery occlusion following PHP.*GFAP*-IL-2 treatment. Mice treated with PHP.*GFAP*-GFP control or PHP.*GFAP*-IL-2 (day -14) were subject to dMCAO and examined at day 14 (n = 3, 5, 2). Perfused brains were compared by high-dimensional flow cytometry. **a**, Absolute number of microglia, gated as $CD11b^{+}$ $CX3CR1^{+}$ $CD64^{+}$ $CD45^{mod}$ $Ly6G^{-}$ cells. **b**, Frequency of CD4, CD8 and γδ T cells within $CD45^{+}$ cells, and **c** frequency of Treg cells within $CD4^{+}$ T cells. **d**, Absolute numbers of CD4 Tconv, Treg cells CD8, and γδ T cells. **e**, Expression of CD25, CD44, CD69, Ki67 and PDL1 markers, or **f** amphiregulin (Areg), IL10 and IL17 within the CD4 conventional T cell population. **g**, Expression of CD25, CD44, CD69, Ki67 and PDL1 markers, or **h** Areg, IL10 and IL17 within the Treg population. **i**, Superficial cervical lymph nodes were assessed for the frequency of CD4 Tconv, Treg cells, CD8, Treg, B cells and γδ T cells within the $CD45^{+}$ cell population, with **j** absolute numbers. Mean ± s.e.m.

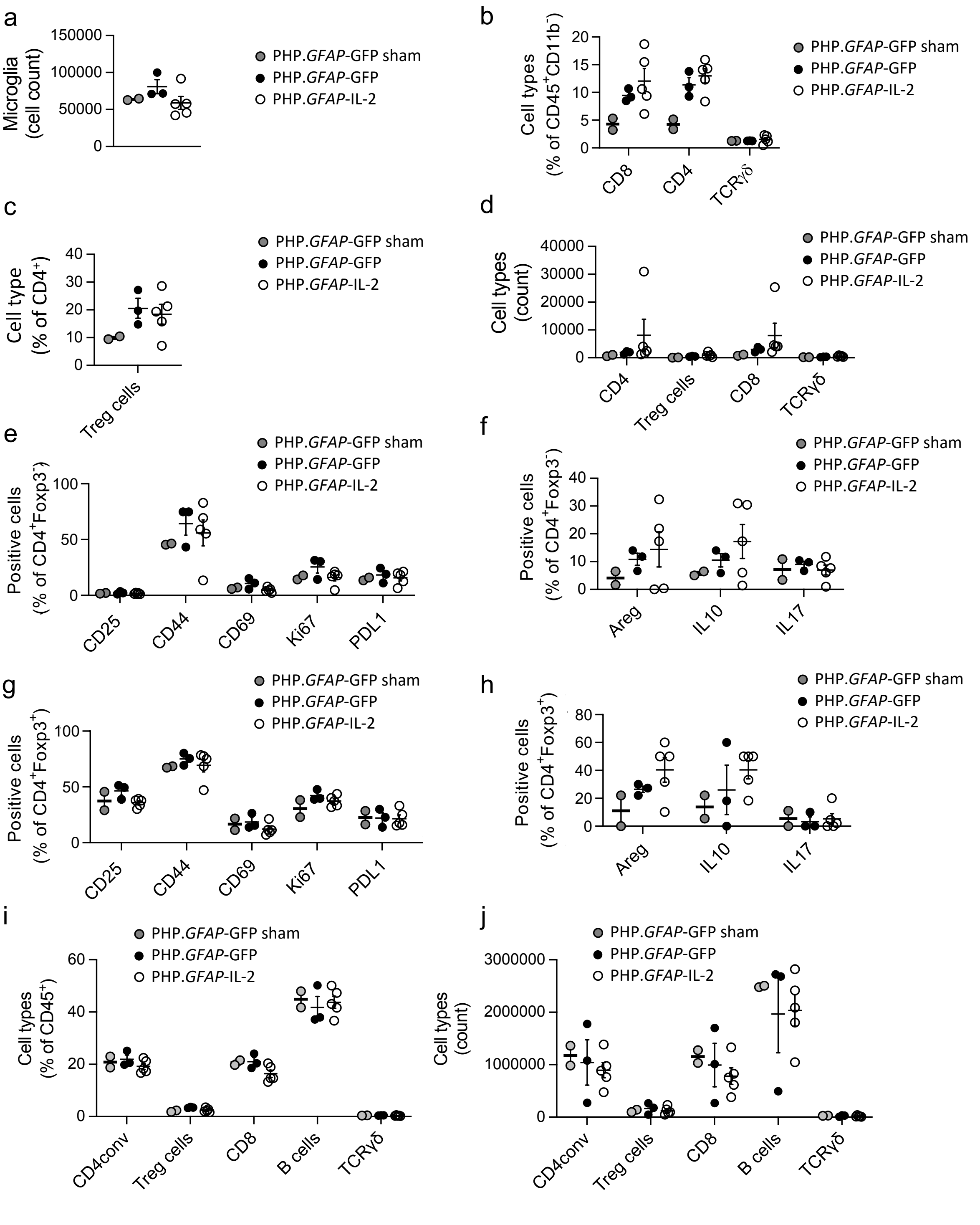
a
Microglia (cell count)
150000
100000
50000
0
PHP.GFAP-GFP sham
PHP.GFAP-GFP
PHP.GFAP-IL-2
b
Cell types (% of CD45+CD11b-)
20
15
10
5
0
CD8
CD4
TCRγδ
PHP.GFAP-GFP sham
PHP.GFAP-GFP
PHP.GFAP-IL-2
c
Cell type (% of CD4+)
40
30
20
10
0
Treg cells
PHP.GFAP-GFP sham
PHP.GFAP-GFP
PHP.GFAP-IL-2
d
Cell types (count)
40000
30000
20000
10000
0
CD4
Treg cells
CD8
TCRγδ
PHP.GFAP-GFP sham
PHP.GFAP-GFP
PHP.GFAP-IL-2
e
Positive cells (% of CD4+Foxp3-)
100
50
0
CD25
CD44
CD69
Ki67
PDL1
PHP.GFAP-GFP sham
PHP.GFAP-GFP
PHP.GFAP-IL-2
f
Positive cells (% of CD4+Foxp3-)
40
30
20
10
0
Areg
IL10
IL17
PHP.GFAP-GFP sham
PHP.GFAP-GFP
PHP.GFAP-IL-2
g
Positive cells (% of CD4+Foxp3+)
100
50
0
CD25
CD44
CD69
Ki67
PDL1
PHP.GFAP-GFP sham
PHP.GFAP-GFP
PHP.GFAP-IL-2
h
Positive cells (% of CD4+Foxp3+)
80
60
40
20
0
Areg
IL10
IL17
PHP.GFAP-GFP sham
PHP.GFAP-GFP
PHP.GFAP-IL-2
i
Cell types (% of CD45+)
60
40
20
0
CD4conv
Treg cells
CD8
B cells
TCRγδ
PHP.GFAP-GFP sham
PHP.GFAP-GFP
PHP.GFAP-IL-2
j
Cell types (count)
3000000
2000000
1000000
0
CD4conv
Treg cells
CD8
B cells
TCRγδ
PHP.GFAP-GFP sham
PHP.GFAP-GFP
PHP.GFAP-IL-2

Supplementary Figure 14: Normal peripheral influx during photothrombotic stroke following PHP.*GFAP*-IL-2 treatment. Mice treated with PHP.*GFAP*-GFP control or PHP.*GFAP*-IL-2 (day -14) were induced with a photothrombotic stroke and examined at day 1 (n = 3, 6, 6). Perfused brains were compared by high-dimensional flow cytometry. **a**, Absolute number of microglia, gated as CD11b$^+$ CX3CR1$^+$ CD64$^+$ CD45^{mod} Ly6G$^-$ cells. **b**, Frequency of CD4, CD8 and γδ T cells within CD45$^+$ cells, and **c** frequency of Treg cells within CD4$^+$ T cells. **d**, Absolute numbers of CD4 Tconv, Treg cells, CD8, and γδ T cells. **e**, Expression of CD25, CD44, CD69 and Ki67 markers, or **f** amphiregulin (Areg), IL10 and IL17 within the CD4 conventional T cell population. **g**, Expression of CD25, CD44, CD69 and Ki67 markers, or **h** Areg, IL10 and IL17 within the Treg population. Mean ± s.e.m.

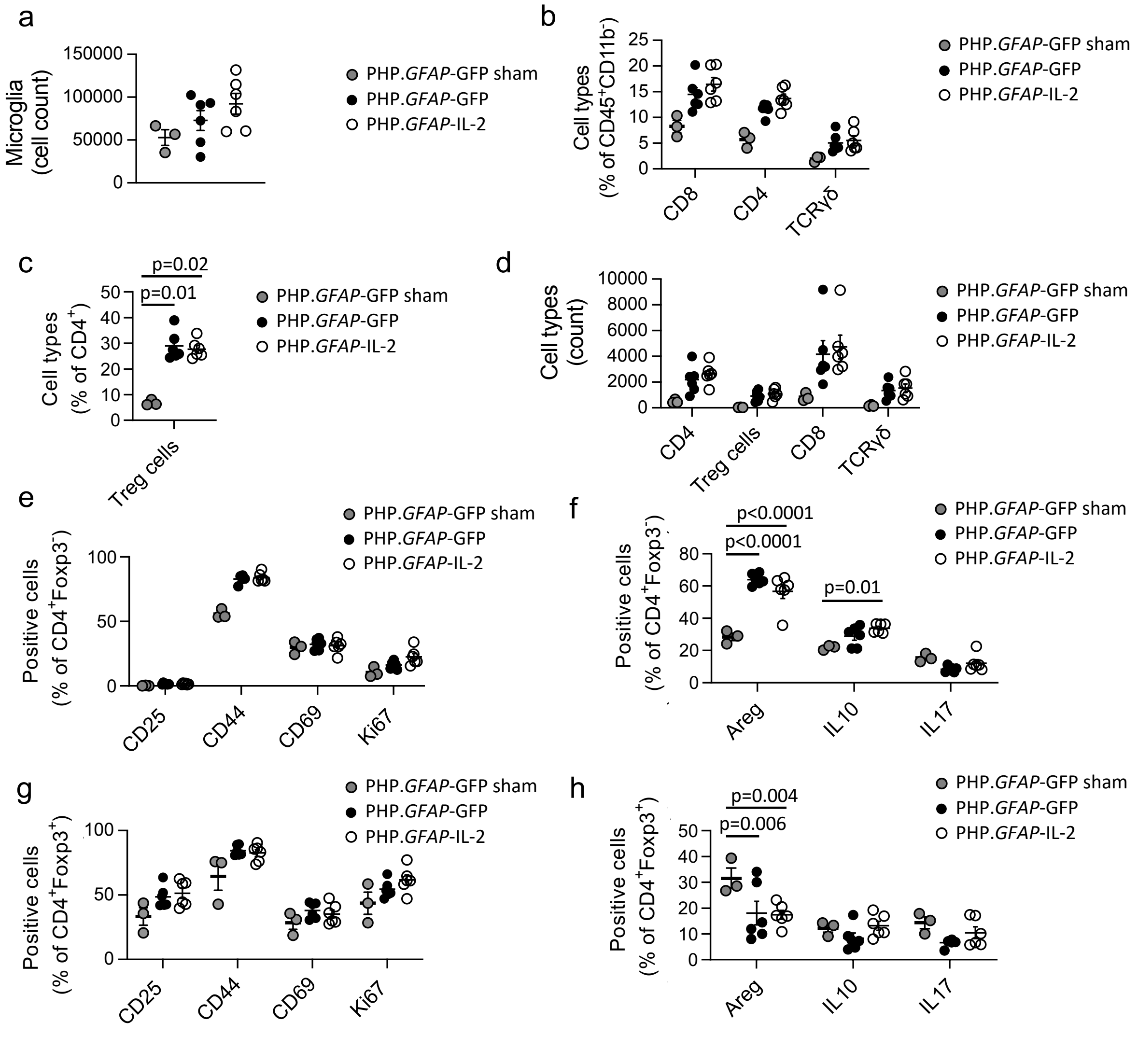

Supplementary Figure 15: Normal peripheral influx during experimental autoimmune encephalomyelitis following PHP.*GFAP*-IL-2 treatment. Mice treated with PHP.*GFAP*-GFP control or PHP.*GFAP*-IL-2 (day -14) were induced with experimental autoimmune encephalomyelitis (EAE) and examined at days 15, 21 and 30. Perfused CNS was compared by high-dimensional flow cytometry. **a**, Frequency of CD8 T cells (n = 5 D0; 5,5 D15; 10,10 D21; 7, 6 D30) and **b** CD4 T cells within $CD45^+$ cells (n = 5 D0; 5,5 D15; 10,10 D21; 7, 6 D30), and **c** frequency of Treg cells within $CD4^+$ T cells (n = 5 D0; 5,5 D15; 10,10 D21; 7, 6 D30). **d**, Absolute number of CD8 T cells (n = 5 D0; 5,5 D15; 10,10 D21; 7, 6 D30), **e** CD4 T cells (n = 5 D0; 5,5 D15; 10,10 D21; 7, 6 D30) and **f** Treg cells (n = 5 D0; 5,5 D15; 10,10 D21; 7, 6 D30). **g**, Frequency of CD25, CD44, CD62L, CD69, CD103, CTLA4, GITR, Helios, ICOS, Ki67, KLRG1, Neuropilin1, PD1 and Tbet expression within the CNS-resident Treg population on day 15 (n = 6, 5), **h** day 21 (n = 6, 5) or **i** day 30 (n = 7, 6). **j**, IL10 (n = 5, 5 D15 and D21; 7,5 D30) and **k** amphiregulin (Areg) expression within CNS-resident Treg cells (n = 5,5). **l**, IL17 (n = 5, 5 D15 and D21; 7,5 D30), **m** IFNγ (n = 5,5), **n** GM-CSF (n = 5, 5 D15 and D21; 7,5 D30), **o** TNFα (n = 5, 5 D15 and D21; 7,5 D30), **p** IL-2 (n = 5, 5 D15 and D21; 6,5 D30) and **q** IFNγ-TNFα cytokine expression within the CNS-resident Tconv population at various times post-EAE induction (n = 5,5). **r**, 15 days post-induction, major leukocyte subsets were assessed by flow cytometry in the superficial cervical lymph nodes (n = 5,5) and **s** the deep cervical lymph nodes (n = 5,5). **t**, Absolute numbers of major leukocyte subsets in the superficial and **u** deep cervical lymph nodes (n=5,5). Mean ± s.e.m. Statistical analyses were performed using an unpaired two-tailed Student's t-test.

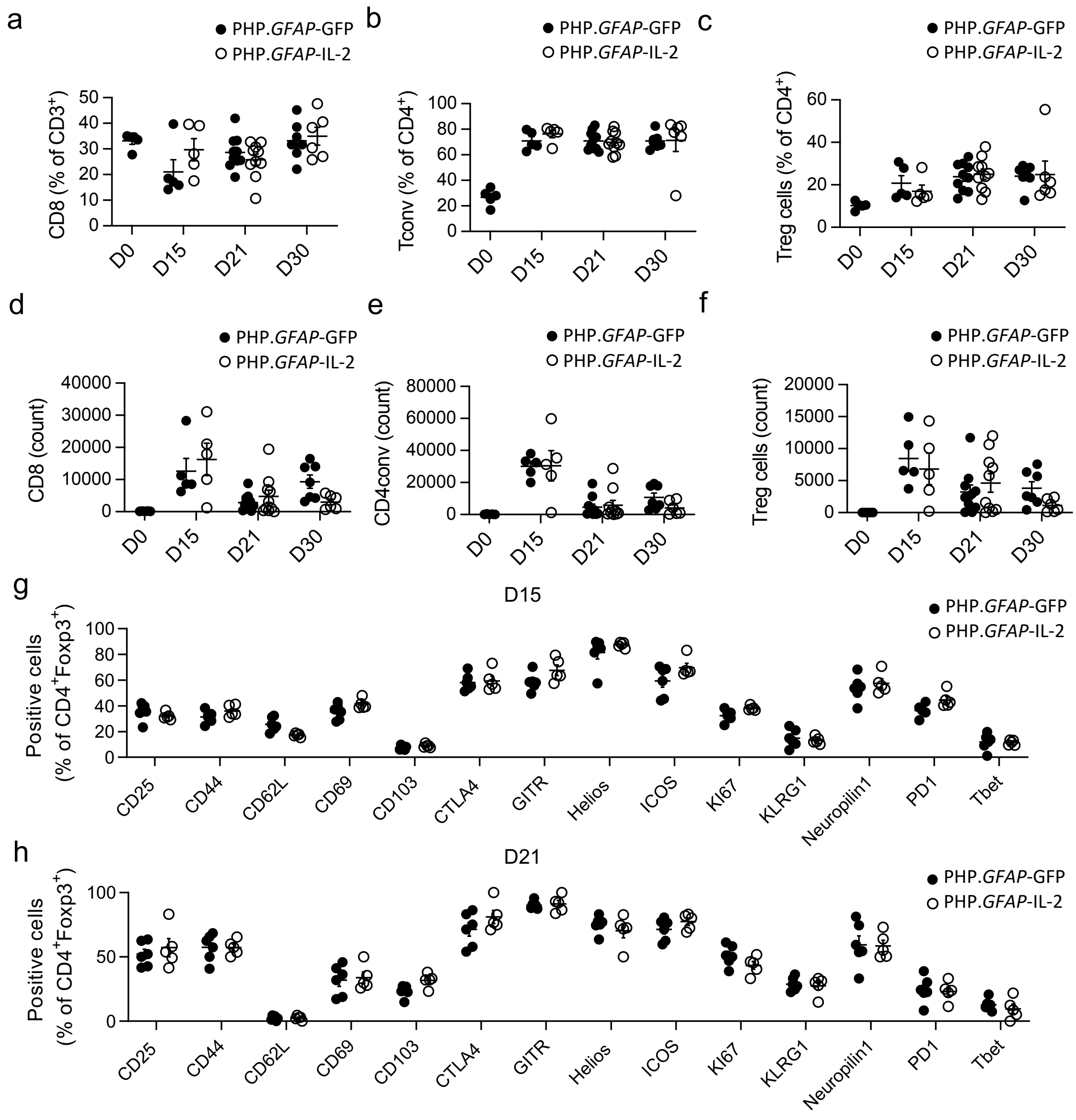

a
PHP.GFAP-GFP
PHP.GFAP-IL-2
CD8 (% of CD3+)
D0
D15
D21
D30
b
Tconv (% of CD4+)
c
Treg cells (% of CD4+)
d
CD8 (count)
e
CD4conv (count)
f
Treg cells (count)
g
D15
Positive cells
(% of CD4+Foxp3+)
CD25
CD44
CD62L
CD69
CD103
CTLA4
GITR
Helios
ICOS
KI67
KLRG1
Neuropilin1
PD1
Tbet
h
D21

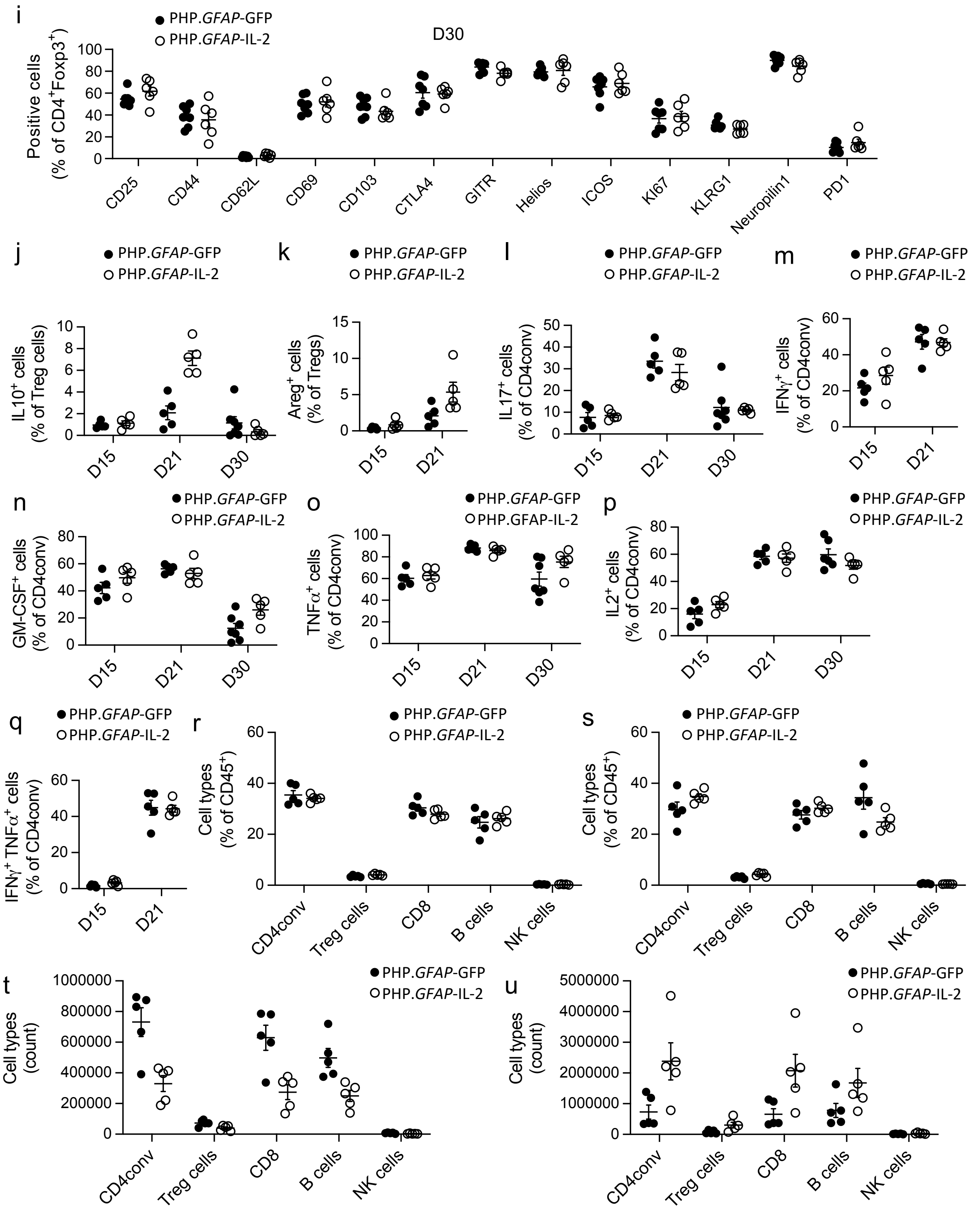
i
PHP.GFAP-GFP
PHP.GFAP-IL-2
D30
Positive cells (% of CD4+Foxp3+)
CD25 CD44 CD62L CD69 CD103 CTLA4 GITR Helios ICOS KI67 KLRG1 Neuropilin1 PD1
j
IL10+ cells (% of Treg cells)
k
Areg+ cells (% of Tregs)
l
IL17+ cells (% of CD4conv)
m
IFNγ+ cells (% of CD4conv)
D15 D21 D30
n
GM-CSF+ cells (% of CD4conv)
o
TNFα+ cells (% of CD4conv)
PHP.GFAP-IL-2
p
IL2+ cells (% of CD4conv)
q
IFNγ+ TNFα+ cells (% of CD4conv)
r
Cell types (% of CD45+)
s
Cell types (% of CD45+)
CD4conv Treg cells CD8 B cells NK cells
t
Cell types (count)
u
Cell types (count)

Supplementary Figure 16: Gating strategy for analyzing Treg cells from the brain. Representative gating strategy used to quantify Treg cell numbers in the brain using flow cytometry.

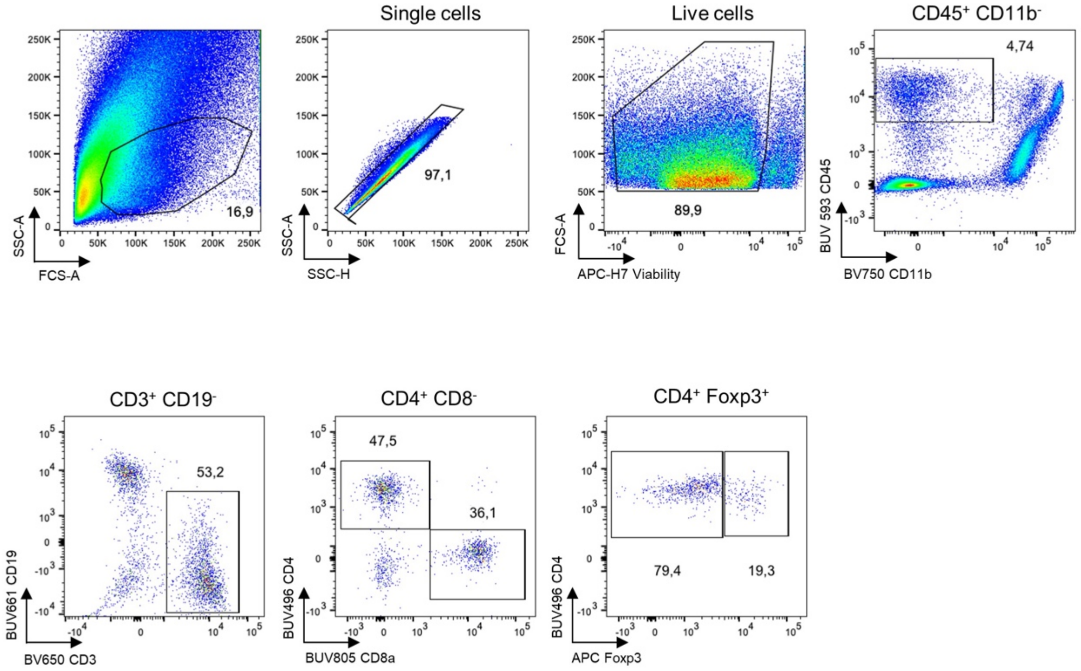

Source data for supplementary figures

Supplementary Figure 4a

	CONTROL					αCamKII IL-2		
CTX	23,9	23,3	10,5	11,1	17,1	70,6	65,6	57,7
HIPP	26,9	26,7	17,9	38,6	15,6	74,7	72,7	64,7
Mb	16,9	22,4	38,5	16,2	16,8	28,6	79,8	75
Crb	25,5	8	13,6	13,3		6,25	45,5	30,8
Ob	26,1	24,1	30,2	30	20	18,7	68,5	4,05
Men		7,94	5,88	7,64	12,2	9,3	14,8	10,4
Sc	27,6	5,88		22,7	3,9	29	17,7	33,3

Supplementary Figure 4b

	CONTROL					αCamKII IL-2		
CTX	37	24	16	8	22	6419	364	861
HIPP	18	13	25	17	16	260	2122	258
Mb	44	22	10	12	21	61	2845	433
Crb	25	2	11	4	1	1	68	12
Ob	18	7	15	3	5	21	386	28
Men	0	5	3	12	6	5	18	5
Sc	8	1	0	5	3	9	30	4

Supplementary Figure 7h

	Control							αCamKII IL-2										
CD80	1,78	1,57	1,34	1,57	0,38	2,35	3,41	2,96	4,69	1,67	4,96	3,35	2,61	2,46	4,34	1,09	1,04
IL1B	0,76	1,38	2,48	0,37	0,03	0,63	2,45	1,26	1,52	2,24	0,12	0,86	0,9	1,56	0,96	0,73	0,87
KI67	4,71	3,41	1,97	3,8	3,3	13,1	7,65	2,37	3,84	3,41	7,57	5,44	6,65	5,03	6,61	3,77	2,08
LAMP1	0,47	1,39	1,25	1,49	1,12	1,64	1,24	1,19	1,25	1,1	4,12	1,39	2,21	3,45	3,09	1,31	1,53
MHCII	0,08	0,77	0,26	0,61	1,15	3,14	13,2	6,27	7,42	0,28	44,3	12,9	19,3	22,5	31,2	8,96	12
PDL1	0,39	0,51	0,52	0,57	0,5	1,13	4,43	1,98	1,78	0,53	12	3,16	12,2	16,4	15	6,18	5,52
TGFB	1,61	0,72	0,84	0,62	0,92	0,75	1,32	0,92	1,3	0,89	0,62	1,13	0,69	0,8	0,61	0,48	0,93
TNF	0,28	0,37	0,75	0,7	3,59	10,9	0,48	0,41	1,12	0,52	1,87	3,03	8,88	12,6	13,3	10,9	3,5

Supplementary Figure 8

hours	IPSI			Contra		
24	1,35E+10	8,49E+09	1,54E+10	7E+09	4,37E+09	5,09E+09
48	1,3E+10	1,87E+10	1,68E+10	4,4E+09	6,67E+09	5,35E+09
72	1,71E+10			8,34E+09		
168	9,13E+09	1,68E+09	1,63E+10	1,53E+09	4,62E+09	7,25E+09

Supplementary Figure 9 a

	CONTROL				αCamKII IL-2			
CD4	496	372	1676	88	152	254	1160	298
Tregs	328	164	1114	68	474	284	1056	332
CD8	2276	786	3272	470	1254	700	3350	1032
TCRγδ	518	746	3008	64	1634	456	2164	488

Supplementary Figure 9 b

	CONTROL				αCamKII IL-2			
CD25	5,65	2,69	7,4	2,27	39,5	25,2	23,8	13,4
CD44	97,6	96,8	98,2	93,2	94,7	100	97,6	96,6
CD69	43,1	43,5	42,8	47,7	56,6	62,2	56	48,3
KI67	17,7	17,7	27,7	13,6	18,4	27,6	15,5	12,8
PDL1	35,5	25,8	25,9	20,5	23,7	26,8	22,6	23,5

Supplementary Figure 9 c

	CONTROL				αCamKII IL-2			
Areg	3,04	2,41	9,2	29,5	8,66	8,9	11	7,87
IL10	25	30,8	23,8	8,33	25,1	28	30,7	10,5
IL17	1,14	0,8	6,13	0,64	2,6	1,93	2,33	2,62

Supplementary Figure 9 d

	CONTROL				αCamKII IL-2			
CD25	74,4	47,6	83,8	58,8	95,4	87,3	94,3	86,1
CD44	100	97,6	99,8	94,1	100	98,6	99,6	100
CD69	68,3	67,1	63,2	47,1	91,1	88,7	86	74,7
KI67	39	40,2	40,2	32,4	27	28,9	28	29,5
PDL1	46,3	32,9	33,8	50	33,3	32,4	24,6	33,1

Supplementary Figure 9 e

	CONTROL				αCamKII IL-2			
Areg	8,37	8,41	24,8	35,6	38	33,3	22,6	29,7
IL10	13,4	18	17,2	2,96	11,5	28,1	24,6	6,7
IL17	3,59	5,22	14,1	4,44	8,17	3,82	3,41	7,18

Supplementary Figure 11a

	PHP.GFAP-GFP			PHP.GFAP-IL2				
NK	13,1	4,7	21,7	18,5	26,4	22,1	13,4	7,17
CD8	11	9,79	11	13,8	8,92	10,7	8,87	10,8
CD4	18,5	24,1	21	16,4	21,3	22,5	19,3	32,2
Treg cells	9,82	8,88	10,3	12,5	6,53	8,06	7,41	9,91

Supplementary Figure 11b

	PHP.GFAP-GFP			PHP.GFAP-IL2				
NK	44	53	36	11	7	4	16	26
CD8	407	195	699	812	438	710	769	225
CD4	359	262	347	692	573	451	597	236
Treg cells	33	15	25	116	236	171	143	19

Supplementary Figure 11c

	Cluster-01	Cluster-02	Cluster-03	Cluster-04	Cluster-05
PHP.GFAP-GFP	47,0	18,4	16,2	15,2	3,2
	50,4	17,2	12,8	11,2	8,4
	47,6	22,0	16,9	8,9	4,5
PHP.GFAP-IL2	58,1	12,1	14,6	8,2	7,1
	36,4	17,2	21,5	18,5	6,4
	43,5	17,3	21,2	13,5	4,6
	40,1	22,2	21,6	10,8	5,3
	36,6	23,9	18,8	14,1	6,6

Supplementary Figure 11d

	PHP.GFAP-GFP			PHP.GFAP-IL2				
CD25	1,61	1,59	0,95	7,59	13,4	7,84	8,71	2,73
CD44	51	40,2	46,7	34,2	55,5	52,5	56,2	55,9
CD62L	46,5	46,6	46,4	55,5	36,3	41,2	38,1	31,8
CD69	42,3	27,9	35,3	54,8	53,1	50,2	40,7	39,1
CD103	1,29	3,19	0,63	1,08	1,03	2,75	1,65	0
CTLA4	1,29	1,2	0,95	1,08	1,37	0,39	4,24	2,73
ICOS	19	7,97	9,78	7,05	18,2	10,2	12,7	10
Ki67	10	10,8	10,7	7,78	13,7	11	8	10
KLRG1	2,26	1,99	3,79	0,54	1,03	0,78	2,59	1,82
Neuropilin1	4,52	1,2	3,47	3,98	10,3	8,24	6,82	5
PD1	13,5	9,96	14,2	13	25,7	21,6	18,4	20,5
ST2	1,61	0,4	0,95	0,9	3,77	0,78	1,65	0,91

Supplementary Figure 11e

	Cluster-01	Cluster-02	Cluster-03	Cluster-04	Cluster-05	Cluster-06
PHP.GFAP-GFP	52,8	34,0	2,8	4,2	0,2	5,9
	44,2	46,5	0,0	9,3	0,0	0,0
	64,9	27,6	3,0	3,9	0,0	0,7
PHP.GFAP-IL2	37,7	46,7	8,3	5,5	0,7	1,1
	39,5	11,0	43,1	1,6	4,2	0,7
	43,1	17,6	34,7	2,2	2,2	0,3
	46,0	36,6	11,1	4,2	0,4	1,8
	42,8	32,7	16,4	5,7	0,6	1,9

Supplementary Figure 11f

	PHP.GFAP-GFP			PHP.GFAP-IL2				
CD25	3,14	1,5	1,79	12,7	39,2	30,6	15,6	6,29
CD44	69,1	55,6	73,8	55,9	63,9	63,5	70,6	64,8
CD62L	35,5	42,9	26,9	45	10,7	17,9	36,2	32,1
CD69	55,8	40,6	69	73,8	90,4	86,8	62,3	59,7
CD103	9,18	0,75	0,45	4,04	10,1	10,7	5,98	4,4
CTLA4	0,24	0	0,74	0,49	0,17	0,14	0,91	1,26
ICOS	8,45	0,75	2,23	1,23	1,44	1,57	3,51	3,14
Ki67	19,3	5,26	10,7	8,33	11,1	12,9	8,06	6,29
KLRG1	21,7	37,6	19,6	34,2	7,29	11,4	21,5	25,8
Neuropilin1	6,28	0,75	1,34	2,82	1,44	1,86	1,04	1,26
PD1	41,8	20,3	58	35	79,2	69,1	47,3	34,6
ST2	0,72	0	1,19	4,53	21	15,6	4,16	6,29

Supplementary Figure 11g

	Cluster-01	Cluster-02	Cluster-03	Cluster-04	Cluster-05
PHP.GFAP-GFP	75,2	4,2	15,2	5,4	0,0
	73,0	3,9	22,8	0,0	0,3
	76,2	4,1	18,6	0,5	0,5
PHP.GFAP-IL2	70,9	16,2	10,6	1,0	1,4
	57,6	34,0	6,3	0,5	1,6
	64,1	27,3	6,2	0,8	1,6
	63,2	16,0	19,3	0,9	0,6
	77,4	10,5	8,9	1,6	1,6

Supplementary Figure 11h

	PHP.GFAP-GFP			PHP.GFAP-IL2				
CD25	33,3	15	23,6	26,4	43,9	44,2	28,4	15,5
CD44	96,2	85	90	94,9	94,4	95,4	94,9	72
CD62L	8,55	11,6	6,02	5,11	2,2	2,95	6,86	6,15
CD69	68,5	49,6	61,3	84,7	85,1	89,7	67	57,1
CD103	1,54	10,7	2,93	3,87	8,6	8,29	4,51	16,6
CTLA4	0	0,29	0	0,14	0,42	0	0,45	1,68
ICOS	26,8	14,4	18,1	23,2	47,1	45,1	23	16,2
Ki67	12,1	4,41	9,1	10,6	10,6	12,2	10,1	5,17
KLRG1	10,1	4,41	5,86	8,84	15,3	12,6	11,1	7,97
Neuropilin1	33	10,6	21,3	34	24,5	22,5	30,3	7,13
PD1	52,6	16,3	31,9	55,7	59	56,5	50,1	19,9
ST2	17,9	11	13,3	7,6	12,9	12,4	15,7	14

Supplementary Figure 11i

	Cluster-01	Cluster-02	Cluster-03	Cluster-04	Cluster-05
PHP.GFAP-GFP	71,1	0,0	2,6	10,5	15,8
	77,8	5,6	0,0	5,6	11,1
	66,7	7,4	11,1	11,1	3,7
PHP.GFAP-IL2	50,7	12,7	18,3	11,3	7,0
	52,1	20,2	19,3	6,7	1,7
	44,4	31,3	16,2	4,0	4,0
	61,8	21,3	11,2	1,1	4,5
	62,9	11,4	2,9	17,1	5,7

Supplementary Figure 11j

	PHP.GFAP-GFP			PHP.GFAP-IL2				
CD25	64,5	55	55,6	67,4	85,1	90,3	61,8	69,2
CD44	61,3	55	77,8	76,7	85,1	84,7	79,4	84,6
CD62L	29	15	16,7	25,6	9,46	15,3	8,82	23,1
CD69	38,7	40	66,7	72,1	83,8	77,8	60,3	53,8
CD103	12,9	35	16,7	14	23	33,3	20,6	7,69
CTLA4	51,6	70	61,1	67,4	67,6	66,7	66,2	61,5
ICOS	48,4	40	33,3	48,8	58,1	54,2	48,5	61,5
KI67	25,8	20	33,3	34,9	21,6	33,3	25	23,1
KLRG1	16,1	15	22,2	14	13,5	22,2	11,8	23,1
Neuropilin1	90,3	65	66,7	83,7	71,6	73,6	79,4	69,2
PD1	64,5	60	66,7	81,4	90,5	86,1	83,8	76,9
ST2	22,6	20	16,7	11,6	12,2	23,6	17,6	7,69

Supplementary Figure 12a

	PHP.GFAP-GFP Sham			PHP.GFAP-GFP				PHP.GFAP-IL-2			
microglia	16638	32733	18678	46925	49249	60178	35636	24115	34991	13132	4916

Supplementary Figure 12b

	PHP.GFAP-GFP Sham			PHP.GFAP-GFP				PHP.GFAP-IL-2			
CD8	20,3	18,6	9,49	16,7	18	16,5	12,8	13,7	14,4	25	17,1
CD4	4,88	5,82	11	15,2	9,05	13,8	9,03	8,55	7,69	10,4	13,7

Supplementary Figure 12c

	PHP.GFAP-GFP Sham			PHP.GFAP-GFP				PHP.GFAP-IL-2			
Treg cells	16	44	112	147	97	337	57	101	61	102	321
CD4	133	316	1314	707	536	1010	471	292	278	249	1067
CD8	624	1165	1252	961	1275	1621	757	632	642	855	1746

Supplementary Figure 12d

	PHP.GFAP-GFP Sham			PHP.GFAP-GFP				PHP.GFAP-IL-2			
CD25	0	2,22	0,84	1,98	0,93	3,66	2,12	2,05	3,6	2,01	2,16
CD44	94	88,6	76,3	96,2	90,7	97,9	81,3	72,3	96,8	97,6	98,7
CD69	10,5	9,18	4,11	17	14,2	17,3	7,86	12,3	19,1	14,5	22,9
KI67	9,77	10,1	7	12,7	13,4	17,8	14,9	9,25	9,71	19,3	24,4
PDL1	71,4	63	40,7	80,6	75	68,3	65,4	64,4	74,5	69,1	59,2
ST2	0	0	0,076	0,14	0	0,2	0,42	0	1,08	0,4	0,37

Supplementary Figure 12e

	PHP.GFAP-GFP Sham			PHP.GFAP-GFP				PHP.GFAP-IL-2			
Areg	0,99	1,27	0,53	0,24	1,15	0,34	0,54	1,01	1,31	1,46	1,53
IL10	2,03	4,11	7,06	9,36	6,38	10,4	9,76	5,39	2,79	2,03	4,37

Supplementary Figure 12f

	PHP.GFAP-GFP Sham			PHP.GFAP-GFP				PHP.GFAP-IL-2			
Areg	1329	1700	1718	1724	1757	1803	1831	1622	1517	1454	1706

Supplementary Figure 12g

	PHP.GFAP-GFP Sham			PHP.GFAP-GFP				PHP.GFAP-IL-2			
CD25	87,5	77,3	s	75,5	61,9	71,2	73,7	74,3	75,4	63,7	72,6
CD44	100	97,7	90,2	98	99	98,5	93	96	98,4	99	99,4
CD69	18,8	20,5	11,6	43,5	22,7	32	28,1	36,6	31,1	20,6	33,6
KI67	25	36,4	28,6	25,2	35,1	37,7	21,1	21,8	31,1	61,8	49,5
PDL1	68,8	77,3	67	84,4	91,8	78,3	80,7	81,2	77	86,3	75,4
ST2	0	0	0,89	0,68	1,03	0,89	0	0	1,64	1,96	1,25

Supplementary Figure 12h

	PHP.GFAP-GFP Sham			PHP.GFAP-GFP				PHP.GFAP-IL-2			
Areg	26,3	22,2	21,4	4,11	7,69	16,5	7,58	20,9	21,9	34,7	22,8
IL10	0	0	0	2,74	1,1	0,79	1,52	0	0	1,33	1,63

Supplementary Figure 12i

Areg	PHP.GFAP-GFP Sham			PHP.GFAP-GFP				PHP.GFAP-IL-2			
	2220	2095	1934	1548	1594	1928	1631	1997	2237	2360	2007

Supplementary Figure 12l

	PHP.GFAP-GFP					PHP.GFAP-IL-2				
CD4	35,5	49,1	34,5	20	33,7	41,3	41,3	54,4	45	41,8
CD8	33,2	30,7	35,8	18,5	32	34	41	37,7	42,2	35,9
TCRγδ	1,35	2	1,64	0,96	0,34	0,98	0,49	0,68	0,83	2,58

Supplementary Figure 12m

	PHP.GFAP-GFP					PHP.GFAP-IL-2				
Treg cells	12	5,33	8,94	20,7	21,7	12,1	18	6	15,6	14,4

Supplementary Figure 12n

	PHP.GFAP-GFP					PHP.GFAP-IL-2				
CD4 conv	786722	82768	134952	26294	1443129	26285	140266	1174484	87318	26940
CD8	735202	51771	140053	24220	1371643	21673	139179	814580	81998	23155
TCRγδ	44212	6095	11089	2579	32553	956	3040	22184	2899	3211
B cells	690306	92823	116737	36274	3340264	21626	158299	594021	92936	38126

Supplementary Figure 12o

	PHP.GFAP-GFP					PHP.GFAP-IL-2				
CD4	48,1	49,2	46,2	43,5	45,3	46,8	45,1	41,8	43,6	45,4
CD8	40,6	43,7	43,3	48,6	47,1	41,4	47,5	47,4	44,4	42,9
TCRγδ	0,27	0,51	0,34	0,34	0,36	0,24	0,42	0,42	0,29	1,11

Supplementary Figure 12p

	PHP.GFAP-GFP					PHP.GFAP-IL-2				
Treg cells	12,1	14,4	13,6	12,6	12,6	14,6	12,9	14,2	13,9	14,7

Supplementary Figure 12q

	PHP.GFAP-GFP					PHP.GFAP-IL-2				
CD4 conv	2362432	1688499	1578027	1905000	1254611	2470943	1745558	1156024	1491065	820810
Treg cells	35760	34656	26807	34371	19838	72272	37664	32803	29520	15113
CD8	1995264	1500750	1478933	2130480	1301820	2187077	1840378	1312679	1517705	776466
TCRγδ	30912	45411	32267	35880	27387	32089	40551	29093	28135	38663
B cells	3370688	2812809	3053600	2802840	2482554	3427560	2678891	2111279	2397822	1047353

		PHP.GFAP-GFP Sham		PHP.GFAP-GFP			PHP.GFAP-IL-2				
**Supplementary Figure 13a**		PHP.GFAP-GFP Sham		PHP.GFAP-GFP			PHP.GFAP-IL-2				
	microglia	64826	62716	71752	71172	99994	91528	45580	57840	42158	57304
**Supplementary Figure 13b**		PHP.GFAP-GFP Sham		PHP.GFAP-GFP			PHP.GFAP-IL-2				
	CD8	3,26	5,33	10,7	8,49	9,19	10,6	18,7	15,5	9,37	6,11
	CD4	3,35	5,16	13,8	9,32	11	14,3	14,1	12,3	15,9	8,36
	TCRγδ	1,3	1,23	1,25	1,29	1,27	2,12	0,48	2,32	1,56	1,17
**Supplementary Figure 13c**		PHP.GFAP-GFP Sham		PHP.GFAP-GFP			PHP.GFAP-IL-2				
	Tregs	10,5	9,43	19,7	27,2	14,8	21,3	7,07	19,4	28,6	15,8
**Supplementary Figure 13d**		PHP.GFAP-GFP Sham		PHP.GFAP-GFP			PHP.GFAP-IL-2				
	CD4	568	1020	2344	1268	1998	2486	31016	4016	1624	1196
	Treg cells	70	110	598	502	360	712	2246	1014	696	236
	CD8	686	1130	3894	2026	3034	4518	25402	4134	4134	2038
	TCRγδ	184	182	352	194	352	544	864	648	406	286
**Supplementary Figure 13e**		PHP.GFAP-GFP Sham		PHP.GFAP-GFP			PHP.GFAP-IL-2				
	CD25	2,16	1,57	2,3	3,48	1,2	1,37	1,28	2,04	2,34	1,24
	CD44	45,4	46,5	74,9	74,9	43,3	82,9	13,4	55,4	69,4	58,9
	CD69	7,04	5,88	15,2	11,1	5,61	7,96	2,28	3,93	4,56	5,52
	Ki67	14,4	18	31,5	30,5	14,4	18,8	4,67	18	21,3	17,1
	PDL1	13,4	16,1	19	24,5	11,2	18,4	6,51	12,5	21,3	19,9
**Supplementary Figure 13f**		PHP.GFAP-GFP Sham		PHP.GFAP-GFP			PHP.GFAP-IL-2				
	Areg	1,69	6,52	11,8	14	6,67	17,3	0,4	21,9	32,4	0
	IL10	5,08	6,52	5,88	14	11,7	17,3	1,44	30,5	31	6,06
	IL17	3,39	10,9	9,8	10,5	6,67	7,69	0,94	11,7	8,45	6,06
**Supplementary Figure 13g**		PHP.GFAP-GFP Sham		PHP.GFAP-GFP			PHP.GFAP-IL-2				
	CD25	45,7	29,1	49,5	51,4	38,9	30,1	33,6	39,4	37,9	38,1
	CD44	68,6	67,3	75,6	80,1	69,4	78,4	47,2	75	77,6	68,6
	CD69	11,4	21,8	26,4	13,9	15,6	11,5	11,4	9,47	7,18	21,2
	Ki67	22,9	38,2	39,8	47,8	38,9	33,4	31,8	43,8	40,2	37,3
	PDL1	28,6	16,4	22,7	29,9	13,9	17,7	15,3	16	25	33,1
	ST2	8,57	5,45	6,35	6,37	5,56	7,87	4,97	11,8	7,76	3,39

Supplementary Figure 13h

	PHP.GFAP-GFP Sham		PHP.GFAP-GFP			PHP.GFAP-IL-2				
Areg	22,2	0	22,2	30	27,3	50	10,2	31,8	50	60
IL10	5,56	22,2	0	60	18,2	33,3	18,4	50	50	50
IL17	0	11,1	0	10	0	0	2,04	4,55	0	20

Supplementary Figure 13i

	PHP.GFAP-GFP Sham		PHP.GFAP-GFP			PHP.GFAP-IL-2				
CD4conv	23	18,8	25,2	20,6	19,9	17,3	16,7	21,2	18,3	22,5
Treg cells	2,39	1,81	3,76	3,46	3,29	2,88	1,64	3,55	2,03	2,25
CD8	21,6	19,8	24,1	20,4	18,6	14,7	13,3	20,4	14,7	19
B cells	41,8	48	38	37,2	50,2	47,3	36,7	43,1	50,1	41,7
TCRγδ	0,49	0,44	0,47	0,4	0,5	0,28	0,24	0,58	0,29	0,41

Supplementary Figure 13j

	PHP.GFAP-GFP Sham		PHP.GFAP-GFP			PHP.GFAP-IL-2				
CD4conv	1364833	984986	1776059	272585	1080076	883947	477000	1385400	762554	972993
Treg cells	141797	94661	264941	45722	178297	147001	46682	231967	84562	97475
CD8	1278433	1035996	1699725	270026	1009276	753153	379280	1332233	612819	824764
B cells	2479495	2508686	2680804	491773	2723952	2418644	1047075	2820700	2087727	1805548
TCRγδ	29193	22821	32922	5287	27166	14553	6748	37733	12129	17793

		PHP.GFAP-GFP Sham			PHP.GFAP-GFP						PHP.GFAP-IL-2					
**Supplementary Figure 14a**		PHP.GFAP-GFP Sham			PHP.GFAP-GFP						PHP.GFAP-IL-2					
	microglia	66730	56802	35386	72700	90926	1E+05	47608	93190	30324	1E+05	1E+05	60612	59764	1E+05	83284
**Supplementary Figure 14b**		PHP.GFAP-GFP Sham			PHP.GFAP-GFP						PHP.GFAP-IL-2					
	CD8	6,25	8,21	10,38	14,6	11,1	20,2	12,8	12,6	15,6	20,3	12,9	16,7	15,5	13,2	20,2
	CD4	4,04	6,02	7,08	11,6	11,8	12,4	9,28	12,5	11,8	12,5	16,3	10,8	13,3	13,4	15,9
	TCRγδ	1,28	2,23	2,3	4	8,22	3,35	4,15	6,06	4,53	4,13	4,08	3,5	7,25	9,19	5,02
**Supplementary Figure 14c**		PHP.GFAP-GFP Sham			PHP.GFAP-GFP						PHP.GFAP-IL-2					
	Treg cells	8,09	6,25	6,13	27,5	24,4	25,9	25,3	38,9	31,7	28	25,8	24,1	25,5	33,8	29,2
**Supplementary Figure 14d**		PHP.GFAP-GFP Sham			PHP.GFAP-GFP						PHP.GFAP-IL-2					
	CD4	672	408	430	2472	2432	3992	1448	1886	890	3918	2872	1400	2484	2650	2550
	Treg cells	58	28	32	984	834	1458	516	1272	438	1584	1052	466	870	1460	1100
	CD8	1166	582	736	4510	3194	9174	2936	3294	1826	9136	3220	2974	3966	4266	4786
	TCRγδ	238	136	140	1112	2374	1522	952	1588	530	1860	920	624	1860	2836	1094
**Supplementary Figure 14e**		PHP.GFAP-GFP Sham			PHP.GFAP-GFP						PHP.GFAP-IL-2					
	CD25	0	0,49	0	1,29	1,39	1,5	1,23	2,55	1,07	1,28	2,09	1,29	1,05	2,29	1,1
	CD44	53,6	59,8	54	82,9	85,1	85,6	83,1	84	77,3	86,2	81,4	81,4	90,3	82,6	82,2
	CD69	33,9	24,5	30,7	32,9	37,2	36,1	27,6	27,3	33,3	30,6	38	33,7	32,5	21,8	30,7
	Ki67	14,9	9,31	7,44	15,6	16,9	12,6	17,8	20,3	13,5	22,7	24,7	15,6	34,1	18,9	19
**Supplementary Figure 14f**		PHP.GFAP-GFP Sham			PHP.GFAP-GFP						PHP.GFAP-IL-2					
	Areg	29,1	24,1	32,2	67,5	63	61,7	68,6	63,6	59,6	57,8	63,3	57,6	65,2	60,4	35,7
	IL10	22,3	20,2	23	21,3	21,2	29,5	31,5	35,9	33,7	36,3	31,4	30,8	36,3	36,5	32
	IL17	18,2	15	13,1	6,56	8,92	6,66	7,67	11,2	9,06	10,7	8,19	8,48	11,1	11,5	22,6
**Supplementary Figure 14g**		PHP.GFAP-GFP Sham			PHP.GFAP-GFP						PHP.GFAP-IL-2					
	CD25	20,7	35,7	43,8	42,3	42,7	49	51,9	63,7	42	59,5	58,7	62,7	39,8	42,9	44,5
	CD44	75,9	42,9	75	83,5	80,8	81,3	89,1	89,6	81,7	86,4	83,3	73,8	90,6	85,6	76,2
	CD69	31	35,7	18,8	44,3	35,3	43,5	30,6	32,2	42,9	36,1	41,1	47,6	29,2	27,7	30
	Ki67	58,6	28,6	43,8	47,2	50,8	53,6	57,4	66,2	51,6	64,8	58,4	47,2	77,2	61,4	61,1
**Supplementary Figure 14h**		PHP.GFAP-GFP Sham			PHP.GFAP-GFP						PHP.GFAP-IL-2					
	Areg	39,4	26,7	28,6	34,1	14,5	11,9	30	7,95	9,96	10,9	16,9	16,1	23,1	17,1	20,5
	IL10	9,09	13,3	14,3	17,3	4,71	7,29	7,59	9,17	3,98	8,03	10,5	14	19,2	10,5	16,9
	IL17	15,2	10	17,9	6,73	7,74	7,17	7,59	6,85	3,59	5,81	5,83	6,61	17,4	10,1	17,1

**Supplementary Figure 15a**					PHP.GFAP-GFP										PHP.GFAP-IL-2					
D0	35,1	34,7	27,8	33,6	34,6															
D15	17,1	39,7	14,2	15,9	18,5						39,6	24,1	39,1	28,1	17,6					
D21	25,4	26,4	29	19	33,1	23,7	33,3	41,9	29	25,6	32,7	30,7	24,4	29	10,7	25,1	19,3	32,8	29,4	24
D30	31,7	45,2	31,7	38,6	34,6	28,4	22,1				30,4	25,8	27,1	47,6	40,6	38,3				

**Supplementary Figure 15b**					PHP.GFAP-GFP										PHP.GFAP-IL-2					
D0	29,6	28	34,7	16,9	25,3															
D15	62,7	67	79,7	77,2	68,2						80,7	65	79,8	77,9	78,2					
D21	73,8	72,1	63,9	67,3	64,9	80,1	76,5	83,1	65	62,1	82,1	66,2	70,7	58,1	77,2	68,6	59,1	78,5	69,2	68
D30	66,8	69,8	72,8	63,7	82,6	67,7	72				28	77,7	81,2	82,6	83,6	74,5				

**Supplementary Figure 15c**					PHP.GFAP-GFP										PHP.GFAP-IL-2					
D0	10,6	12,6	10,5	7,43	10,1															
D15	30,8	27,9	14,1	15,9	15,1						12,3	28,2	14,7	14,1	15,6					
D21	20,7	23,3	30,1	24,9	28,3	17,4	16,7	13,6	29,6	33,3	13,1	28,6	23,3	33,6	16,6	28,6	37,9	18,5	25,3	24
D30	28,2	24,8	23,3	29	12,7	27	23,7				55,5	21,3	17,6	16,2	15,1	23,7				

**Supplementary Figure 15d**					PHP.GFAP-GFP										PHP.GFAP-IL-2					
D0	131	102	195	236	121															
D15	11238	28336	8580	8622	6274						21080	17924	1218	31062	10108					
D21	8828	190	346	1916	3258	1790	884	4272	4786	2348	19416	9358	3202	356	20	410	6304	1038	6910	1032
D30	16514	14088	13792	4590	3190	4280	9114				684	4900	5742	1618	930	4320				

**Supplementary Figure 15e**					PHP.GFAP-GFP										PHP.GFAP-IL-2					
D0	161	97	340	247	124															
D15	32540	26516	38084	33404	19964						24416	35434	1236	59868	31382					
D21	19386	202	294	1778	5444	572	486	3470	11382	2742	28816	16636	3018	476	34	1280	4586	366	2476	862
D30	17998	7296	17790	3690	2760	5978	19318				254	8870	9912	816	986	4244				

**Supplementary Figure 15f**					PHP.GFAP-GFP										PHP.GFAP-IL-2					
D0	19	14	40	20	14															
D15	14962	10580	6378	6544	3742						3518	14336	220	10058	5996					
D21	3370	1372	5344	11696	2374	4214	44	48	810	2920	476	7040	1580	7872	5782	12010	10666	710	174	12
D30	7588	2592	5686	1678	426	2384	6362				504	2428	2154	160	178	1350				

Supplementary Figure 15g

	PHP.GFAP-GFP						PHP.GFAP-IL-2				
CD25	39,8	42,1	38,2	23,4	34,4	35,58	29,2	36,5	30,7	32,2	31,4
CD44	38,2	33,7	28,3	24,3	32,4	31,38	31,2	33,4	33,9	40,3	41,2
CD62L	22,1	23,9	32,4	31,3	18,5	25,64	16,8	15,2	17,9	18,8	17,3
CD69	37,2	39,6	27,8	29,3	42,9	35,36	38,9	48,1	38,8	39,4	41,6
CD103	9,76	8,49	5,93	5,7	8,08	7,592	7,53	11,1	8,91	7,81	9,02
CTLA4	53,7	61,8	55,1	51,4	69,1	58,22	56,9	73,1	60,9	52,8	53,7
GITR	58,1	58,9	55,7	49,4	70,3	58,48	63,4	79,5	74,4	57,4	63,9
Helios	84,8	86,7	57,4	89,7	88,9	81,5	89,1	89,4	84,3	86,6	88,1
ICOS	70,6	68,7	44,2	45,4	67,9	59,36	66,3	83,2	67,4	66,7	64,9
KI67	34,6	38,2	34,1	24,9	30,5	32,46	38,3	37,1	41,1	37,6	36,7
KLRG1	20,9	24,4	12,8	5,6	10,5	14,84	12,1	17,4	12	10,1	14,3
Neuropilin1	68,2	57,3	54,7	38,1	50,1	53,68	50,2	70,7	55,5	53	58,1
PD1		43,1	35	28,9	38,7	36,425	40,6	55,2	42,7	44,6	40,4
Tbet	15,6	9,2	1,16	19,8	13,9	11,932	11,8	9,45	9,61	13,2	13,4

Supplementary Figure 15h

							PHP.GFAP-IL-2				
CD25	62,6	63,6	50	41,7	42,8	52,14	53,8	58,2	49,3	41,4	83,3
CD44	50	40,9	62,5	68,4	65,7	57,5	53,6	60	57,5	65,5	50
CD62L	3,99	4,55	0	1,48	1,1	2,224	2,85	1,91	3,1	4,6	0
CD69	41,2	36,4	45,8	18,8	17,2	31,88	25,9	28	29,9	35,6	50
CD103	22,6	27,3	25	14,8	26,8	23,3	32,1	38,1	23,4	32,2	33,3
CTLA4	83,3	86,4	54,2	58	75,2	71,42	82,8	76,7	74,9	71,3	100
GITR	95,7	90,9	87,5	88,1	88,9	90,22	92,3	93,2	86,5	83,9	100
Helios	77,1	63,6	83,3	77,8	74,5	75,26	71,8	74,5	72,7	82,8	50
ICOS	76,5	77,3	62,5	59,8	80,6	71,34	82	80,3	69,3	72,4	83,3
KI67	46,9	50	58,3	39	61,3	51,1	45,5	46,4	40,3	51,7	33,3
KLRG1	22,6	36,4	33,3	24,7	27,3	28,86	27,9	30,2	31,3	14,9	33,3
Neuropilin1	53,7	54,5	33,3	74,6	81,3	59,48	73,1	64,1	55,2	50,6	50
PD1	22	22,7	8,33	39	30,3	24,466	24,9	23,9	22,3	11,5	33,3
Tbet	7,4	13,6	20,8	11,4	10,9	12,82	5,13	8,83	12,4	21,8	0

Supplementary Figure 15i

	PHP.GFAP-GFP							PHP.GFAP-IL-2					
CD25	55,1	55,2	49,9	51	48,4	68,6	53,3	73,4	61,4	64,6	71,2	42,7	57,5
CD44	47,7	37,8	37,4	28,4	24,9	44,6	50,4	41,7	45	57,3	31,2	13,5	24,3
CD62L	0,9	1,39	2,85	0,83	2,35	0,92	3,11	1,98	2,31	0,56	5	4,49	3,26
CD69	60,1	59,2	50,9	46,4	39	49,6	41,7	45,6	50,2	70,7	55	37,1	53
CD103	57,4	47,4	53,9	37,8	35,7	55,5	47,6	42,1	37,5	43,9	37,5	39,3	60
CTLA4	66,6	47,9	63,4	42,9	50,2	75,4	76,7	61,5	58,8	59,1	63,7	46,1	65,9
GITR	86,1	83,6	86,8	76,6	78,9	87,8	87,5	77,8	84,7	78,6	77,5	70,8	79
Helios	79,4	82,8	74,7	86,1	76,5	78	78,2	91,3	87,4	84,7	86,2	69,7	65
ICOS	68,2	64,6	72,1	47	60,1	75,3	72,5	77	69,1	61,8	62,5	59,6	83,7
KI67	43	27,5	41,5	22,9	27,2	42,4	51,6	54,8	37,6	29,1	48,8	24,7	37,5
KLRG1	29	27,9	29,4	33,1	38,5	27,8	29,9	22,6	31,2	30,4	31,2	23,6	23,3
Neuropilin1	92,8	90,6	87,4	87,4	83,1	94	94,8	88,1	90,6	88,3	86,2	74,2	80,7
PD1	8,41	11,7	5,31	9,18	15	16,1	5,72	29,4	12,1	9,47	16,2	10,1	11,1

Supplementary Figure 15j	PHP.GFAP-GFP							PHP.GFAP-IL-2				
D15	0,69	0,84	0,83	1,01	1,46			1,01	1,79	0,48	0,99	1,38
D21	4,15	2,7	2,04	1,03	0,56			7,21	7,49	9,36	5,77	5,78
D30	1,47	4,25	0,84	1,25	0	0,22	0,083	1,08	0,13	0	0,45	0

Supplementary Figure 15k	PHP.GFAP-GFP					PHP.GFAP-IL-2				
D15	0,48	0,42	0,6	0,2	0,24	0,41	0,27	0,78	0,76	1,92
D21	4,15	2,7	2,04	1,03	0,56	10,5	3,2	4,07	3,22	5,76

Supplementary Figure 15l	PHP.GFAP-GFP							PHP.GFAP-IL-2				
D15	12,1	6,41	13,5	3,9	2,66			6,18	7,61	7,74	11,5	9,52
D21	29,3	32,1	26	36	44,4			37,7	37,3	23,1	22,4	21
D30	9,59	8,79	6,68	3,51	31	10,7	15,4	10,8	11,6	9,3	10,8	12,2

Supplementary Figure 15m	PHP.GFAP-GFP					PHP.GFAP-IL-2				
D15	19,56	23,82	29,91	21,36	13,71	41,49	12,46	32,01	30,86	25,76
D21	53,87	55,06	48,62	46,19	32,4	41,86	46,34	47,35	54,18	44,53

Supplementary Figure 15n	PHP.GFAP-GFP							PHP.GFAP-IL-2				
D15	35,3	44,8	56,4	42,2	32,6			57,3	35	53,6	55,3	47,2
D21	59,7	54,2	57,6	58,1	52,3			52,5	66,6	45,6	53,9	46
D30	3,65	1,73	8,42	9,52	19,7	28,6	15,4	34,4	29,5	32,3	22,1	12,2

Supplementary Figure 15o	PHP.GFAP-GFP							PHP.GFAP-IL-2				
D15	60,9	60	72,2	55,2	52,8			69,5	52,1	61,9	70	60,8
D21	92	90,5	86,7	87,1	84,9			89,9	86,9	79,9	85,7	86,8
D30	49,1	38,5	51	47,4	71,8	80,1	79,3	77,4	86,6	81,1	56,3	75,6

Supplementary Figure 15p	PHP.GFAP-GFP						PHP.GFAP-IL-2				
D15	18,3	19,3	25,6	9,92	6,7		26,2	16,2	21,2	29,1	22,4
D21	64,9	60,4	52,1	60,3	54,8		65,6	58,7	46,8	55,2	59,5
D30	48,4	55,4	52,7	70,4	56,8	74,8	52,7	41,9	57,9	53	53

Supplementary Figure 15q	PHP.GFAP-GFP					PHP.GFAP-IL-2				
D15	1,26	2,32	1,81	2,06	0,71	4,09	1,26	4,91	3,26	2,96
D21	52,7	53	45,6	42,6	30,6	40,6	44,3	42,6	51,3	42,7

Supplementary Figure 15r	PHP.GFAP-GFP					PHP.GFAP-IL-2				
CD4conv	32,2	40	31,7	39,4	33,8	34,4	34,2	32,1	33,7	36,1
Tregs	3,44	3,21	3,3	4,07	3,68	4,03	4,21	3,9	4,65	3,71
CD8	31,1	34,9	27,4	28,4	30,4	29,6	29,9	27	27,2	25,8
B cells	25,5	17,6	30,4	22,4	27,8	23	27	29,3	24,9	26,6
NK cells	0,36	0,53	0,19	0,3	0,38	0,16	0,52	0,28	0,29	0,4

Supplementary Figure 15s	PHP.GFAP-GFP					PHP.GFAP-IL-2				
CD4conv	28,1	21	30,9	39,2	29,4	31,9	36	33,9	34,2	38,2
Tregs	3,51	2,48	2,98	3,58	3,13	3,22	4,66	4,94	4,66	3,83
CD8	25,2	22,7	28,6	32,1	29,6	28,6	33,1	31,1	29,9	28,6
B cells	38,7	47,8	32,6	20	32,8	30,5	21,3	23,6	26,3	22,6
NK cells	0,54	0,63	0,45	0,84	0,73	0,42	0,42	0,48	0,51	0,52

Supplementary Figure 15t	PHP.GFAP-GFP					PHP.GFAP-IL-2				
CD4conv	667575	894063	391332	830446	875011	396821	430176	221463	413692	187857
Tregs	71223	71836	40636	85699	95243	46416	52970	26921	57017	19308
CD8	644000	781616	337276	597608	785445	340946	376340	186047	333346	134152
B cells	529613	393467	375276	471284	719277	265508	339898	202335	304746	138263
NK cells	7398	11940	2294	6370	9806	1865	6562	1902	3529	2055

Supplementary Figure 15u	PHP.GFAP-GFP					PHP.GFAP-IL-2				
CD4conv	1189200	342087	355733	1386803	370750	787850	2213933	2371017	4518683	2013600
Tregs	148560	40327	34267	126480	39400	79400	286400	345217	616733	201920
CD8	1066880	370607	328600	1134410	373150	705600	2035200	2176417	3951153	1509200
B cells	1635920	779240	375200	707143	412400	751400	1311667	1647217	3474490	1191040
NK cells	22800	10273	5133	29637	9250	10450	25867	33367	67860	27520
