# Supplementary material for: Astrocyte-targeted gene delivery of interleukin 2 specifically increases brain-resident regulatory T cell numbers and protects against pathological neuroinflammation
Source: Nat Immunol. 2022 May 26;23(6):878–91. doi: 10.1038/s41590-022-01208-z (PMC9174055; doi:10.1038/s41590-022-01208-z)
Supplement: Supplementary file 2 — Reporting Summary [file 41590_2022_1208_MOESM2_ESM.pdf]

nature portfolio

Corresponding author(s): Matthew Holt, Adrian Liston

Last updated by author(s): 25 March, 2022

Reporting Summary

Nature Portfolio wishes to improve the reproducibility of the work that we publish. This form provides structure for consistency and transparency in reporting. For further information on Nature Portfolio policies, see our Editorial Policies and the Editorial Policy Checklist.

Statistics

For all statistical analyses, confirm that the following items are present in the figure legend, table legend, main text, or Methods section.

n/a	Confirmed	
☐	☒	The exact sample size (*n*) for each experimental group/condition, given as a discrete number and unit of measurement
☐	☒	A statement on whether measurements were taken from distinct samples or whether the same sample was measured repeatedly
☐	☒	The statistical test(s) used AND whether they are one- or two-sided<br>*Only common tests should be described solely by name; describe more complex techniques in the Methods section.*
☒	☐	A description of all covariates tested
☐	☒	A description of any assumptions or corrections, such as tests of normality and adjustment for multiple comparisons
☐	☒	A full description of the statistical parameters including central tendency (e.g. means) or other basic estimates (e.g. regression coefficient) AND variation (e.g. standard deviation) or associated estimates of uncertainty (e.g. confidence intervals)
☐	☒	For null hypothesis testing, the test statistic (e.g. *F*, *t*, *r*) with confidence intervals, effect sizes, degrees of freedom and *P* value noted<br>*Give P values as exact values whenever suitable.*
☒	☐	For Bayesian analysis, information on the choice of priors and Markov chain Monte Carlo settings
☒	☐	For hierarchical and complex designs, identification of the appropriate level for tests and full reporting of outcomes
☒	☐	Estimates of effect sizes (e.g. Cohen's *d*, Pearson's *r*), indicating how they were calculated

Our web collection on statistics for biologists contains articles on many of the points above.

Software and code

Policy information about availability of computer code

Data collection	Flow cytometry data collection was performed using FACSDIVA version 8.0.2 (BD) or SpectroFlo version 3.0 (Cytek).<br>Flow sorting was performed using FACSDIVA version 8.0.1 (BD).<br>Sequencing data was collected on a Illumina Novaseq 6000 or Illumina HiSeq.<br>Behaviour data was collected with Ethovision (version 7 or 14, Noldus) for open field /morris water maze/ light dark test/ novel object recognition; and ANY-maze Video tracking system (Stoelting Europe) for sociability / forced swim test. MRI measurements were collected on 9.4 T Bruker Biospec small animal MR system.<br>Immunostainings were collected with Zen software (Zeiss), Nikon NIS (Nikon), or Zeiss Axio Examiner Z1.<br>Multi-electrode array electrophysiology recordings were done in multielectrode array (MEA 2100, Multi Channel Systems).<br>Live functional imaging in acute brain slices were collected using a two-photon microscopy (VIVO 2-Photon platform, Intelligent Imaging Innovations GmbH), equipped with a tunable multiphoton laser (MaiTai laser, Spectra-Physics). Acquisition was controlled using Slidebook version 6 software (Intelligent Imaging Innovations GmbH). The average fluorescence for each ROI per frame of the Fluo4 channel was then measured and exported to MATLAB (version R2022a, The Mathworks).<br>IL-2 Immunoassay data was collected using ProQuantun High sensitivity mouse IL2 (Life Technologies)<br>Surface morphology imaging was obtained using a Bioptonics 3001 OPT Scanner.<br><br>Single Cell RNA-seq data: αCamKIIIL-2 analysis code is available in Supplementary Resource 2, and PHP.GFAP-IL-2 analysis code is available in Supplementary Resource 3.
Data analysis	MRI images were processed using the Bruker Biospin software Paravision 6.1.<br>Immunostainings and Functional Imaging were analysed by Image J v2.0.0 or Imaris v.9.5.1.<br>All statistical tests were run using Graphpad Prism 9.<br>Flow data was analysed using FlowJo version 10.7, or by tSNE, FlowSOM and heatmap analysis in R (version 3.6.2) using an in-house script (manuscript in preparation).<br>Single-cell RNA-seq was processed/analyzed with Cell Ranger V.3.1 (αCamKIIIL2 dataset) or V6.0 (PHP.GFAP-IL2 dataset) from 10x Genomics.

March 2021

The resulting count matrices were analyzed with Seurat (https://satijalab.org/seurat/) V.3.1.5 28 (αCamKIIIL2 dataset), or V.4.0.1 and V.4.0.5 (PHP.GFAP-IL2 dataset).
Multi-electrode array electrophysiology recordings were processed and analyzed using Multi Channel Experimenter software (Multi Channel Systems).
Surface morphology imaging was reconstructed by NRecon (v. 1.6.1.6, Bruker) and visualized with Arivis (v. 2.12.5, Rostock).

For manuscripts utilizing custom algorithms or software that are central to the research but not yet described in published literature, software must be made available to editors and reviewers. We strongly encourage code deposition in a community repository (e.g. GitHub). See the Nature Portfolio guidelines for submitting code & software for further information.

Data

Policy information about availability of data

All manuscripts must include a data availability statement. This statement should provide the following information, where applicable:

- Accession codes, unique identifiers, or web links for publicly available datasets
- A description of any restrictions on data availability
- For clinical datasets or third party data, please ensure that the statement adheres to our policy

The data that support the findings of this study are available from the corresponding authors. The scRNA-seq datasets generated in this study are available on GEO as dataset GSE153427 and dataset GSE179176. Source data are provided with this paper.

Field-specific reporting

Please select the one below that is the best fit for your research. If you are not sure, read the appropriate sections before making your selection.

☒ Life sciences ☐ Behavioural & social sciences ☐ Ecological, evolutionary & environmental sciences

For a reference copy of the document with all sections, see nature.com/documents/nr-reporting-summary-flat.pdf

Life sciences study design

All studies must disclose on these points even when the disclosure is negative.

Sample size	Sample sizes were chosen to ensure adequate power with the statistical tests while minimizing the number of animals used in compliance with ethical guidelines. The amount of mice was selected based on power calculations performed based on previous or present studies carried out in our laboratory and in the field.
Data exclusions	No data was excluded from analysis
Replication	Experimental findings were reliably reproduced in at least two independent experiments except when specifically indicated.
Randomization	Age and sex-matched animals were used for each experiment. Animals were also co-housed when possible. No other randomization procedures were used.
Blinding	For the evaluation of all behavior tests, performing clinical score for EAE, performing TBI surgery, photothrombotic Stroke, dMCAO Stroke, the operators were blinded to the experimental groups or genotype of the animals.<br>For imaging assessment of murine samples, immunostainings were analyzed by the experimenter in a blinded manner. Experimenter was unblinded for pooling the data of previously blindingly analyzed independent data.

Reporting for specific materials, systems and methods

We require information from authors about some types of materials, experimental systems and methods used in many studies. Here, indicate whether each material, system or method listed is relevant to your study. If you are not sure if a list item applies to your research, read the appropriate section before selecting a response.

Materials & experimental systems

n/a	Involved in the study	
☐	☒	Antibodies
☒	☐	Eukaryotic cell lines
☒	☐	Palaeontology and archaeology
☐	☒	Animals and other organisms
☒	☐	Human research participants
☒	☐	Clinical data
☒	☐	Dual use research of concern

Methods

n/a	Involved in the study	
☒	☐	ChIP-seq
☐	☒	Flow cytometry
☐	☒	MRI-based neuroimaging

Antibodies

Antibodies used

Immunostaining antibodies:
Anti-Foxp3 (1:500, MAB8214, R&D systems)
Anti-CD4 (1:250, 100506, Biolegend)
Anti-Laminin alpha 4(1:500, AF3837 ,R&D Systems)
Anti-Iba1 (1:1000, 014-19741, Wako)
Anti-GFAP (1:500, ab4674, Abcam)
Anti-CD31 (1:100, MA3105, Invitrogen)
Anti-S100ß (1:1000, S2532, Sigma-Aldrich)
Anti-APC (1:250, ab16794, Abcam)
Anti-NeuN (1:500, ABN90P, Millipore)
Anti-GFP (1:300, 132002, Synaptic Systems)
Anti-GFP (1:1000, 600-401-215, Rockland)
Anti-GFP (1:500, 600-101-215, Rockland)
Anti-GFAP (1:1000, 173004, Synaptic Systems)
Anti-PDGRα (1:200, APA5, BD Pharmingen)
Anti-Aldh1l1 (1:200, ab87117, Abcam)
Anti-MHCII (1:400, 14-5321-82, eBiosciences)
Anti-ZO-1 (1:500, 617300, Invitrogen)
Anti-Claudin-1 (1:200, 51-9000, Thermo Fisher)
Anti-E-cadherin (1:500,610181, BD)
Anti-CD31 (1:100, DIA-310, Dianova)
Anti-Occludin (1:100, 33-1500, Invitrogen)
Alexa Fluor-488 goat anti-rabbit (1:400, A11008, Thermo Fisher)
Alexa Fluor-488 goat anti-mouse (1:400, A11001, Thermo Fisher)
Alexa Fluor-633 goat anti-rat (1:400, A21094, Thermo Fisher)
Hoechst reagent (1:1000, Sigma-Aldrich)

Flow cytometry antibodies:
Alexa Fluor 488 CD25 (PC61.5) eBioscience Cat#53-0251-82 1:100
Alexa Fluor 488 Human/Mouse CCR6 (TC11-18H10) R&D Systems Cat#FAB590G-100 1:50
Alexa Fluor 488 anti-mouse TCR β chain (H57-597) BioLegend Cat#109215 1:2500
Alexa Fluor 700 anti-mouse Ki-67 (16A8) BioLegend Cat#652420 1:500
Alexa Fluor 700 anti-mouse MHC-II I-A/I-E (M5/114) BioLegend Cat#107622 1:500
Amphiregulin Biotinylated Mouse R&D Systems Cat#BAF989 1:500
APC FOXP3 (FJK-16s) eBioscience Cat#17-5773-82 1:200
BB630-P anti-mouse CD80 (16-10A1) BD Biosciences custom 1:2000
BB660-P2 anti-mouse GITR (DTA-1) BD Biosciences Cat#624284 1:10000
BB660-P2 anti-mouse TCRγδ (GL3) BD Biosciences custom 1:1000
BB790-P anti-mouse TCRβ (H57-957) BD Biosciences custom 1:2000
BD Horizon BUV563 Rat Anti-Mouse CD45 (30-F11) BD Biosciences Cat#565710 1:10000
BD Horizon BUV661 Rat Anti-Mouse CD19 (1D3) BD Biosciences Cat#565076 1:1000
BD Horizon BUV737 Rat Anti-Mouse CD62L (MEL-14) BD Biosciences Cat#612833 1:1000
BD Horizon BUV737 rat anti-mouse IFNγ (XMG 1.2), BD Biosciences Cat#564693 1:250
BD Horizon BUV805 Rat Anti-Mouse CD8 (53-6.7) BD Biosciences Cat#612898 1:1000
BD Horizon BV650 Rat Anti-Mouse IL-10 (JES5-16A3) BD Biosciences Cat#564083 1:250
BD OptiBuild BUV395 Rat Anti-Mouse CD103 (M290) BD Biosciences Cat#740238 1:1000
Brilliant Blue 790-P anti-mouse CD45 (30-F11) BD Biosciences custom 1:2000
Brilliant Violet 421 anti-mouse CD152/CTLA4 (UC10-4B9) BioLegend Cat#106312 1:200
Brilliant Violet 570 anti-mouse CD3 (17A2) BioLegend Cat#100225 1:500
Brilliant Violet 570 anti-mouse Ly-6G (1A8) BioLegend Cat#127629 1:2000
Brilliant Violet 570 anti-mouse/human CD44 (IM7) BioLegend Cat#103037 1:1000
Brilliant Violet 605 anti-human/mouse/rat CD278(ICOS) (C398.4A) BioLegend Cat#313538 1:500
Brilliant Violet 605 Streptavidin BioLegend Cat#405229 1:500
Brilliant Violet 650 anti-mouse CD3 (17A2) BioLegend Cat#100229 1:1000
Brilliant Violet 650 anti-mouse CX3CR1 (SA011F11) BioLegend Cat#149033 1:100
Brilliant Violet 711 anti-mouse CD274 (PD-L1) (10F.9G2) BioLegend Cat#124319 1:500
Brilliant Violet 711 anti-mouse CD279 (PD-1) (29F.1A12) BioLegend Cat#135231 1:5000
Brilliant Violet 785 anti-mouse CD19 (6D5) BioLegend Cat#115543 1:200
Brilliant Violet 785 anti-mouse/human CD44 (IM7) BioLegend Cat#103041 1:1000
Brilliant Violet 785 anti-mouse/human KLRG1 (MAFA) BioLegend Cat#138429 1:1000
BUV395 Mouse Anti-Ki-67 (B56) BD Biosciences Cat#564071 1:250
BUV395 Rat Anti-Mouse IL-17A BD (TC11-18H10) Biosciences Cat#565246 1:2000
BUV496 Rat Anti-Mouse CD4 (GK1.5) BD Biosciences Cat#612952 1:500
BUV563 anti-mouse Ly6C (AL-21) BD Biosciences custom 1:2000
BUV615-P Streptavidin BD Biosciences custom 1:1000
BUV737 Hamster Anti-Mouse CD69 (H1.2F3) BD Biosciences Cat#564684 1:200
BV480 Rat Anti-Mouse CD25 (PC61) BD Biosciences Cat#566120 1:1000
BV750 anti-mouse CD11b (M1/70) BD Biosciences custom 1:2000
BV750 Rat Anti-Mouse TNF (MP6-XT22) BD Biosciences Cat#566365 1:100
eFluor 450 IL-1 beta (Pro-form) (NJTEN3), eBioscience Cat#48-7114-82 1:250
Fixable Viability Dye eFluor™ 780 eBioscience™ Cat#65-0865-18 1:2000
PE anti-mouse GM-CSF (MP1-22E9) BioLegend Cat# 505406 1:250

PE CD107a (LAMP-1) (1D4B) eBioscience Cat#12-1071-82 1:5000
PE CD11b (M1/70) eBioscience Cat#12-0112-82 1:2000
PE T-bet (4B10) eBioscience Cat#12-5825-82 1:200
PE T-bet (REA102) Miltenyi Biotec Cat#130-098-596 1:200
Pe-Cy5.5 anti-mouse NK-1.1 (PK136) BioLegend Cat#108702 1:500
PE-Cyanine5 CD11b (M1/70) eBioscience Cat#15-0112-81 1:2000
PE-Cyanine5 TCRγδ (GL3) eBioscience Cat#15-5711-82 1:500
PE-Cyanine5.5 CD3 (145-2C11) eBioscience Cat#35-0031-80 1:5000
PE-Cyanine7 anti-mouse TGFβ / LAP eBioscience Cat#25 9821-80 1:50
PE-Cyanine7 CD45 (30-F11) eBioscience Cat#25-0451-82 1:500
PE-Cyanine7 IL-33R (ST2) (RMST2-2) eBioscience Cat#25-9335-80 1:5000
PE-Vio615 Helios (REA829) Miltenyi Biotec Cat#130-112-636 1:500
PE/Cy5 anti-mouse CD69 (H1.2F3) BioLegend Cat#104510 1:200
PE/Dazzle 594 anti-mouse CD64 (FcγRI) BioLegend Cat#139320 1:500
PE/Dazzle 594 anti-mouse IL-2 (JES6-5H4) BioLegend Cat# 503840 1:250
PerCP-Cyanine5.5 Rat Anti-Mouse CD4 (GK1.5) eBioscience Cat#45-0042-82 1:100
PerCP-eFluor 710 CD304 (Neuropilin-1) (3DS304M) eBioscience Cat#46-3041-82 1:5000
Super Bright 600 IL-33R (ST2) (RMST2-2) eBioscience Cat#63-9335-80 1:500
TrkB Biotinylated Mouse R&D Systems Cat#BAF1494 1:200

Validation

Immunostaining antibodies and Flow cytometry antibodies were validated by manufacturers, and were used based in previous publication (Pasciuto et al., Cell 2020 doi: 10.1016/j.cell.2020.06.026).

Animals and other organisms

Policy information about studies involving animals; ARRIVE guidelines recommended for reporting animal research

Laboratory animals

Both male and female mice (8-12 weeks old) were used in this study, unless otherwise specified in the manuscript. All mice were used on the C57BL/6 background. C57BL/6J (Jackson stock #000664), Foxp3-Cre (MGI:3809724) , αCamKII-CreERT2 (MGI:4439147) , PLP1-CreERT (MGI:2450391) IL-2-GFP mice (MGI:3830507), and Rag1 knockout mice (Jackson stock #002216) were used. RosaIL2 transgenic mice were generated through the insertion of a cassette containing a floxed-STOP sequence followed by an IL2-IRES-GFP sequence into the Rosa26 locus, using the endogenous Rosa26 promoter (White et al, bioRxiv, 2020.2012.2018.423431).

Mice were housed under SPF conditions, under a 12 hour light/dark cycle in a temperature and humidity-controlled room with ad libitum access to food and water.

Wild animals

The study did not involved wild animals.

Field-collected samples

The study did not involved samples collected from the field.

Ethics oversight

All animal procedures were approved by the KU Leuven Animal Ethics Committee (P035/2015, P015/2014, P209/2015, P043/2016, P082/2018, P124/2019,), the University of Amsterdam (CCD 4925, AVD1110020184925), or the Babraham Institute Animal Welfare and Ethics Review Body (PP3981824) taking into account relevant national and European guidelines.

Note that full information on the approval of the study protocol must also be provided in the manuscript.

Flow Cytometry

Plots

Confirm that:

☒ The axis labels state the marker and fluorochrome used (e.g. CD4-FITC).

☒ The axis scales are clearly visible. Include numbers along axes only for bottom left plot of group (a 'group' is an analysis of identical markers).

☒ All plots are contour plots with outliers or pseudocolor plots.

☒ A numerical value for number of cells or percentage (with statistics) is provided.

Methodology

Sample preparation

Mice were deeply anaesthetized with ketamine/xilazine i.p. and transcardially perfused with ice cold PBS unless otherwise state. Mouse tissues have been harvested in RPMI 1640 (Life-technologies) supplemented with 2 mM MgCl2, 2 mM CaCl2, 20% FBS, 2 mM EDTA and 2 mM HEPES and kept in ice until processing. Single-cell suspensions were prepared from mouse, spleen, and lymph nodes by mechanical dissociation or from blood by red blood cells lysis. Single cell suspensions from brain tissue were prepared by digestion for 30 minutes at 37ºC with 2 mg/ml collagenase D (Roche), 300 µg/ml hyaluronidase (Sigma-Aldrich) and 40 ug/ml DNase I (Sigma-Aldrich) in RPMI supplemented with 2mM MgCl2, 2mM CaCl2 20% FBS and 2 mM HEPES. Digested tissue was mechanically disrupted, filtered through 100µm mesh and enriched for leukocytes by centrifugation (600g, 10 minutes, no brakes) through 30% Percoll (GE Healthcare). Non-specific binding was blocked using 2.4G2 supernatant for mouse cells, and dead cells were labelled by fixable viability dye eFluor 780 (ThermoFisher). Cells were fixed and permeabilised with the eBioscience Foxp3 staining kit (eBioscience).

Instrument	BD Symphony A5 or Cytek Aurora Spectral Analyser were used to collect data for analysis.<br>BD FACSAria III was used for cell sorting.
Software	All flow data was collected using FACSDIVA versions 8.0.2 (BD) and analyzed using FlowJo version 10.7.1 or in-house R script using FlowSOM, tSNE, and heatmap analysis.
Cell population abundance	All sorts had a purity > 95%.
Gating strategy	FSC-A SSC-A was used to gate on cells.<br>FSC-H/FSC-A and SSC-H SSC-A were used to gate on singlet cells.<br>Leucocytes were gated as as Live/CD11b-/CD45+/ Live/CD11b-/CD45+. T cells were gated on Live/CD11b-/CD45+/CD3+. CD4 T conventional cells were gated as Live/CD11b-/CD45+/CD3+/CD19-/CD8-/CD4+/Foxp3-. Regulatory T cells cells were gated as Live/CD11b-/CD45+/CD3+/CD19-/CD8-/CD4+/Foxp3+. CD8 T cells were gated as Live/CD11b-/CD45+/CD3+/CD19-/CD8+/CD4-. NK cells cells were gated as Live/CD11b-/CD45+/NK1.1. Microglia were gated as as Live/CD11b+/CD45int/ CD3-/CD19-/Ly6G-/CX3CR1+/CD64+.

☒ Tick this box to confirm that a figure exemplifying the gating strategy is provided in the Supplementary Information.

Magnetic resonance imaging

Experimental design

Design type	Assessment of brain injury after MCAO and TBI using volumetric measurements as well as T2 relaxation times and ADC values
Design specifications	T2-weighted anatomical MRI; T2 map (multislice-multi-echo, 12 TE increments); ADC map (6 b-values plus b=0)
Behavioral performance measures	Control of physiological parameters (body temperature and respiration rate)

Acquisition

Imaging type(s)	Structural MRI (2D T2-weighted (spin echo) as well as 3D MRI (gradient echo); parametric T2-map (multi-slice-multi echo, 12 TE increments of 12ms); diffusion MRI (ADC map with 6 b-values between 100 to 1500 plus b=0)
Field strength	9.4 Tesla
Sequence & imaging parameters	Spin echo for structural MRI (TE=40ms, TR=4.5s, RARE factor 8, 24 slices of 0.5mm thickness)<br>3D gradient echo MRI (FLASH, TR=30ms, TE=7ms, 20dgr flip angle, 0.125mm isotropic resolution)<br>T2 maps (MSME sequence, 20 slices of 0.5mm thickness, TR=4s, 12 TE increments of 12ms)<br>ADC maps (TR=2s, TE=20ms, same geometry as for T2 maps, b-values=0, 100, 300, 500, 800, 1000, 1500
Area of acquisition	coverage of the whole brain (excluding parts for the olfactory bulb and parts of the cerebellum)

Diffusion MRI ☒ Used ☐ Not used

Parameters: 1 direction, b-values=0, 100, 300, 500, 800, 1000, 1500, no cardiac gating

Preprocessing

Preprocessing software	Paravision v6.0.1 (Bruker Biospin)
Normalization	parametric T2 and ADC maps using exponential fitting of SI vs. TE/ b-value incrementation, respectively
Normalization template	none
Noise and artifact removal	none
Volume censoring	*Define your software and/or method and criteria for volume censoring, and state the extent of such censoring.*

Statistical modeling & inference

Model type and settings	no fMRI data
Effect(s) tested	*Define precise effect in terms of the task or stimulus conditions instead of psychological concepts and indicate whether ANOVA or factorial designs were used.*

Specify type of analysis: ☒ Whole brain ☐ ROI-based ☐ Both

Statistic type for inference<br>(See Eklund et al. 2016)	*Specify voxel-wise or cluster-wise and report all relevant parameters for cluster-wise methods.*

Correction

Describe the type of correction and how it is obtained for multiple comparisons (e.g. FWE, FDR, permutation or Monte Carlo).

Models & analysis

n/a	Involved in the study
☒	☐ Functional and/or effective connectivity
☒	☐ Graph analysis
☒	☐ Multivariate modeling or predictive analysis
